# Measuring organizational and individual factors thought to influence the success of quality improvement in primary care: a systematic review of instruments

**DOI:** 10.1186/1748-5908-7-121

**Published:** 2012-12-17

**Authors:** Sue E Brennan, Marije Bosch, Heather Buchan, Sally E Green

**Affiliations:** 1 School of Public Health and Preventive Medicine, Monash University, Melbourne, Australia; 2 Central Clinical School, Monash University and National Trauma Research Institute, Melbourne, Australia; 3 Australian Commission on Safety and Quality in Health Care (ACSQHC), Sydney, Australia

**Keywords:** Continuous quality improvement, Primary care, Evaluation, Systematic review, Measurement, Instrument, Conceptual framework, Theoretical model, Intervention fidelity, Organizational context

## Abstract

**Background:**

Continuous quality improvement (CQI) methods are widely used in healthcare; however, the effectiveness of the methods is variable, and evidence about the extent to which contextual and other factors modify effects is limited. Investigating the relationship between these factors and CQI outcomes poses challenges for those evaluating CQI, among the most complex of which relate to the measurement of modifying factors. We aimed to provide guidance to support the selection of measurement instruments by systematically collating, categorising, and reviewing quantitative self-report instruments.

**Methods:**

Data sources: We searched MEDLINE, PsycINFO, and Health and Psychosocial Instruments, reference lists of systematic reviews, and citations and references of the main report of instruments. Study selection: The scope of the review was determined by a conceptual framework developed to capture factors relevant to evaluating CQI in primary care (the InQuIRe framework). Papers reporting development or use of an instrument measuring a construct encompassed by the framework were included. Data extracted included instrument purpose; theoretical basis, constructs measured and definitions; development methods and assessment of measurement properties. Analysis and synthesis: We used qualitative analysis of instrument content and our initial framework to develop a taxonomy for summarising and comparing instruments. Instrument content was categorised using the taxonomy, illustrating coverage of the InQuIRe framework. Methods of development and evidence of measurement properties were reviewed for instruments with potential for use in primary care.

**Results:**

We identified 186 potentially relevant instruments, 152 of which were analysed to develop the taxonomy. Eighty-four instruments measured constructs relevant to primary care, with content measuring CQI implementation and use (19 instruments), organizational context (51 instruments), and individual factors (21 instruments). Forty-one instruments were included for full review. Development methods were often pragmatic, rather than systematic and theory-based, and evidence supporting measurement properties was limited.

**Conclusions:**

Many instruments are available for evaluating CQI, but most require further use and testing to establish their measurement properties. Further development and use of these measures in evaluations should increase the contribution made by individual studies to our understanding of CQI and enhance our ability to synthesise evidence for informing policy and practice.

## Background

Continuous quality improvement (CQI) approaches are prominent among strategies to improve healthcare quality. Underpinned by a philosophy that emphasises widespread engagement in improving the systems used to deliver care, CQI teams use measurement and problem solving to identify sources of variation in care processes and test potential improvements. The use of iterative testing (plan-do-study-act cycles) by QI teams to design and implement an evidence-based model of depression care is one example [1,2]. CQI methods have been used as the main strategy in organisation-wide quality improvement (QI) efforts [3-5], as a tool for implementing specific models of care [1,6], and as the model for practice change in QI collaboratives [7]. Investment in CQI-related education reflects the increasing emphasis on these methods, with inclusion of QI cycles as modules in continuing medical education curricula [8] and incorporation of CQI principles as core competencies for graduate medical education [9].

Despite this widespread emphasis on CQI, research is yet to provide clear guidance for policy and practice on how to implement and optimise the methods in healthcare settings. Evidence of important effects and the factors that modify effects in different contexts remains limited [3,4,10-12]. This is particularly the case for primary care, where far less research has been conducted on CQI than in hospital settings [12,13]. Recent calls to address gaps in knowledge have focused on the need for methodological work to underpin evaluations of QI interventions [14-16]. Priority areas include theory development to explain how CQI works and why it may work in some contexts and not others, and the identification of valid and reliable measures to enable theories to be tested [14].

The extent to which specific contextual factors influence the use of CQI methods and outcomes in different settings is not well understood [4,11,17,18]. Measuring organizational context in CQI evaluations is key to understanding the conditions for success, and for identifying factors that could be targeted by CQI implementation strategies to enhance uptake and effectiveness [3,11,17]. In intervention studies, measuring these factors as intermediate outcomes permits investigation of the mechanisms by which CQI works. Measuring the extent to which CQI methods are used in practice is uncommon in evaluative studies [13], but provides important data for interpreting effects. Complex interventions such as CQI are not easily replicable or implemented in a way that ensures that intervention components are used as intended [19]. Moreover, adaptation to fit the local context may be necessary [17,20]. Measures that capture the implementation and use of CQI interventions are required to assess whether observed effects (or the absence thereof) can be attributed to the intervention. These measures of intervention fidelity also permit assessment of the extent to which individual intervention components contribute to effects and whether changes to the intervention have an important influence on effects [17,20,21].

Investigating the relationship between context, use of CQI, and outcomes poses practical and methodological challenges for researchers. These challenges include determining which factors to measure and selecting suitable measurement instruments from a large and complex literature. Variability in how contextual factors have been defined and measured adds to these challenges and limits the potential to compare and synthesise findings across studies [12].

In this paper, we report a systematic review of instruments measuring organizational, process, and individual-level factors thought to influence the success of CQI. This review is part of a larger project that aims to aid the evaluation of CQI in primary care by providing guidance on factors to include in evaluations and the measurement of these factors. The project includes a measurement review (reported in two parts; this paper and a companion review focussing on team-level measures) and development of a conceptual framework, the Informing Quality Improvement Research (InQuIRe) in primary care framework. Our initial framework is included in this paper to illustrate the scope of the measurement review and as the basis for assessing the coverage of available instruments. Our analysis of instruments is used to integrate new factors and concepts into the framework. These refinements are reported as taxonomies in the measurement review papers. The development and content of the final InQuIRe framework will be reported in full in a separate publication.

The specific objectives of the measurement review reported in this paper are to: identify measures of organizational, CQI process, and individual factors thought to modify the effect of CQI; determine how the factors measured have been conceptualised in studies of QI and practice change; develop a taxonomy for categorising instruments based on our initial framework and new concepts arising from the measurement review; use the taxonomy to categorise and compare the content of instruments, enabling assessment of the coverage of instruments for evaluating CQI in primary care; and appraise the methods of development and testing of existing instruments, and summarise evidence of their validity, reliability, and feasibility for measurement in primary care settings.

### Scope of the review—the InQuIRe framework

**Figure 1:**
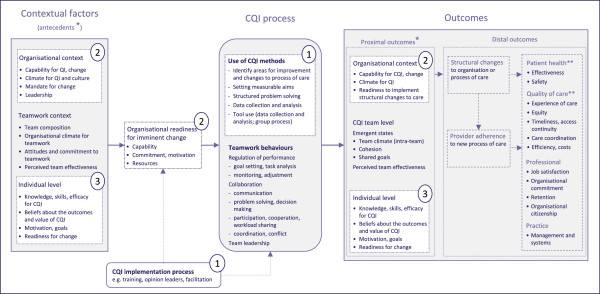
**Conceptual framework for defining the scope of the review – Informing Quality Improvement Research (InQuIRe) in primary care.** Instruments within the scope of the review reported in this paper cover three content domains (shaded in white and numbered as follows in the figure and throughout the review): (1) CQI use and implementation; (2) Organizational context; (3) Individual level factors. Boxes shaded in grey are included in a companion paper reporting team measures. Boxes with dashed lines are outside the scope of either review. * Contextual factors that are potentially modifiable by participation in the CQI process are depicted as both antecedents and proximal outcomes. ** Primarily based on dimensions of quality from Institute of Medicine (U.S.) Committee on Quality of Health Care in America. Crossing the quality chasm: a new health system for the 21st century. Washington DC: National Academy Press, 2001:xx, 337.

Figure 1 depicts the first version of our InQuIRe framework, which we used to set the scope of this measurement review. Development of the InQuIRe framework was prompted by the absence of an integrated model of CQI theory for informing the design of evaluations in primary care. The version of InQuIRe presented in Figure 1 reflects our initial synthesis of CQI theory, models, and frameworks. It aims to capture the breadth of factors that could be measured when evaluating CQI in primary care settings.

The starting point for our synthesis was the landmark papers that spurred the adoption of CQI in healthcare (*e*.*g*., [22-29]). From these sources, we identified recurrent themes about the core components of CQI and how it was expected to work. We used snowballing methods to uncover the main bodies of research (including reviews) and prevailing theory on CQI in healthcare. This literature focussed on large organizational settings (*e*.*g*., [10,30-34]) with few models for primary care and limited consideration of team-level factors in CQI theory (exceptions include [1,35,36]). We therefore extended our search to identify more general models or theories of QI, practice change, and innovation relevant to primary care (examples in primary care are Cohen’s model for practice change based on complexity theory [37], Orzano’s model of knowledge management [38], and Rhydderch’s analysis of organizational change theory [39]; in other settings [40-42]), and review articles on teamwork theory (*e*.*g*., [43-47]). Factors salient to CQI in primary care were collated and grouped thematically to identify content for our framework. O’Brien and Shortell’s model of organizational capability for QI [30], and Solberg’s conceptual framework for practice improvement [48] were among the few models that integrated findings across CQI studies to describe relationships between context and outcomes. We used these models as the initial basis for our framework, integrating findings from our thematic analysis of other sources.

To structure our framework, we adopted the inputs-process-outputs (IPO) model that is widely used in research on teams [46]. Although it simplifies the relationship between variables, the IPO model depicts variables in a way that supports the design and interpretation of longitudinal studies. Reporting available instruments using this structure illustrates how the instruments included in this review could be incorporated in an evaluation of the effects of CQI. Contextual factors thought to influence CQI process, and outcomes are presented as antecedents of organizational readiness for change. Organizational readiness, defined here as collective capability and motivation for an imminent change [40], is hypothesised to mediate the effects of contextual factors on CQI process and outcomes. This is consistent with the view that organizational readiness should be delineated from other contextual factors that make an organisation receptive to change, but which do not reflect an organisation’s readiness to engage in a specific change [41,49,50]. Contextual factors that are potentially modifiable are depicted in the framework as both antecedents and proximal outcomes. These factors may be modified by participation in the CQI process itself or by methods used to implement CQI (*e*.*g*., improving motivation for CQI by using opinion leaders). In turn, proximal outcomes may mediate the effect of CQI process on more distal outcomes (*e*.*g*., structural changes to the process of care, and provider adherence to these changes). Our concept of CQI process focuses on the use of CQI methods most salient to primary care settings [51]. These methods are reflected in Weiner’s operational definition of CQI ‘use of cross-functional teams to identify and solve quality problems, use of scientific methods and statistical tools by these teams to monitor and analyse work processes, and use of process-management tools …’ [52].

**Figure 2:**
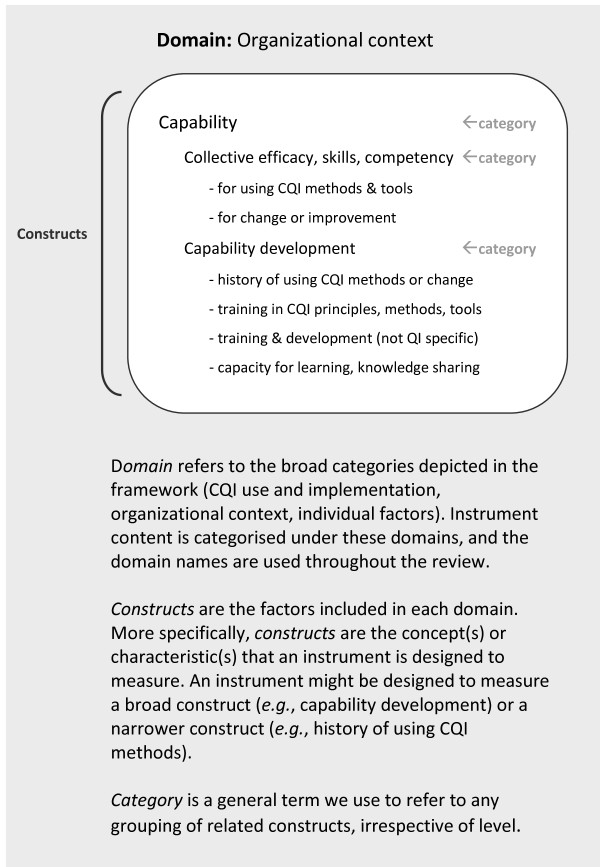
Terms used to describe the taxonomy, illustrated with content from the ‘Capability for QI or change’ category of the ‘Organizational context’ domain.

This review focuses on instruments relevant to three domains of the InQuIRe framework (shaded in white and numbered one to three as follows). Broadly, these cover: (1) CQI implementation and use (*i*.*e*., measures of the process used to implement CQI and the fidelity with which CQI methods are used); (2) organizational context (*e*.*g*., technical capability for CQI and organizational culture); and (3) individual level factors (*e*.*g*., knowledge and beliefs about CQI). Figure 2 illustrates terms used throughout the review, with an example from the taxonomy (see Additional file 1 for a glossary of these and other terms).

## Methods

**Figure 3:**
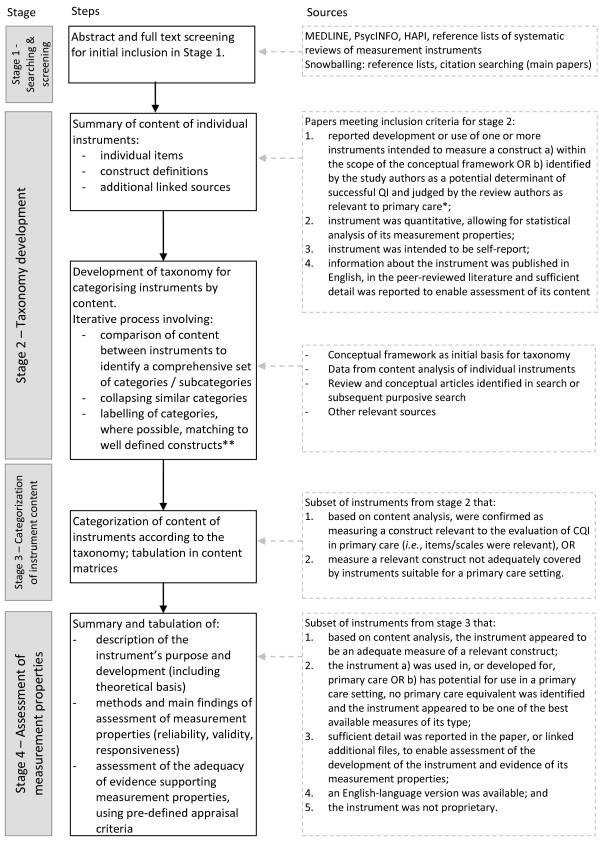
**Stages of data extraction and analysis for the review.** * External factors (e.g., financing, accreditation) were excluded as these are likely to be specific to the local health system. ** Extent to which this was possible depended on the existence of consistent construct definitions in multiple included studies or, alternatively, in synthesized sources from the extant literature (i.e., recent or seminal review article or meta-analysis).

Methods for the review of measurement instruments are not well established [53]. Figure 3 summarises the stages of this review. Searching and screening (stage one) followed general principals for the conduct of systematic reviews, while data analysis and synthesis methods (stages two to four) were developed to address the objectives of this review. The methods used at each stage of the review are described below.

### Stage one: searching and initial screening

#### Data sources and search methods

To identify papers reporting potentially relevant instruments we searched MEDLINE (from 1950 through December 2010), PsycINFO (from 1967 through December 2008), and Health and Psychosocial Instruments (HaPI) (from 1985 through September 2008) using controlled vocabulary (thesaurus terms and subject headings) and free-text terms for quality improvement and practice change. Scoping searches were used to test terms (*e*.*g*., to test retrieval of known reports of instruments) and gauge the likely yield of references. Further details about the scoping searches and the final set of search terms are reported in Additional file 2. Searches were limited to articles published in English language.

Reports of potentially relevant instruments were also identified from systematic reviews identified from the database searches and other sources. These reviews included systematic reviews of measurement instruments, and systematic reviews of QI studies (*e*.*g*., reviews of observational studies measuring factors thought to influence QI outcomes).

Snowballing techniques were used to trace the development and use of instruments and to identify related conceptual papers. We identified the main publication(s) reporting initial development of instruments, screened the reference lists of these studies, and conducted citation searches in ISI Web of Science citation databases or Scopus for more recent publications [54]. Snowballing searches were limited to the subset of instruments included in stage four of the review.

#### Selection of studies for initial inclusion in the review

Titles and abstracts were screened to identify studies for inclusion in the review. Clearly irrelevant papers were excluded and the full text of potentially relevant studies was retrieved and screened for inclusion by one author (SB). Criteria for the selection of studies included in stage two are reported in Figure 3.

### Stage two: development of taxonomy for categorising instruments

#### Data extraction

One review author extracted data from included studies for all three stages (SB). To ensure consistent interpretation of the data extraction guidance and data extraction, a research assistant extracted data from a subsample of included studies (18 instruments, comprising 10% of the instruments included in stage two and 25% of the instruments included in stage four). Data extracted at stage two are summarised in Table 1. This data included information on the purpose and format of the instrument, and data to facilitate analysis and categorisation of the content of each instrument (constructs measured, construct definitions, theoretical basis of instrument).

**Table 1:** Data extracted at stage two

**Data extracted**	**Description**
Study characteristics	Study aims
	Study design (categorised as experimental, observational, instrument development, model development)
	Setting in which the instrument was used
Instrument source	Name of instrument
	Source paper for the instrument as cited by the authors
Instrument purpose	Purpose for which the instrument was used (descriptive, predictive or diagnostic, outcome measure/evaluative)
Instrument format	Number of items
	Response scale (Likert, ipsative, *etc*.); response options
Instrument content and theoretical basis	Constructs and dimensions measured
	Definitions of the constructs; additional description of the content required to illustrate how the construct had been operationalized (*e*.*g*., sample items)
	Theoretical basis of the instrument and references cited for the theory

#### Taxonomy development

Methods for developing the taxonomy were based on the framework approach for qualitative data analysis [55]. This approach combines deductive methods (commencing with concepts and themes from an initial framework) with inductive methods (based on themes that emerge from the data). The first version of the InQuIRe framework (Figure 1) was the starting point for the taxonomy, providing its initial structure and content. Content analysis of the instruments included in stage two was used to identify factors that were missing from our initial framework (and hence, the taxonomy) (*e*.*g*., we added commitment, goals, and motivation as organisation level-constructs, when in our initial framework they were included only at individual-level) and to determine how factors had been conceptualised. The initial taxonomy was revised to incorporate new factors and prevailing concepts (*e*.*g*., we separated dimensions of climate that were prevalent in instruments specific to QI (*e*.*g*., emphasis on process improvement), from more general dimensions of climate (*e*.*g*., cooperation)). Using this approach enabled us to ensure the taxonomy provided a comprehensive representation of relevant factors.

Instruments confirmed as relevant to one or more of the three domains of our framework were included for content analysis. At this stage, we were aiming to capture the breadth of constructs relevant to evaluating CQI. Hence, we included all measures of potentially relevant constructs irrespective of whether item content was suitable for primary care. The content of each instrument (items, subscales), and associated construct definitions, was compared with the initial taxonomy. Instrument content that matched constructs in the taxonomy was summarised using existing labels. The taxonomy was expanded to include missing constructs and new concepts, initially using the labels and descriptions reported by the instrument developers.

To ensure the taxonomy was consistent with the broader literature, we reviewed definitions extracted from review articles and conceptual papers identified from the search. We also searched for and used additional sources to define constructs when included studies did not provide a definition, a limited number of studies contributed to the definition, or the definition provided appeared inconsistent with the initial framework or with that in other included studies. Following analysis of all instruments and supplementary sources, related constructs were grouped in the taxonomy (as illustrated in Figure 2). Overlapping constructs were then collapsed, distinct constructs were assigned a label that reflected the QI literature, and the dimensions of constructs were specified to create the final taxonomy.

### Stage three: categorisation of instrument content

Criteria for the selection of the subset of instruments included in stage three are reported in Figure 3. Categorisation of instrument content was primarily based on the final set of items reported in the main report(s) for each instrument. Construct definitions and labels assigned to scales guided but did not dictate categorisation because labels were highly varied and often not a good indicator of instrument content (*e*.*g*., authors used the following construct labels for very similar measures of QI climate: organizational culture that supports QI [56], organizational commitment to QI [57], QI implementation [58], degree of CQI maturity [59], quality management orientation [60], and continuous improvement capability [61]). Instrument content was summarised in separate tables for each of the content domains from the InQuIRe framework: (1) CQI implementation and use, (2) organizational context, and (3) individual level factors.

### Stage four: assessment of measurement properties

Criteria for the selection of instruments included in stage four are reported in Figure 3.

#### Data extraction

We extracted information about the development of the instrument and assessment of its measurement properties from the main and secondary reports. Secondary reports were restricted to studies of greatest relevance to the review, focussing on studies of CQI, QI, or change in primary care. Table 2 summarises the data extracted at stage four. Extracted data was summarised and tabulated, providing a brief description of the methods and findings of assessments that were of most relevance to this review.

**Table 2:** Data extracted at stage four

**Data extracted**	**Description**^**1**^
Instrument development	Methods used to generate items (*e*.*g*., items derived from existing instruments; new items generated from data from interviews or comprehensive review of theory)
	Methods used to refine instrument
Administration & scoring	Method of administration (*e*.*g*., self-administered, facilitated)
	Feasibility of administration (*e*.*g*., researcher time, resources)
	Acceptability to respondents (*e*.*g*., views on burden and complexity)
	Methods of scoring and analysis
Measurement properties	Methods and findings of assessments of:
	Content validity (*e*.*g*., clear description of content domain and theoretical basis, expert assessment of items for relevance and comprehensiveness)
	Construct validity
	- Instrument structure (*e*.*g*., using factor analytic methods)
	- Hypothesis testing (*e*.*g*., whether scores on the instrument converge with measures of theoretically related variables, discriminate between groups, predict relevant outcomes)
	Reliability (*e*.*g*., internal consistency, stability over time, inter-rater)
	Responsiveness
Other assessments	Interpretability (potential for ceiling and floor effects; guidance on what constitutes an important change or difference in scale scores)
	Generalizability (sampling methods, description of sample, and response rate reported)

^1^ Definitions and further explanation of the extracted measurement properties are provided in Additional file 3.

#### Appraisal of evidence supporting measurement properties

Studies included in stage four were appraised using the COSMIN (COnsensus-based Standards for the selection of health status Measurement Instruments) checklist [62]. The COSMIN checklist focuses on the appraisal of the methods used during instrument development and testing, not on the measurement properties of the instrument itself. The COSMIN criteria were intended for studies reporting instruments for the measurement of patient reported outcomes; however, we were unable to identify equivalent appraisal criteria for organizational measures [63]. The checklist has strong evidence for its content validity, having been derived from a systematic review and an international consensus process to determine its content, terminology, and definitions [62,64]. The terminology and definitions in the COSMIN checklist closely match those adopted by the Joint Committee on Standards for Educational and Psychological Testing [65], indicating their relevance to measures other than health outcomes. Based on guidance from the organizational science and psychology literature [66-68], and other reviews of organizational measures (*e*.*g*., [40,63,69]), we added a domain to address issues associated with the measurement of collective constructs (level of analysis) and a criterion to the content validity domain. The appraisal criteria are reported in Additional file 3.

Most instruments had undergone limited testing of their measurement properties and, where properties had been tested, there was often limited reporting of the information required to complete the checklist. Because of the sparse data, for each instrument we tabulated a summary of the extent of evidence available for each property and a description of the instrument’s development and testing. We used appraisal data to provide an overall summary of the methods used to develop and test the measurement properties of instruments included in the review.

## Results

### Summary of initial screening process for the review

**Figure 4:**
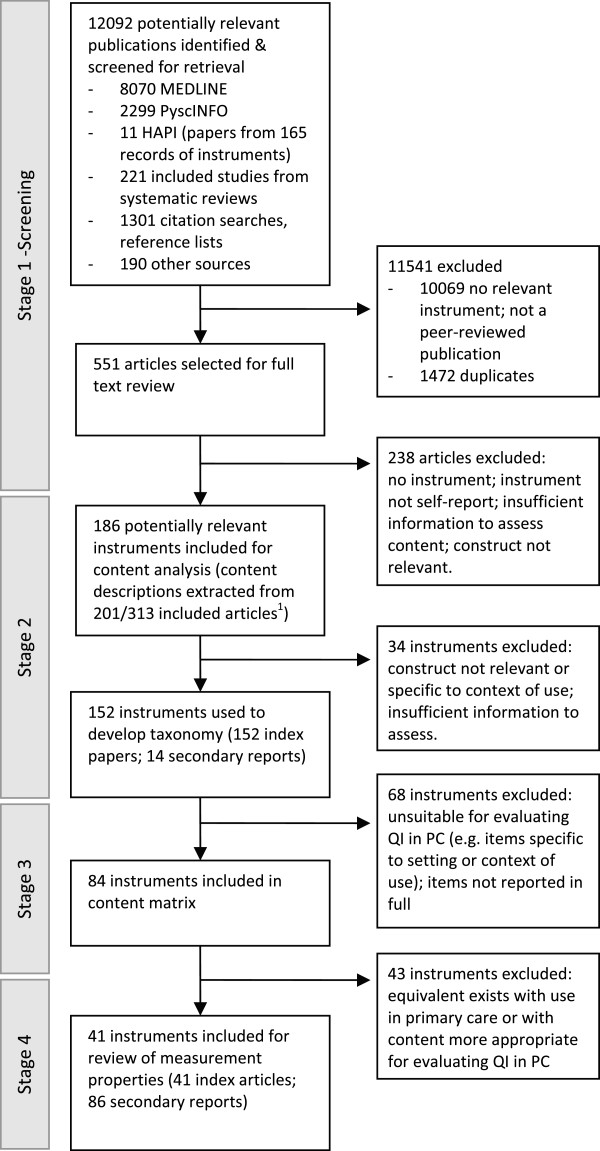
**Flow of studies and instruments through the review.**^1^Remainder of 313 articles (n =112) were secondary reports that did not contribute additional information about instrument content. These were retained for assessment of measurement properties if required when final set of studies for inclusion in stage four was determined.

Figure 4 summarises the flow of studies and instruments through the review. A total of 551 articles were included for full text review. This included eight systematic reviews of instruments (two on readiness for change [40,69], three on organizational culture [63,70,71], and one each on quality improvement implementation [72], organizational assessment [73], and organizational learning [74]); and five systematic reviews of observational or effectiveness studies [12,75-78]. Ninety-one articles were identified from the systematic reviews of instruments, and 60 articles were identified from the other reviews.

Of the 313 papers included for the first stage of data extraction, the majority reported studies in healthcare settings (n = 225), 83 of which were in primary care. Of the included papers, 62 had as their primary aim development of an instrument. Observational designs were most commonly reported in other papers (n = 196), encompassing simple descriptive studies through to testing of theoretical models. Experimental designs were reported in 25 papers, of which five were randomised trials (four in primary care), one a stepped-wedge time series design, and the balance were before-after designs. The remainder of papers were conceptual, including qualitative studies and descriptive papers.

### Identification of unique instruments

Individual papers reported between one and four potentially relevant instruments; collectively providing 352 unique reports of development or use of an instrument. One hundred and eighty-six unique, potentially relevant instruments were identified, 34 of which were excluded following initial analysis of the content of all instruments (constructs measured, items), resulting in 152 instruments for review. Reasons for exclusion are reported in Figure 4. Identification of the main and secondary reports required direct comparison of items with previously identified instruments because of attribution of the same instrument to different sources or no source reference, use of different names for the same instrument, and changes to content without reporting that changes had been made. Most instruments were unnamed or had multiple names reported in the literature. We therefore used the first author’s name and year from the index paper to name the instrument (reflected in text and tables, *e*.*g*., Solberg 2008). The index paper for the instrument was typically the first of the main reports.

### Development of taxonomy and categorisation of instrument content

In stage two, the content of 152 instruments was analysed to inform development of the taxonomy. The breadth of constructs measured and diversity of items used to operationalise constructs was large. Of the 152 instruments, 28 were initially categorised as measuring use or implementation of CQI, 101 measured attributes of organizational context (31 context for CQI or total quality management (TQM)), 46 context for any change or improvement, 17 organizational culture, 7 generic context), 23 measured organizational or individual readiness for change, and 25 individual level factors (some instruments covered more than one domain, hence the total sums to >152). The taxonomy incorporates additional constructs identified during the analysis, makes explicit the dimensions within constructs, and includes some changes to terminology to reflect existing instruments.

At stage three, 84 instruments were categorised using the taxonomy. We included instruments confirmed as measuring a relevant construct with item wording suitable for primary care (41 instruments). These included instruments requiring minor rewording (*e*.*g*., ‘hospital’ to ‘practice’). For constructs not adequately covered by suitable instruments, we included instruments with potential for adaptation (43 instruments). Sixty-eight instruments were excluded from stage three because the instrument content was unsuitable for evaluating QI in primary care (n = 48) or the authors reported only a subset of items from the instrument (*e*.*g*., example questions) (n = 20). Instruments judged as unsuitable were those with content intended for a specific context of use (*e*.*g*., Snyder-Halpern’s instrument measuring readiness for nursing research programs [79]; Lin’s instrument measuring climate for implementing the chronic care model [80]), with content intended for large, differentiated settings (*e*.*g*., Saraph’s instrument measuring quality management factors [81]), or with content adequately covered by more suitable instruments (*e*.*g*., Chen’s instrument measuring generalised self-efficacy [82] was excluded because we identified multiple instruments measuring self-efficacy for CQI (categorised as beliefs about capability)).

The categorisation of instruments is presented in Additional file 4: Tables S3-S6. Each table covers a separate domain of the InQuIRe framework. Instruments are grouped by setting to illustrate if they have been used in primary care or if their use has been limited to other settings. The tables enable comparison across instruments and give an overall picture of coverage of the framework. Instruments vary, however, in how comprehensively they measure individual constructs, some providing comprehensive measures (*e*.*g*., Oudejans, 2011 [83] includes 36 items in 5 scales measuring capacity for learning) while others include only one or two items (*e*.*g*., Apekey, 2011 [84] includes 3 items measuring capacity for learning). Instruments also vary considerably in item wording, influencing their suitability for different purposes. For example, some instruments ask about prior experience of change (*i*.*e*., retrospective measurement) while others refer to an imminent change (*i*.*e*., prospective measurement). In the next section, we include a brief description of each domain of the InQuIRe framework and highlight instruments that provide good coverage of specific constructs. Used in conjunction with the results tables, this information can help guide the selection of instruments.

### Content and coverage of domains of the InQuIRe framework

#### 1) CQI implementation and use

Additional file 4: Table S3 reports the final taxonomy and categorisation of instrument content for the CQI implementation and use domain (boxes numbered ‘1’ in InQuIRe framework).

##### Description of the CQI implementation and use domain

This domain covers the process used to implement CQI (*e*.*g*., training in the use of PDSA cycles, facilitation to help teams apply QI tools, influencing acceptability of CQI as a method for change), organisation wide use of CQI methods (*e*.*g*., process improvement, use of teams for QI), and the use of CQI methods by QI teams (*e*.*g*., planning and testing changes on a small scale, as done in plan-do-study-act (PDSA) cycles). We adopted Weiner’s operational definition of CQI methods ‘use of cross-functional teams to identify and solve quality problems, use of scientific methods and statistical tools by these teams to monitor and analyse work processes, and use of process-management tools …’ [52]. Instruments that focussed on organizational policies or practices used to support CQI (*e*.*g*., leadership practices) were categorised under organizational context (boxes numbered ‘2’ in the InQuIRe framework). We view these instruments as measures of climate for QI rather than measures of the use of CQI methods. This is in line with prevailing definitions of climate as ‘the policies, practices, and procedures as well as the behaviours that get rewarded, supported, and expected in a work setting’ [85]. It is also consistent with recent attempts to identify an operational definition for CQI interventions, which focused on CQI methods such as the use of data to design changes [86].

Instrument content was categorised as CQI implementation process, organisation-wide use of CQI methods, and use of CQI methods by QI teams. Our concept of the CQI implementation process extends our initial framework by drawing on the analysis of instrument content and review articles (key reviews were [87,88]). Organisation-wide use of CQI methods covers indicators of the use of CQI methods across an organisation [52,89]. The use of CQI methods by QI teams encompasses the main components of CQI depicted in our initial framework (*e*.*g*., setting aims, structured problem solving, data collection and analysis, use of QI tools) [51,90-92].

##### Measures of the CQI implementation process

Duckers 2008 [93] and Schouten 2010 [94] included items measuring methods used to implement CQI, with Schouten 2010 providing a more comprehensive measure of training, facilitation and opinion leader support. Three instruments measured processes used to implement change, but these were not specific to CQI (Gustafson 2003 [42], Helfrich 2009 [95], and Øvretveit 2004 [96]). The instruments were primarily included in the review as measures of organizational context; however, their content is relevant to measuring the process used to implement CQI and the theoretical basis of these instruments is strong.

##### Measures of the use of CQI methods – organizational and team level

Barsness 1993 [89] was the most widely used indicator of organisation-wide use of CQI methods (*e*.*g*., [97,98]), and the only instrument suitable for smaller healthcare settings. Of the instruments included as measures of the use of CQI methods at team level, most involved dichotomous responses to whether methods were used or not, or rating of frequency of use of CQI methods (*e*.*g*. Solberg 1998 [56], Lemieux-Charles 2002 [35], Apekey 2011 [84]). We did not identify any comprehensive self-report instruments for measuring the fidelity with which CQI methods are used, such as measures of the intensity of use of CQI methods. Alemni 2001 [99] was the most comprehensive measure of fidelity, but included response formats that would require modification for use as a quantitative scale. Two instruments developed for QI collaboratives measured the use of CQI methods (Duckers 2008 [93] and Schouten 2010 [94]), of which Schouten 2010 was the most comprehensive.

#### 2) Organizational context

Additional file 4: Tables S4 and S5 reports the final taxonomy and categorisation of instrument content for the organizational context domain (boxes numbered ‘2’ in the InQuIRe framework).

##### Description of the organizational context domain

We included instruments in this domain if they measured perceptions of organizational: capability; commitment, goals and motivation; climate for QI or change; generic climate; culture; leadership for QI; resources, supporting systems and structure; and readiness for change. Our concept of each of these categories is reflected in the taxonomy in Additional file 4: Table S4. Capability and commitment reflect perceptions of the collective expertise and motivation to undertake CQI [18,30,48]. We distinguish organizational climate (defined in the previous section) from culture, the latter reflecting ‘… core values and underlying ideologies and assumptions …’ in an organisation [100]. In the taxonomy, we delineate dimensions of climate for QI and change reflecting models of QI (*e*.*g*., [18,30,37,48]). We adopt a broad definition of leadership for QI, ‘any person, group or organisation exercising influence’ [101], including formal and informal leaders at all levels.

##### Measures of capability and commitment

Organisation-level measures of capability and commitment to using CQI methods were uncommon, despite their potential importance as indicators of organizational readiness for CQI [40]. A number of instruments were labelled as measures of organizational commitment to CQI; however, these instruments focussed on practices and policies that reflected management commitment rather than the collective commitment of staff within the organisation. Although not specific to CQI, several instruments designed for primary care included items measuring capability for change and commitment to change (*e*.*g*., Bobiak 2003 [102]; Ohman-Strickland 2007 [103]). Other relevant instruments included those measuring organizational capacity for learning—a dimension of capability (for examples in primary care, Rushmer 2007 [104], Sylvester 2003 [105]).

##### Measures of climate, culture and leadership for QI

A large number of instruments measured aspects of organizational climate for QI or change, some developed specifically for primary care (*e*.*g*., Bobiak 2003 [102] and Ohman-Strickland 2007 [103] are measures of change capacity). Parker 1999 [58] and Shortell 2000 [57] were the most comprehensive instruments developed specifically for QI (rather than change in general) and include scales measuring leadership for QI. The content of these instruments reflects the structure and processes of large organisations; however, no equivalent instruments were found for primary care. Instruments described by developers as measuring culture (*e*.*g*., Kralewski, 2005 [106]), organizational learning (*e*.*g*., Marchionni, 2008 [107]) and readiness for change (*e*.*g*., Helfrich, 2009 [95]) often had considerable overlap in content with instruments measuring QI climate (*e*.*g*., Meurer, 2002 [108]). Culture and climate are related constructs [85], which was reflected in similar instrument content. Instruments explicitly identified by developers as measuring culture are identified as such in Additional file 4: Table S4 (indicated by E rather than X). The wording of items in instruments measuring culture focused on values, rather than policies and practices. However, most content from instruments measuring culture was categorised under generic climate because the dimensions were the same (*e*.*g*., Taveira 2003 [109] and Zeitz 1997 [110]).

##### Measures of organizational readiness for change

We categorised instrument content as measuring organizational readiness for change when items or the item context referred to an imminent change (*e*.*g*., Gustafson 2003 [42], Helfrich 2009 [95], and Øvretveit 2004 [96]). Content designed to elicit views on change in general was included under other categories of organizational context (*e*.*g*., Lehman, 2002 [111] and Levesque, 2001 [112]). Instruments that were explicitly identified by developers as measuring readiness for change are identified as such in Additional file 4: Table S4 (indicated by E rather than X).

#### 3) Individual level factors

Additional file 4: Table S6 reports the final taxonomy and categorisation of instrument content for the individual level factors domain (boxes numbered ‘3’ in InQuIRe framework).

##### Description of the individual level factors domain

Instruments were included in this domain if they measured individual: capability and empowerment for QI and change; commitment, goals, and motivation; and readiness for change. Our final taxonomy reflects frameworks for understanding individual level factors thought to influence behaviour change [113,114], and the results of our content analysis (key sources include [115-118]). We focused on individual capabilities and beliefs hypothesised to directly impact on collective capacity for CQI.

##### Measures of individual level factors

Within each of the three categories, we identified instruments that referred to CQI and others that referred more generally to QI and change. We focussed on CQI specific measures (*e*.*g*., Hill 2001 [119]; Coyle Shapiro 2003 [120]; Geboers 2001b [121]) or measures for which there were few organisation level equivalents. The latter included measures of commitment to change (*e*.*g*., Fedor 2006 [122], Herscovitch 2002a and 2002b [115]) and readiness to change (*e*.*g*., Armenakis 2007 [118], Holt 2007 [116]). We identified multiple measures of perceived CQI capability (*e*.*g*., Calomeni 1999 [123], Ogrinc 2004 [124], Solberg 1998 [56]) and knowledge ‘tests’ (*e*.*g*., Gould 2002 [125]). Overall, there were few comprehensive, theory-based measures of CQI-specific constructs. Good examples of theory-based measures were instruments measuring empowerment for QI (Irvine 1999 [126]) and motivation to use CQI methods (Lin 2000 [80]).

### Instrument characteristics, development and measurement properties

In stage four, we reviewed the development and measurement properties of 41 instruments with use or the potential for use in primary care. Additional file 5: Tables S7, S8, and S9 report the main characteristics of each instrument. Each table covers a separate content domain, and the order of instruments matches that used in Additional file 4: Tables S3, S4, S5, and S6. The purpose for which the instrument was first developed and the dimensions as described by the developers are summarised. The number of items, response scale, and modified versions of the instrument are reported, together with examples of use relevant to evaluation of CQI.

Additional file 4: Table S10 gives an overview of the development and testing of measurement properties for each instrument, indicating the extent of evidence reported in the main report(s) and any other studies in relevant contexts (as referenced in Additional file 6: Tables S11, S12, and S13). The development and testing of each instrument is described in Additional file 6: Tables S11, S12, and S13.

Although most papers provided some description of the instrument content and theoretical basis, constructs were rarely defined explicitly and reference to theory was scant. Reports of instruments arising from the healthcare and psychological literatures were notably different in this respect, the latter tending to provide comprehensive operational definitions that reflected related research and theory (*e*.*g*. Armenakis, 2007 [118] and Spreitzer 1995 [117]). Formal assessments of content validity (*e*.*g*., using an expert consensus process) were uncommon (examples of comprehensive assessments include Ohman-Strickland, 2007 [103], Kralewski, 2005 [106], and Holt, 2007 [116]). For most instruments, evidence of construct validity (*e*.*g*., through hypothesis testing, analysis of the instrument’s structure or both) was derived from one or two studies, and no evidence of construct validity was found for seven of the 41 instruments. Only one study used methods based on item response theory to assess construct validity and refine the instrument (Bobiak 2009 [102]).

Most studies report Cronbach’s alpha (a measure of internal consistency or the ‘relatedness’ between items) for the scale or, where relevant, subscales; however, it was common that this was done without checks to ensure that the scale was unidimensional (*e*.*g*., using factor analysis to ensure that items actually form a single scale and, hence, are expected to be related) [127,128]. Very few studies reported other assessments of reliability, thus providing limited evidence of the extent to which scores reflect a true measure of the construct rather than measurement error.

Consideration of conceptual and analytical issues associated with measuring collective constructs (*e*.*g*., organizational climate) was limited. Few authors discussed whether they intended to measure shared views (*i*.*e*., consensus is a pre-requisite for valid measurement of the construct), the diversity of views (*i*.*e*., the extent of variation within a group is of interest), or a simple average. Consequently, it was difficult to assess if items were appropriately worded to measure the intended construct and whether subsequent analyses were consistent with the way the construct was interpreted.

Very few studies reported the potential for floor and ceiling effects—which may influence both the instrument’s reliability and its ability to detect change in a construct [128]. None of the studies provided any guidance on what constitutes an important or meaningful change in scores on the instrument. Information about the acceptability of the instrument to potential respondents and feasibility of measurement was provided for less than one quarter of instruments, with most basing assessments on response rate only. Reporting of missing items, assessment of whether items were missing at random or due to other factors, and the potential for response bias [128,129] was dealt with in only a handful of studies.

## Discussion

This review aimed to provide guidance for researchers seeking to measure factors thought to modify the effect of CQI in primary care settings. These factors include contextual factors at organizational and individual level, and the implementation and use of CQI. We found many potentially relevant instruments—some reflecting pragmatic attempts to measure these factors and others the product of a systematic and theory-based instrument development process. Distinguishing the two was difficult, and the large number of factors measured and highly varied labelling and definition makes the process of selecting appropriate instruments complex. Limited evidence of the measurement properties of most instruments and inconsistent findings across studies increases the complexity. We discuss these findings in more depth, focussing first on the three content domains covered by the review. We then discuss overarching considerations for researchers seeking to measure these factors and explore opportunities to strengthen the measurement methods on which CQI evaluations depend.

### Measurement of CQI implementation and use

There were few self-report instruments designed to measure the implementation and use of CQI (fidelity) and most of those identified had undergone limited assessment of their measurement properties. Such measures provide important explanatory data about whether outcomes can be attributed to the intervention and the extent to which individual intervention components contribute to effects [19-21]. They can also provide guidance for implementing CQI in practice. However, there are challenges with developing these instruments.

First, there is limited consensus in the literature on what defines a CQI intervention and its components, and large variability in the content of CQI interventions across studies [86]. In part, this is attributed to the evolution and local adaptation of CQI interventions. However, it also reflects differences in how CQI interventions are conceptualised [130]. For this review, we adopted a definition that encompasses a set of QI methods relevant to teams in any setting, irrespective of size and structure. If we are to develop measures of CQI and accumulate evidence on its effectiveness, then it is essential to agree on the components that comprise the ‘core’ of CQI interventions and to further recent attempts to develop operational definitions of these components [86].

Second, measures of the use of CQI interventions need to address non-content related dimensions of intervention fidelity. Frameworks for specifying and defining these dimensions exist for health behaviour change interventions. The dimensions covered by these frameworks include intervention intensity (*e*.*g*., duration, frequency), quality of delivery, and adherence to protocols [131,132]. In public health, frameworks such as RE-AIM include assessment of intervention reach (target population participation), implementation (quality and consistency of intervention delivery), and maintenance (use of intervention in routine practice) [133]. These frameworks are broadly relevant, but most assume interventions are ‘delivered’ to a ‘recipient’ by an ‘interventionist’ [132], which does not reflect how CQI interventions are used. For QI interventions, assessing ‘intensity,’ ‘dose,’ ‘depth,’ and ‘breadth’ has been recommended [134,135]. Improved measurement will require agreement and definition of the dimensions of fidelity most relevant to CQI.

Finally, the validity of self-report instruments measuring the fidelity of use of CQI methods needs to be assessed against a criterion, or gold standard, measure of actual behaviour [131]. Measures that involve direct observation of CQI teams and expert evaluation of CQI process are likely to be best for this purpose [131,136] and examples of their use exist [1]. In other contexts, behavioural observation scales (typically, scoring of frequency of behaviours on a Likert scale) and behaviourally anchored rating scales (rating of behaviour based on descriptions of desirable and undesirable behaviours) have been used to facilitate rating of teamwork behaviours by observers [137]. While these methods are not feasible for large-scale evaluation, direct observation of CQI teams could be used to inform development and assess the validity of self-report instruments.

### Measurement of organizational context

The wide range of potentially relevant instruments included in the review illustrates the scope of possible measures of organizational context (Additional file 4: Table S4). A positive development is the emergence of instruments for measuring context in small healthcare settings (*e*.*g*., [103,106]). Instruments developed for large organizational settings still dominate the literature. Some have content and item wording that reflects the structure or processes of large organisations; however, there are a number of instruments suitable for small healthcare settings and others that could be adapted. A good example is the primary care organizational assessment instrument [138] that was adapted from a well-established, theoretically-sound instrument developed for the intensive care unit [139]. Testing is required to ensure the suitability of instruments in new settings. For example, evidence is accumulating that the widely used competing values instrument for measuring organizational culture may not be suitable for discriminating culture types in primary care [140-144].

### Measurement of individual level factors

A number of CQI-specific instruments measured individual level factors; among them were several instruments that had a strong theoretical basis and prior use in CQI evaluations (see Additional file 4: Table S6). The relationship between individual level factors and the outcomes of a group level process like CQI is complex [67,145]. For example, although individual capability and motivation to participate in CQI may influence outcomes, it is unclear how these individual level factors translate to overall CQI team capability and motivation, a factor that may be more likely to predict the performance of the CQI team. Team members often combine diverse skills and knowledge; conceptually, this collective capability is not equivalent to the average of individual members’ capability. This underscores the importance of ensuring item wording and methods of analysis reflect the conceptualisation of the construct and, in turn, the level at which inferences are to be made [67,146]. Particular care may be required when using and interpreting individual level measures in relation to a collective process such as CQI.

### Key considerations for researchers

The review findings highlighted two areas in particular that need careful consideration: the implications of using existing instruments versus developing new ones; and ensuring constructs and associated measures are clearly specified.

#### Implications of using existing instruments versus developing new ones

Given recent emphasis on measuring context in evaluations of quality improvement, and the concomitant proliferation of new measurement instruments, the value of using existing instruments needs to be emphasised. Streiner and Norman caution against researchers’ tendency to ‘dismiss existing scales too lightly, and embark on the development of a new instrument with an unjustifiably optimistic and naïve expectation that they can do better’ [128]. By using existing instruments, researchers capitalise on prior theoretical work and testing that ensures an instrument is measuring the intended construct and is able to detect changes in the construct or differences between groups. The use of existing instruments can therefore strengthen the findings of individual studies and help accumulate evidence about the instrument itself. The latter is important because many instruments in this review have very little evidence supporting their measurement properties, and less still in contexts relevant to evaluation of CQI in primary care. Investing in new instruments rather than testing existing scales fragments efforts to develop a suite of well-validated measures that could potentially be used as a core set of measures for QI evaluation [16].

Using existing instruments also increases the potential for new studies to contribute to accumulated knowledge. Many of the ‘theories’ about the influence of specific contextual factors on the use and outcomes of CQI come from the findings of one or two studies, or have not been tested [12,134]. In organizational psychology, meta-analysis is widely used to investigate the association between contextual factors and outcomes, largely with the aim of testing theories using data from multiple studies. Using existing scales with good evidence of validity and reliability would enhance our ability to synthesise the findings across studies to investigate theories about the relationship between context, use of CQI and outcomes.

An initiative that may provide a model for addressing these issues is the Patient-Reported Outcomes Measurement Information System (PROMIS) [147]. PROMIS is a coordinated effort to improve the measurement of patient reported outcomes through: the development of a framework for defining essential measurement domains; systematically identifying and mapping existing measures to the framework; and using items derived from these measures to develop a bank of highly reliable, valid scales. An equivalent resource in quality improvement could lead to substantive gains.

#### Ensuring constructs and associated measures are clearly specified

Our attempts to identify relevant instruments underscored the importance of clarity and consistency in the way factors are defined and measured. Consistent labelling of instruments measuring similar constructs aids the indexing of studies, increasing the likelihood that researchers and decision makers will be able to retrieve and compare findings of related studies. Using well-established construct definitions as the basis for instrument development helps ensure that instruments aiming to measure the same construct will have conceptually similar content. This is particularly important for comparison across studies and synthesis because it reduces the chance that readers will erroneously compare findings across studies that appear to be measuring the same construct but are in fact measuring something quite different. Such comparisons have the potential to dilute findings when comparing or pooling across multiple studies and, in areas where there is little comparable evidence, may lead to false associations between a construct and the outcomes of QI.

To address these issues, future research should build on existing theoretical work and place greater emphasis on providing clear concept labels and definitions that reference or extend those in existing research. This is an important but substantial task because of the large body of theoretical and empirical research in psychology and social sciences underpinning many constructs. However, developing a ‘common language’ for contextual factors and intervention components may reveal that there is much less heterogeneity across studies than the literature suggests, and hence, much more potential to synthesise existing research [148,149].

In developing the taxonomy presented in this review, we aimed to reflect prevailing labelling and conceptualisations of factors that may affect the success of CQI. The starting point for the structure and content of the taxonomy was the initial version of our InQuIRe framework. Our analysis led to elaboration of many of the constructs in our initial framework, some refinement to the categorisation of constructs within domains, but no changes to the overall structure of domains. The resulting taxonomy (Additional file 4: Tables S3, S4, S5, and S6) provides a guide to the factors that could be included in evaluations of CQI in primary care. The refinements and construct definitions derived from our measurement review (reported in this paper and the companion paper on team measures) will be incorporated in the final version of the InQuIRe framework (to be reported separately).

### Strengths and limitations

To our knowledge, this is the first attempt to collate and categorise the wide range of instruments relevant to measuring factors thought to influence the success of CQI. We used a broad systematic search and an inclusive approach when screening studies for potentially relevant instruments; however, we cannot rule out that we may have missed some instruments. We limited the review to instruments with information about their development and measurement properties reported in peer-review publications (*i*.*e*., not books, theses, or proprietary instruments), reasoning that these instruments were readily available to researchers.

The taxonomy we developed draws upon the wide range of instruments identified and allows comparison of instruments using a ‘common language.’ The process of developing and applying the taxonomy revealed the complexity of comparing existing instruments and the consequent value of taxonomies for helping QI researchers make sense of heterogeneity. Because this is the first application of the taxonomy and categorisation of instruments, refinement of the taxonomy is likely. A single author developed the taxonomy and categorised instruments with input from the other authors. Given the subjectivity inherent in this type of analysis, alternative categorisations of instruments are possible.

Although not a limitation of the review, there is comparatively little research on the measurement of organizational factors in healthcare. This increases the complexity of selecting and reviewing instruments because current evidence on the measurement properties of relevant instruments is limited and heterogeneous. Rising interest in this area means the number of studies will increase, however the heterogeneity is likely to remain because of the diversity of study designs that contribute evidence of an instrument’s measurement properties. Interpreting and synthesising this evidence is complex. Guidance on appraising the methods used in these studies, interpreting the findings, and methods for synthesising findings across studies would aid both the selection and the systematic review of instruments.

## Conclusions

Investigating the factors thought to modify the effects of CQI poses practical and methodological challenges for researchers, among the most complex of which relate to measurement. In this review, we aimed to provide guidance to support decisions around the selection of instruments suitable for measuring potential modifying factors. For researchers and those evaluating CQI in practice, this guidance should lessen the burden of locating relevant measures and may enhance the contribution their research makes by increasing the quality of measurement and the potential to synthesise findings across studies. Methodological guidance on measurement underpins our ability to generate better evidence to support policy and practice. While reviews such as this one can make a contribution, identification of a core set of measures for QI could ensure important factors are measured, improve the quality of measurement, and support the accumulation and synthesis of evidence [16]. Ultimately, a coordinated effort to improve measurement, akin to the Patient-Reported Outcomes Measurement Information System [147], may be required to produce the substantive gains in knowledge needed to inform policy and practice.

## Competing interests

Heather Buchan is a member of the Implementation Science Editorial Board. The authors have no other competing interests.

## Authors’ contributions

SB and SG conceived the study with input from HB. SB designed the review, conducted the searching, screening, data extraction and analysis. SG, HB, and MB provided input on the design, provided comment on the analysis and the presentation of results. SB drafted the manuscript and made subsequent revisions. All authors provided critical review of the manuscript. All authors read and approved the final manuscript.

## Supplementary Material

Additional file 1 Glossary of terms used in the review. Click here for file

Additional file 2 Search terms. Click here for file

Additional file 3 Tables reporting the content of instruments (Tables S3-S6) and overview of development and assessment of measurement properties (Table S10). Click here for file

Additional file 4 Tables reporting the content of instruments (Tables S3-S6) and overview of development and assessment of measurement properties (Table S10). Click here for file

Additional file 5 Tables summarising the characteristics of instruments included for review of measurement properties (Tables S7-S9). Click here for file

Additional file 6 Tables summarising the development and measurement properties of instruments included in Stage 4 of the review (Tables S11–S13). Click here for file
