# Supplementary material for: Measuring organizational and individual factors thought to influence the success of quality improvement in primary care: a systematic review of instruments
Source: Implement Sci. 2012 Dec 17;7:121. doi: 10.1186/1748-5908-7-121 (PMC3573896; doi:10.1186/1748-5908-7-121)
Supplement: Additional file 2 — Search terms. [file 1748-5908-7-121-S2.pdf]

Additional file 2: search terms[1]

Box 1: controlled vocabulary and free-text terms used to search MEDLINE

1. (questionnaire or questionnaires).ti,ab.
2. questionnaire/
3. (instrument or instruments or instrumentation).ti,ab.
4. tool?.ti.
5. (measuring or measures or measure or measurement).ti.
6. or/1-5
7. (improvement and quality).ti,ab.
8. (change and (organi#ations or organi#ational)).mp.
9. continuous improvement.ti,ab.
10. total quality management/
11. (change and practice).ti,ab.
12. quality of life.mp.
13. 7 not 12
14. 8 or 9 or 10 or 11 or 13
15. 6 and 14

[1] The final set of terms was decided following scoping searches to (i) test search terms for retrieval of relevant instruments and reviews of which we were already aware, and (ii) gauge the likely yield of references. These scoping searches resulted in high yields and identified three systematic reviews of instruments measuring two constructs in the conceptual framework (readiness for change [1-2] and organisational culture [3]). Collectively, these reviews included comprehensive searches of health and non-healthcare databases. We also identified a review of survey-based studies of quality management published in the business and management literature [4]. Given the high initial yield, we used these reviews, plus snowballing searches, as our primary sources for measures from the business and management literature. This enabled us to focus our database searches on MEDLINE, PsycINFO and HAPI using controlled vocabulary (thesaurus terms and subject headings) and free-text terms for quality improvement and practice change.

References

1. Holt DT, Armenakis AA, Harris SG, Feild HS: **Toward a comprehensive definition of readiness for change: a review of research and instrumentation.** In *Research in organizational change and development. Volume* 16. Edited by Pasmore WA, Woodman RW: Emerald Group Publishing Limited; 2007: 289-336
2. Weiner BJ, Amick H, Lee S-YD: **Conceptualization and measurement of organizational readiness for change: a review of the literature in health services research and other fields.** *Med Care Res Rev* 2008, **65:**379-436.
3. Mannion R, Davies H, Scott T, Jung T, Bower P, Whalley D, McNally R: **Measuring and assessing organisational culture in the NHS (OC1)**. National Co-ordinating Centre for National Institute for Health Research Service Delivery and Organisation Programme (NCCSDO) 2008.
4. Sila I, Ebrahimpour M: **An investigation of the total quality management survey based research published between 1989 and 2000: A literature review.** *International Journal of Quality & Reliability Management* 2002, **19:**902-970.
