# Supplementary material for: Measuring organizational and individual factors thought to influence the success of quality improvement in primary care: a systematic review of instruments
Source: Implement Sci. 2012 Dec 17;7:121. doi: 10.1186/1748-5908-7-121 (PMC3573896; doi:10.1186/1748-5908-7-121)
Supplement: Additional file 3 — Tables reporting the content of instruments (Tables S3-S6) and overview of development and assessment of measurement properties (Table S10). [file 1748-5908-7-121-S3.pdf]

Additional file 3: Definition of measurement properties (Table 1) and appraisal criteria (Table 2)

Table 1: Definition of measurement properties and description of assessments

Property	Definition and description of assessments[1]
Validity	"The degree to which accumulated evidence and theory support specific interpretation of test scores entailed by proposed uses of a test." ([1] p184).[2]
Content validity	The extent to which the content of the instrument clearly and comprehensively reflects the construct it is intended to measure.<br>Assessment: typically expert or independent assessment of the instrument against a detailed definition of the construct to determine (i) appropriateness of content for intended purpose, (ii) extent to which individual items are relevant to the content domain, and (iii) extent to which the entire set of items comprehensively represents all dimensions of the construct [1-3].
Construct validity – hypothesis testing	The extent to which the instrument measures the construct intended based on accumulated evidence from testing hypotheses about (i) the association between scores on the instrument and theoretically related variables, and (ii) the difference in scores between groups expected to differ on the construct.<br>Hypothesis testing should assess whether scores on the instrument (i) 'converge' with related variables -including other measures of the same construct, (ii) 'discriminate' between groups expected to differ on the construct, (iii) predict relevant outcomes, and (iv) concur with scores on a criterion – or gold standard – measure of the construct (e.g. a long form of instrument). Hypotheses should be pre-specified and include the expected direction (positive or negative) and magnitude (absolute or relative) of correlation or difference between groups [1, 3].
Construct validity - structure	The extent to which items on the proposed scale (or subscales) relate to each other in a way that is consistent with the theoretically predicted dimensions of the construct [1].<br>Assessment: confirmatory factor analysis (CFA) to test a priori hypotheses about the relationship between items. In the absence of an a priori hypothesis about the dimensions of a construct, exploratory factor analysis of the instrument's structure may be used to (i) identify dimensions, (ii) assess the unidimensionality of scales or subscales to confirm that items can be summed and before assessing internal consistency, and (iii) assess whether there are redundant items or items that relate poorly to the construct (i.e. during instrument development). Approaches based on item response theory may also be used.
Reliability[3]	The extent to which an instrument yields scores attributable to the 'true' score and not measurement error. When the 'true' score is unchanged, reliable instruments should produce reproducible scores in a range of conditions.<br>Assessments: (i) tests of whether the instrument yields consistent scores on items from the same scale (internal consistency – "the interrelatedness among the items" [4], p742), (ii) stable scores over time (test-retest reliability), and (iii) consistent scores with different raters (inter-rater reliability).

Property	Definition and description of assessments[1]
Other assessments	
Acceptability	Assessments of whether the instrument is acceptable to respondents.<br>Assessments: direct assessment of respondent views on burden and complexity of the questionnaire, or indirect assessment involving (i) time to complete (instructions and response time), (ii) response rate, and (iii) missing responses to items and whether there is potential for response bias.
Feasibility	Assessments of the feasibility of administering and scoring the instrument.<br>Assessments: time to administer, time to score or process data.
Level of analysis	The extent to which the content of the instrument, and the analysis and interpretation of resulting data, is consistent with the level at which the construct is defined.<br>Assessments: Clear statement of (i) the level at which the construct is conceptualised (e.g. group, organisation), and (ii) how the construct is conceptualised (e.g. as a 'shared' property which is meaningful only if there is within group consensus; as a property in which the extent of variation within groups is of interest). Instrument content, data analysis and interpretation is consistent with the conceptualisation of the construct.
Responsiveness	The extent to which an instrument detects changes over time, where changes are actually present. Responsiveness is a form of validity relating to change scores [3, 5].<br>Assessment: analogous to those used to assess construct validity [3, 5], focussing on change scores rather than cross sectional scores.
Interpretability	The extent to the instrument captures the full range of responses relevant to assessing the construct and can detect *important* changes or differences between groups.<br>Assessment: reporting of distribution of scores and potential for ceiling and floor effects; formal assessment of the smallest difference in scores considered important or meaningful (i.e. minimal important change or difference).[3, 5]
Generalisability	Reporting of information to enable assessment of the extent to which the findings about the instrument's measurement properties can be generalised.<br>Assessment: sample frame and selection described; response rate and analytical sample reported.

1. Multiple sources were used to identify, define and describe each property [1-2, 4-10]. Where the definition or description closely matches a particular source, the reference is provided in the text.
2. In this review, 'Test' is considered a synonym for 'instrument' or 'scale'.
3. The definition and description of categories of reliability is based on classical test theory (CTT). Similar concepts exist for generalisability theory (GT) and item response theory (IRT), however different techniques are used to assess measurement error [1]. Definition and description was limited to CTT approaches because they dominated the literature reviewed in this paper.

Table 2: Criteria for appraising methods used to evaluate an instrument's measurement properties

Summary level	Review authors' judgment	Description	Criteria (developed from source references)	Source
General		Complete if data not reported under specific domain.		
Were missing items adequately addressed	% missing items described		YES: investigators reported either: i) average number of missing items/instrument or ii) % missing responses per item<br>NO: not described	COSMIN p22 [11]
	Handling of missing items described		YES: the investigators provided a clear description of how missing items were handled<br>NO: not described	COSMIN p22 [11]
	Sample size adequate	Refers to final sample size (excluding non-responders, drop outs, missing values)	Complete for assessments below as indicated.	COSMIN p22 [11]
Was the study free of important flaws	Free of important flaws in design or methods of the study	Flaws that might lead to biased results or conclusions e.g. exclusion of respondents with incomplete data	YES: study appears to be free of flaws that could put it at risk of bias<br>NO: study has important flaws in design or methods that might lead to bias	COSMIN p22 [11]
Were level of analysis issues addressed			YES<br>NO [skip items]	
	Level of analysis clearly defined	Level of analysis is an important consideration for organisation-level and other collective measures. Construct definitions, scales and analysis of resulting data must be consistent with the intended level of measurement (e.g. individual, team, organisational level) to ensure construct validity. Hence, the intended level of the construct must be clear, and associated measurement and analysis consistent with this intent.	YES: explicit statement of the unit or level of analysis and clear definition of the construct at this level<br>NO: level and definition of construct not reported or ambiguous	Malhotra p423 [12]
	Scale consistently reflects level of analysis	The intended level of the construct should be clear from the wording of item context (i.e. introductory statement), items, and response scales. Ambiguity may compromise the construct validity of the measure.<br><br>For example, "I am confident I can perform the tasks required in this change" versus "I am confident that members of my practice can perform the tasks required in this change". The former reflects an individual's perception of their own capability; the latter reflects an individual's perception of others' capability.	YES: wording of item context, items or both clearly direct respondent to answer in relation to the intended construct level.<br>UNCLEAR: incomplete reporting of item context, items or both<br>NO: wording of item context, item stem or both refers to multiple levels or is ambiguous.	Malhotra p423 [12] Klein [13-14]

Summary level	Review authors' judgment	Description	Criteria (developed from source references)	Source
	Adequate design and statistical methods for analysing higher level constructs from individual level data	Items of the type given in the first example (above) may be analysed and interpreted in multiple ways, for example:<br>i) As an individual-level construct.<br>ii) As an organisational-level construct, in which the construct is only valid if consensus exists among organisational members (i.e. shared perceptions). A threshold of within-group agreement is set, below which it is not valid to aggregate scores (i.e. clusters with low within-group agreement would be excluded from analysis).<br>iii) As an organisational-level construct, in which the extent of perceptual agreement is of interest (i.e. measuring strength of perceptions - a conceptually distinct construct). In this case, the analysis investigates "the array, pattern, variability within a group". (Klein 2007).<br><br>Analyses may involve i) tests to assess the appropriateness of aggregating data or ii) use of modelling techniques that preclude the need to aggregate data (e.g. Hierarchical Linear Modelling (HLM)). The latter provides data that allows assessment of the variance accounted for by different levels.	YES: analyses are appropriate for the specified unit of analysis and intended interpretation of the construct<br>UNCLEAR: intended unit of analysis is unclear; analyses not reported or insufficiently reported to permit judgement<br>NO: analyses are inappropriate or analyse were not reported and it is highly unlikely that appropriate analyses were undertaken	Chan [15] Klein [13-14]
Was internal consistency assessed			YES<br>NO [skip items]	COSMIN p24 [11]
	Scale consists of effect indicators	If YES, complete internal consistency assessment. If NO, skip assessment.<br>Internal consistency applies to scales based on a reflective model, but is not relevant if the items "together form the construct" (COSMIN manual p24). To check, consider whether all items are expected to change when the construct changes.<br>Items are expected to be moderately correlated if they are all manifestations of the same underlying construct (i.e. based on a reflective model).	YES: scale i) consists of effect indicators, ii) is explicitly reported as based on a reflective model, or iii) the reviewer judges that all item scores would be expected to change if the construct changes.<br>UNCLEAR: not reported or not clear whether items are effect or causal indicators<br>NO: i) scale consists of causal indicators, ii) is explicitly reported as based on a formative model, or iii) the reviewer judges that all items scores would not be expected to change if the construct changes.	COSMIN p24 [11] Streiner p68-9 [5]
	% missing items described		YES: investigators reported either: i) average number of missing items/instrument or ii) % missing responses per item<br>NO: not described	COSMIN p24 [11]
	Handling of missing items described		YES: the investigators provided a clear description of how missing items were handled<br>NO: not described	COSMIN p24 [11]
	Sample size adequate for internal consistency analysis	Refers to final sample size (i.e. excludes non-responders, drop outs, missing values)<br>Reliability: sample size calculation for confidence intervals (CI) around Intra-class correlation coefficient (ICC)	YES: sample size calculation for CI around ICC<br>UNCLEAR: sample size not reported (record in notes)<br>NO: sample size not calculated; or highly unlikely that sample size calculated (record in notes)	COSMIN p24 [11]

Summary level	Review authors' judgment	Description	Criteria (developed from source references)	Source
	Unidimensionality of scale checked	Scale (or subscales) must be unidimensional for internal consistency to be interpretable.	YES: Factor analysis or item response theory (IRT) model applied to check dimensionality of scale (or subscales) and scale or subscales confirmed as unidimensional<br>UNCLEAR: analysis performed but incompletely reported or results unclear.<br>NO: unidimensionality not checked	COSMIN p24 [11]
	Sample size adequate for unidimensionality analysis	Recommendations for factor analysis: total sample ≥ 100 (although ≥ 50 allowed in some reviews) and the ratio of subjects to variables ranges from 4:1 to 10:1. Terwee criteria scores 7:1 ratio as 'YES'.	YES: For factor analysis, ≥ 100; subject to variable ratio: 4:1 to 10:1<br>UNCLEAR: sample size not reported (record in notes)<br>NO: sample size < 100; subject to variable ratio: < 4:1 (record in notes)	COSMIN p24 [11] Terwee 2007 [16]
	Internal consistency statistic calculated for each (sub)scale		YES: Internal consistency statistic calculated for each (sub)scale<br>UNCLEAR: not reported<br>NO: not done	COSMIN p24 [11]
	Important flaws in design or methods of the study		YES: study appears to be free of flaws that could put it at risk of bias<br>NO: study has important flaws in design or methods that might lead to bias	COSMIN p24 [11]
	Cronbach's alpha calculated	Classical test theory (CTT)	YES<br>UNCLEAR<br>NA: not applicable	COSMIN p24 [11]
	Cronbach's alpha or KR20 calculated (dichotomous scores)	Applies to dichotomous scores only.	YES<br>UNCLEAR<br>NA: not applicable	COSMIN p24 [11]
	Goodness of fit statistic at global level calculated	Item response theory (IRT)	YES<br>UNCLEAR<br>NA: not applicable	COSMIN p24 [11]
Was reliability assessed (relative measures)		Answer for relative measures: test-retest (also, inter- and intra-rater reliability, but these are less likely to be used for QI context measures)	YES<br>NO [skip items]	

Summary level	Review authors' judgment	Description	Criteria (developed from source references)	Source
	% missing items described	Complete only when separate administration for reliability assessment.	YES: investigators reported either: i) average number of missing items/instrument or ii) % missing responses per item<br>NO: not described	COSMIN p26 [11]
	Handling of missing items described	Complete only when separate administration for reliability assessment.	YES: the investigators provided a clear description of how missing items were handled<br>NO: not described	COSMIN p26 [11]
	Sample size adequate for analysis	Refers to final sample size (excluding non-responders, drop outs, missing values).	YES: sample size calculation for CI around ICC<br>UNCLEAR: sample size not reported (record in notes)<br>NO: sample size not calculated; or highly unlikely that sample size calculated (record in notes)	COSMIN p26 [11]
	At least two measures available		YES<br>NO: single administration or not reported	COSMIN p26 [11]
	Independent administrations for two measures	i.e. first administration should not influence second, for example if respondents were aware of score on first test then the administrations were not independent.	YES<br>UNCLEAR: not reported<br>NO: administration not independent<br>NA: single administration only	COSMIN p26 [11]
	Time interval between administrations stated		YES<br>NO: not reported<br>NA: single administration only	COSMIN p26 [11]
	Construct not expected to change during this interval	i.e. no exposure to an intervention or other factor that might alter the construct in interim	YES: construct not expected to change<br>UNCLEAR: not reported and can't assess<br>NO: construct likely to change<br>NA: single administration only	COSMIN p26 [11]
	Time interval appropriate	i.e. short enough that no change to construct expected, long enough to prevent recall of item response	YES<br>UNCLEAR:not reported and can't assess<br>NO: too short or too long<br>NA: single administration only	COSMIN p26 [11]
	Equivalent test conditions for both administrations	e.g. type of administration, environment, instructions	YES<br>UNCLEAR: administration conditions not reported<br>NO: important difference in administration conditions<br>NA: single administration only	COSMIN p26 [11]
	Important flaws in design or methods of the study		YES: study appears to be free of flaws that could put it at risk of bias<br>NO: study has important flaws in design or methods that might lead to bias	COSMIN p26 [11]
	Intraclass correlation coefficient (ICC) calculated	Continuous scores only<br>Pearsons and Spearman's correlation coefficient may be reported, but are "considered inadequate because they do not account for systematic error." (COSMIN manual p27)	YES<br>UNCLEAR: not reported, but relevant<br>NO<br>NA: not applicable	COSMIN p26 [11]

Summary level	Review authors' judgment	Description	Criteria (developed from source references)	Source
	Kappa calculated	Dichotomous, nominal or ordinal scores only	YES<br>UNCLEAR: not reported, but relevant<br>NO<br>NA: not applicable	COSMIN p26 [11]
	Weighted Kappa calculated	Ordinal scores only	YES<br>UNCLEAR: not reported, but relevant<br>NO<br>NA: not applicable	COSMIN p26 [11]
	Weighting scheme described	e.g. linear, quadratic "Proportion agreement is considered not adequate" as "it does not correct for chance agreement" (COSMIN manual p27)	YES<br>UNCLEAR: not reported, but relevant<br>NO<br>NA: not applicable	COSMIN p26 [11]
Was measurement error assessed			YES<br>NO [skip items]	COSMIN p28 [11]
	% missing items described	complete only when separate administration for reliability assessment	YES: investigators reported either: i) average number of missing items/instrument or ii) % missing responses per item<br>NO: not described	COSMIN p28 [11]
	Handling of missing items described	complete only when separate administration for reliability assessment	YES: the investigators provided a clear description of how missing items were handled<br>NO: not described	COSMIN p28 [11]
	Sample size adequate for analysis	Refers to final sample size (excluding non-responders, drop outs, missing values)	YES: sample size calculation for CI around ICC<br>UNCLEAR: sample size not reported (record in notes)<br>NO: sample size not calculated; or highly unlikely that sample size calculated (record in notes)	COSMIN p28 [11]
	At least two measures available		YES<br>NO: not reported or single administration	COSMIN p28 [11]
	Independent administrations for two measures	i.e. first administration should not influence second, for example if respondents were aware of score on first test then the administrations were not independent.	YES<br>UNCLEAR: not reported<br>NO: not done or administration not independent<br>NA: single administration only	COSMIN p28 [11]
	Time interval between administrations stated		YES<br>NO: not reported<br>NA: single administration only	COSMIN p28 [11]
	Construct not expected to change during this interval	i.e. no exposure to an intervention or other factor that might alter the construct in interim	YES: construct not expected to change<br>UNCLEAR: not reported and can't assess<br>NO: construct likely to change<br>NA: single administration only	COSMIN p28 [11]
	Time interval appropriate	i.e. short enough that no change to construct expected, long enough to prevent recall of item response	YES<br>UNCLEAR: not reported and can't assess<br>NO: too short or too long<br>NA: single administration only	COSMIN p28 [11]

Summary level	Review authors' judgment	Description	Criteria (developed from source references)	Source
	Equivalent test conditions for both administrations	e.g. type of administration, environment, instructions	YES<br>UNCLEAR: administration conditions not reported<br>NO: important difference in administration conditions<br>NA: single administration only	COSMIN p28 [11]
	Important flaws in design or methods of the study		YES: study appears to be free of flaws that could put it at risk of bias<br>NO: study has important flaws in design or methods that might lead to bias	COSMIN p28 [11]
	Standard error of measurement (SEM), smallest detectable difference (SDC) or limits of agreement (LoA) calculated	SEM is preferred. "requirement of two administrations … implies that the calculation of the SEM based on Cronbach's alpha is considered not appropriate" (p29)	YES<br>UNCLEAR<br>NO<br>NA: not applicable	COSMIN p28, 29 [11]
Was data presented regarding content validity			YES<br>NO [skip items]	
	Method of item generation was likely to optimise content validity		YES: Item generation involved i) deductive approach such as comprehensive review of literature relevant to construct and existing instruments OR, for immature constructs, ii) inductive approach such as using interviews with subject matter experts or observation of 'critical incidents'.<br>UNCLEAR: insufficient information to asses or not reported; existing instrument modified but modifications not reported<br>NO: items generated by investigators without careful definition of construct domain (i.e. through literature or inductive process)	Hinkin p969 [17]
	Items assessed for relevance to the construct to be measured	Face validity only - involves assessment of whether items appear to measure construct, by experts or target population	YES: any assessment of relevance of items to construct<br>UNCLEAR<br>NO	COSMIN p30 [11]
Was the content validity assessed	Instrument assessed for comprehensive coverage of construct	Should include:<br>- assessment that items comprehensively cover the content domain<br>- clear description of the construct domain<br>- clear description of the theory on which the construct is based	YES: construct and theoretical basis described, and items independently assessed for comprehensiveness and relevance to study population<br>UNCLEAR: insufficient information reported to assess all three aspects OR no report of content validity assessment<br>NO: content validity not assessed, or assessed and appears to be fully described, but assessment does not cover both comprehensiveness and relevance to study population	COSMIN p30 [11] Hinkin [17]

Summary level	Review authors' judgment	Description	Criteria (developed from source references)	Source
	Items assessed for compre-hensiveness	Usually assessment by expert panel. May involve quantitative assessment using a scale to rate relevance, comprehensiveness etc of items.	YES: items independently assessed for comprehensiveness<br>UNCLEAR: insufficient information reported to determine whether assessment performed or extent of assessment.<br>NO: items not independently assessed for comprehensiveness	COSMIN p30 [11]
	Clear description of construct domain		YES: construct domain clearly described or reference provided to another source providing a clear description.<br>NO: no description or insufficient information to describe the construct domain. General statements about related research without clearly defining the construct domain as the investigators intended to measure it.	COSMIN p30 [11]
	Clear description of the theory on which construct is based (theoretical foundation)		YES: theoretical basis clearly described or reference provided to another source providing a clear description<br>NO: no description or insufficient information to describe the theoretical basis. General statements about related research without clearly stating the theoretical basis as it applies to this measure.	COSMIN p30 [11]
Was structural validity assessed			YES<br>NO [skip items]	COSMIN p32 [11]
	Scale consists of effect indicators	If YES, complete structural validity assessment. If NO, skip assessment.<br>Structural validity applies to scales based on reflective model, but is not relevant if the items "together form the construct" (COSMIN manual p24). To check, consider whether all items are expected to change when the construct changes.	YES: scale i) consists of effect indicators, ii) is explicitly reported as based on a reflective model, or iii) the reviewer judges that all item scores would be expected to change if the construct changes.<br>UNCLEAR: not reported or not clear whether items are effect or causal indicators<br>NO: i) scale consists of causal indicators, ii) is explicitly reported as based on a formative model, or iii) the reviewer judges that all items scores would not be expected to change if the construct changes.	COSMIN p32 [11]
	% missing items described	Complete only when separate administration for structural validity assessment.	YES: investigators<br>- reported either: i) average number of missing items/instrument or ii) % missing responses per item	COSMIN p32 [11]
	Handling of missing items described	Complete only when separate administration for structural validity assessment.	YES: the investigators provided a clear description of how missing items were handled	COSMIN p32 [11]
	Sample size adequate for analysis of structure	Recommendations for factor analysis: total sample ≥ 100 (although ≥ 50 allowed in some reviews); subject to variable ratio ranges from 4:1 to 10:1. Terwee scores YES for 7:1 (see table 1, p39)	YES: For factor analysis, ≥ 100; subject to variable ratio: 4:1 to 10:1<br>NO: sample size < 100; subject to variable ratio: < 4:1 (record in notes)<br>Unclear: sample size not reported (record in notes)	COSMIN p32 [11]

Summary level	Review authors' judgment	Description	Criteria (developed from source references)	Source
	Important flaws in design or methods of the study			COSMIN p32 [11]
	Exploratory or confirmatory factor analysis performed	Factor analysis preferred statistical analysis to assess structural validity. Confirmatory factor analysis (CFA) preferred. Assessment is necessary to examine the stability of factor structure [17] and confirm that items load onto subscales as predicted (CFA). The "rationale for retention and deletion of items" should be "clearly linked both theoretically and empirically" (Hinkin 1998 p975). Further, "scales should not be derived post-hoc, based only on the results of factor analysis" (Hinkin 1998 p977)	YES: factor analysis (FA) performed to examine factor structure and confirm hypothesised item loading onto subscales<br>UNCLEAR: not reported<br>NO: highly unlikely FA performed	COSMIN p32 [11]<br>Hinkin 1998 [17]
	IRT test for determining the dimensionality of items performed	Complete for papers reporting item response theory (IRT) analyses.	YES<br>NO<br>UNCLEAR: not reported	COSMIN p32 [11]
Was construct validity assessed by hypothesis testing			YES<br>NO [skip items]	COSMIN p33 [11]
	% missing items described	Complete only when separate administration for hypothesis testing.	YES: investigators reported either: i) average number of missing items/instrument or ii) % missing responses per item	COSMIN p33 [11]
	Handling of missing items described	Complete only when separate administration	YES: the investigators provided a clear description of how missing items were handled	COSMIN p33 [11]
	Sample size adequate	Sample size calculation for expected correlations between measures or differences between groups.	YES: sample size calculated prior to sampling and appears adequate<br>NO: sample size inadequate<br>UNCLEAR: not reported	COSMIN p33 [11]
	A priori hypotheses formulated regarding correlations or mean differences	Hypotheses should be specified before data collection to prevent study being at high risk of bias.<br>Includes hypotheses about (i) correlations with scores on other instruments and (ii) mean differences in scores between groups.	YES: i) hypotheses stated AND ii) reported that hypotheses were specified prior to data collection<br>UNCLEAR: hypotheses stated, but unclear if specified prior to data collection<br>NO: no hypotheses stated or not stated prior to data collection.	COSMIN p33 [11]
	Expected direction of correlation or mean differences included in the hypotheses	Hypotheses should specify whether direction is expected to be positive or negative	As above, specifying direction of (i) correlation or (ii) mean difference in score between groups.	COSMIN p33 [11]
	Expected absolute or relative magnitude of correlations or mean differences included in the	Hypotheses should specify (i) the absolute magnitude of correlation with another measure, or (ii) the magnitude of correlation relative to the magnitude of correlation with a third measure.	As above, specifying absolute or relative magnitude of (i) correlation or (ii) mean difference in score between groups.	COSMIN p33 [11]

Summary level	Review authors' judgment	Description	Criteria (developed from source references)	Source
	hypotheses			
	Comparator instrument adequately described	For studies testing convergent validity	YES: sufficient description of comparator instrument provided to permit assessment of construct and content<br>NO: comparator instrument not described or described in insufficient detail<br>NA: no comparator instrument	COSMIN p33 [11]
	Measurement properties of comparator instrument were adequate	If the comparator instrument properties are inadequate or unknown, the performance of new instrument in relation to the comparator cannot be assessed.	YES: i) measurement properties of comparator instrument reported or available and ii) properties are adequate<br>UNCLEAR: properties not reported<br>NO: properties reported and not adequate	COSMIN p33 [11]
	Important flaws in design or methods of the study		YES: study appears to be free of flaws that could put it at risk of bias<br>NO: study has important flaws in design or methods that might lead to bias	COSMIN p33 [11]
	Adequate design and statistical methods for testing hypotheses	p values should be avoided. "Validity testing is about whether the direction and magnitude of correlation is similar to what could be expected based on the construct(s) that are being measured" and whether "differences [between groups] are as large as could be expected. (COSMIN manual p34)	YES: design and analysis focus on direction and magnitude of correlation or difference between groups.<br>UNCLEAR: insufficient information reported to assess<br>NO: reports tests of statistical significance only	COSMIN p33 [11]
Was data provided to assess interpretability			YES<br>NO [skip items]	COSMIN p43 [11]
	% missing items described	Complete only when separate administration for this assessment.	YES: the investigators provided a clear description of how missing items were handled	COSMIN p43 [11]
	Handling of missing items described	Sample size calculation for expected correlations	YES: sample size calculated prior to sampling and appears adequate<br>NO: sample size inadequate<br>UNCLEAR: not reported	COSMIN p43 [11]
	Sample size adequate	Refers to final sample size (i.e. excludes non-responders, drop outs, missing values)	YES: final sample appears adequate<br>NO: sample size not calculated; or highly unlikely that sample size calculated (record in notes)<br>Unclear: sample size not reported (record in notes)	COSMIN p43 [11]
	Distribution of the (total) scores in the sample described	Entire distribution should be shown (e.g. in histogram), in addition to mean and standard deviation (SD)	YES: mean, SD and entire distribution shown<br>NO: distribution not described or insufficient description	COSMIN p43 [11]

Summary level	Review authors' judgment	Description	Criteria (developed from source references)	Source
	% respondents with lowest possible score reported	Required to assess floor effects, and concomitant effects on reliability and responsiveness to change.<br>> 15% respondents achieving lowest score suggests floor effects.	YES: reported<br>NO: not reported	COSMIN p43 [11] Terwee p39 [16]
	% respondents with highest possible score reported	Required to assess ceiling effects, and concomitant effects on reliability and responsiveness to change.<br>> 15% respondents achieving highest score suggests ceiling effects.	YES: reported<br>NO: not reported	COSMIN p43 [11] Terwee p39 [16]
	Scores and change scores reported for relevant groups	e.g. normative groups, general population, groups with expected differences	YES: reported for relevant groups<br>NO: not reported	COSMIN p43 [11]
	Minimal important change (MIC) or minimal important difference (MID) determined		YES: determined and reported<br>UNCLEAR: not reported<br>NO: not determined	COSMIN p43 [11]
	Important flaws in design or methods of the study		YES: study appears to be free of flaws that could put it at risk of bias<br>NO: study has important flaws in design or methods that might lead to bias	COSMIN p43 [11]
Was information reported to enable assessment of generalisability			YES<br>NO [skip items]	COSMIN p45 [11]
	Sample in which the instrument was evaluated was adequately described.	e.g. Setting, respondent characteristics, countries, language.<br><br>(Each attribute of the sample is scored separately in the COSMIN criteria; however, the assessment is combined here because the relevance of individual attributes varies for organisational measures depending on the construct, level and purpose of measurement.)	YES: adequate description<br>NO: not reported or inadequate description	COSMIN p45 [11]
	Method used to select sample was adequately described	e.g. random versus convenience or purposive.	YES: adequate description<br>NO: not reported or inadequate description	COSMIN p45 [11]
	% of missing responses (response rate) acceptable	Response rate (RR) at first and, if applicable, second administration (e.g. at baseline and follow up). Response rate may indicate whether there is a risk of selection bias. Selection bias may occur at multiple stages: (1) when potential respondents are invited to participate, and either consent to do so or decline, (2) when the instrument is administered and potential respondents complete the instrument or do not complete the instrument, (3) at follow up administration, where respondents at the first completion do not complete the instrument at follow up. For collective measures, both the	YES: adequate RR<br>NO: inadequate RR<br>UNCLEAR: not reported	COSMIN p45 [11]

Summary level	Review authors' judgment	Description	Criteria (developed from source references)	Source
		group level (cluster, team, group, unit organisation) and overall RR are important.		

References

1. Joint Committee on Standards for Educational and Psychological Testing (U.S.), American Educational Research Association., American Psychological Association., Education. NCoMi: *Standards for educational and psychological testing.* Washington, DC: American Educational Research Association; 1999.
2. Di Iorio CK: *Measurement in health behavior: methods for research and education.* 1st edn. San Francisco: Jossey-Bass; 2005.
3. Mokkink L, Terwee C, Knol D, Stratford P, Alonso J, Patrick D, Bouter L, De Vet H: **The COSMIN checklist for evaluating the methodological quality of studies on measurement properties: A clarification of its content.** *BMC Medical Research Methodology* 2010, **10:**22.
4. Mokkink LB, Terwee CB, Patrick DL, Alonso J, Stratford PW, Knol DL, Bouter LM, de Vet HCW: **The COSMIN study reached international consensus on taxonomy, terminology, and definitions of measurement properties for health-related patient-reported outcomes.** *Journal of Clinical Epidemiology* 2010, **63:**737-745.
5. Streiner DL, Norman GR: *Health measurement scales: a practical guide to their development and use.* 3rd edn. Oxford ; New York: Oxford University Press; 2003.
6. Fitzpatrick R, Davey C, Buxton MJ, Jones DR: **Evaluating patient-based outcome measures for use in clinical trials.** *Health technology assessment (Winchester, England)* 1998, **2:**i-iv, 1-74.
7. Mannion R, Davies H, Scott T, Jung T, Bower P, Whalley D, McNally R: **Measuring and assessing organisational culture in the NHS (OC1)**. National Co-ordinating Centre for the National Institute for Health Research Service Delivery and Organisation Programme (NCCSDO) London 2008.
8. Mokkink LB, Terwee CB, Gibbons E, Stratford PW, Alonso J, Patrick DL, Knol DL, Bouter LM, De Vet HC: **Inter-rater agreement and reliability of the COSMIN (COnsensus-based Standards for the selection of health status Measurement Instruments) Checklist.** *BMC Med Res Methodol* 2010, **10:**82.
9. O'Leary-Kelly SW, J. Vokurka R: **The empirical assessment of construct validity.** *Journal of Operations Management* 1998, **16:**387-405.
10. Trochim WM: **The Research Methods Knowledge Base** [http://www.socialresearchmethods.net/kb/]
11. Mokkink LB, Terwee CB, Patrick DL, Alonso J, Stratford PW, Knol DL, Bouter LM, de Vet HC: **COSMIN checklist manual, version 6.0 (February 2010)**. Downloaded from: http://cosmin.nl 2010.
12. Malhotra MK, Grover V: **An assessment of survey research in POM: from constructs to theory.** *Journal of Operations Management* 1998, **16:**407-425.
13. Klein KJ, Conn AB, Smith DB, Sorra JS: **Is everyone in agreement? An exploration of within-group agreement in employee perceptions of the work environment.** *Journal of Applied Psychology* 2001, **Vol.86:**pp.
14. Klein KJ, Kozlowski SWJ: **From Micro to Meso: Critical Steps in Conceptualizing and Conducting Multilevel Research.** *Organizational Research Methods* 2000, **3:**211-236.
15. Chan D: **Functional relations among constructs in the same content domain at different levels of analysis: A typology of composition models.** *Journal of Applied Psychology* 1998, **83:**234-246.
16. Terwee CB, Bot SDM, de Boer MR, van der Windt D, Knol DL, Dekker J, Bouter LA, de Vet HCW: **Quality criteria were proposed for measurement properties of health status questionnaires.** *Journal of Clinical Epidemiology* 2007, **60:**34-42.
17. Hinkin T: **A brief tutorial on the development of measures for use in survey questionnaires.** *Organizational Research Methods* 1998, **1:**104-121.
