# Supplementary material for: Measuring organizational and individual factors thought to influence the success of quality improvement in primary care: a systematic review of instruments
Source: Implement Sci. 2012 Dec 17;7:121. doi: 10.1186/1748-5908-7-121 (PMC3573896; doi:10.1186/1748-5908-7-121)
Supplement: Additional file 4 — Tables reporting the content of instruments (Tables S3-S6) and overview of development and assessment of measurement properties (Table S10). [file 1748-5908-7-121-S4.pdf]

Table 3: Content of instruments measuring CQI implementation and use [1,2,3,4]

	Apekey, 2011a [84]	Engels, 2006 [150]	Geboers, 2001a [121]	Solberg, 1998a [56]	Solberg, 2008 (1) [151]	Alemi, 2001 [99] *	Alexander, 2007 [152] *	Barsness, 1993 [89]	Duckers, 2008 (1) [93]	Gilman, 1995 [153] *	Gustafson, 2003 (1) [42]	Helfrich, 2009 (1) [95]	Lemieux-Charles, 2002 [35]	Meurer, 2002 (1) [108]	Olsson, 2003a (1) [154] *	Øvretveit, 2004 (1) [96] *	Schouten, 2010 (1) [94]	Westphal, 1997 (1) [155] *	Wilkens, 2006 [156]
	primary care					other health care													
CQI implement process																			
Training in CQI principles, methods, tools				X															
content, satisfaction with																	X		
coverage, timing, duration																			
Facilitation of CQI process																			
capability and influence of facilitator									X		X	X				X	X		
type of support provided and effectiveness						X			X			X					X		X
commitment of facilitator			X			X			X		X	X					X		
Influencing roles																			
opinion leaders (support for CQI, QI or proposed change)												X				X	X		
champion					X							X							
Organisation-wide use of CQI methods																			
Continuous process improvement					X			X											
Structured problem solving, statistical methods and measurement					X			X											
Teams as major mechanism for improvement					X			X						X				X	
Empowerment to identify and act on opportunities for improvement					X			X						X					
Explicit focus on customers					X			X						X					
Use of CQI methods by QI teams																			
QI team formation, roles, meetings			X	X		X			X				X		X	X	X		
Setting overall aims			X	X		X							X		X	X	X		
Structured problem solving													X						
mapping existing process			X	X		X													
setting specific goals for process improvement		X	X	X		X									X	X	X		
selecting measures		X		X									X			X	X		
identifying sources of variation, root cause		X		X									X						
identifying and prioritising changes			X	X		X							X				X		
planning and testing changes on small scale		X	X			X							X				X		
Implementation & spread of changes				X		X													
Data collection and analysis during QI process																	X		
to characterise problem		X		X		X					X		X		X		X		
to identify sources & root cause of variation													X						
to identify potential changes/solutions				X							X						X		
to monitor the effect of changes		X		X		X					X		X			X	X		
Use of QI tools to collect, manage and analyse data			X																
tools used	X					X				X				X				X	
extent/frequency of use	X						X												
Use of QI tools to facilitate group process			X																
tools used	X									X			X					X	
extent/frequency of use	X						X							X					

[1] ‘a’ and ‘b’ are used to label instruments when multiple instruments are reported from a single paper (or two papers in the same year).

[2] (1) and (2) denote an instrument with content spanning more than one domain, (1) being the first entry and (2) the second entry.

[3] * Denotes instruments excluded from Stage 4 - review of measurement properties (hence, they do not appear in Table 10 or in Additional files 5 and 6).

[4] An ‘X’ may represent a single item, multiple items or an entire scale

Table 4: Content of instruments measuring organisational context in health care[1,2,3,4,5]

	Apekey, 2011b [1] *	Bobiak, 2009 [2]	Book, 2003 [3]	Geboers, 2001b (1)[4]	Kralewski, 2005 [5]	Leykum, 2011 [6]	Marsden, 2006 [7]	Ohman-Strickland, 2007 [8]	Olsson, 2003b [9]	Parker, 1999 [10]	Rushmer, 2007 [11]	Solberg, 2008 (2) [12]	Shortell, 1991 [13]	Stevenson, 2005 [14]	Sylvester, 2003 [15]	Duckers, 2008 (2) [16]	Estabrooks, 2009 [17] *	Fedor, 2006 [18] *	Freeman, 2003 [19] *	Gustafson, 2003 (2) [20]	Helfrich, 2009 (2) [21]	Ingersoll, 2000 [22] *	Kivimaki, 1997 [23] *	Lageson, 2006 [24]	Lameei, 2005 [25] *	Lehman, 2002 [26] *	Levesque, 2001 [27] *	Marchionni, 2008 [28] *	Meurer, 2002 (2) [29]	Nelson, 2002 [30] *	Olsson, 2003a (2) [31] *	Oudejans, 2011 [32] *	Øvretveit, 2004 (2) [33] *	Rondeau, 2002 [34] *	Rondeau, 2004 [35] *	Schouten, 2010 (2) [36]	Shortell, 2000 [37]	Siloaho, 2001 [38] *	West, 1998 [39] *	Westphal, 1997 (2) [40] *	Zammuto, 1991 [41]
	primary care															other health care																									
Capability																																									
Collective efficacy, skills, competency																																									
- for using CQI methods & tools																																									
- for change or improvement												X		X						X										X	X					X		X			
Capability development			X																																						
- history of using CQI methods or change					X			X			X	X		X																	X										
- training in CQI principles, methods, tools							X			X															X		X								X	X	X			X	
- training & development (not QI specific)						X					X	X		X	X				X	X	X			X		X			X	X	X	X		X			X				
- capacity for learning, knowledge sharing	X	X			X	X			X	X	X		X	X	X		X		X		X		X					X	X	X	X	X	X	X		X	X				
Commitment, goals, motivation																																									
Attitudinal indicators																																									
- beliefs about CQI			X	X																					X																
- beliefs about change, improvement, quality		X	X		X		X		X	X	X	X	X	X	X			X	X	X	X	X				X		X			X		X	X		X		X	X		
Behaviours indicating commitment																																									
- to CQI				X																																					
- to change, improvement or quality	X	X			X		X	X	X	X	X	X		X	X	X	X		X	X	X				X			X		X	X	X		X		X	X	X	X		
Factors motivating use of CQI/change															X				X							X							X								
Tension for change																				X											X		X								
Climate for QI or change																																									
Emphasis on process improvement									X	X		X												X	X		X	X		X					X	X	X		X		
Focus on customers' needs & expectations		X			X				X	X	X	X		X						X	X		X		X				X	X	X		X		X	X	X	X	X	X	
Staff involvement & participation	X			X	X	X	X	X	X	X	X		X	X	X	X		X	X	X	X		X		X			X	X		X	X	X	X	X	X	X	X	X		
Staff empowerment for QI				X				X	X	X	X	X				X					X							X	X		X				X		X	X			
Support for risk taking, experimentation, new ideas	X	X			X		X		X	X	X		X		X						X	X	X	X				X	X		X			X			X	X			
Data/information for monitoring & improvement					X				X	X	X	X	X				X		X		X									X	X	X		X	X		X		X	X	
- timeliness, accuracy, relevance										X	X												X						X	X							X				
- comparison with similar organisations	X									X					X													X						X	X		X				
QI alignment with organisational plans, goals									X	X		X				X				X									X						X	X	X				

Additional file 4: Brennan et al. Measuring organisational and individual factors thought to influence the success of quality improvement in primary care: a systematic review of instruments.

	Apekey, 2011b [1] *	Bobiak, 2009 [2]	Book, 2003 [3]	Geboers, 2001b (1)[4]	Kralewski, 2005 [5]	Leykum, 2011 [6]	Marsden, 2006 [7]	Ohman-Strickland, 2007 [8]	Olsson, 2003b [9]	Parker, 1999 [10]	Rushmer, 2007 [11]	Solberg, 2008 (2) [12]	Shortell, 1991 [13]	Stevenson, 2005 [14]	Sylvester, 2003 [15]	Duckers, 2008 (2) [16]	Estabrooks, 2009 [17] *	Fedor, 2006 [18] *	Freeman, 2003 [19] *	Gustafson, 2003 (2) [20]	Helfrich, 2009 (2) [21]	Ingersoll, 2000 [22] *	Kivimaki, 1997 [23] *	Lageson, 2006 [24]	Lameei, 2005 [25] *	Lehman, 2002 [26] *	Levesque, 2001 [27] *	Marchionni, 2008 [28] *	Meurer, 2002 (2) [29]	Nelson, 2002 [30] *	Olsson, 2003a (2) [31] *	Oudejans, 2011 [32] *	Øvretveit, 2004 (2) [33] *	Rondeau, 2002 [34] *	Rondeau, 2004 [35] *	Schouten, 2010 (2) [36]	Shortell, 2000 [37]	Siloaho, 2001 [38] *	West, 1998 [39] *	Westphal, 1997 (2) [40] *	Zammuto, 1991 [41]
QI integration with daily work																														X						X		X			
Recognition, rewards for QI or quality					X					X	X				X				X		X	X		X	X		X	X		X				X	X		X	X		X	
Generic climate																																									
Cooperation, collaboration, coordination		X			X	X	X	X			X	X	X	X	X	X	X		X		X	X	X			X		X		X		X						X			X
Communication, openness					X						X		X		X	X	X	X	X		X		X			X		X		X	X	X		X							X
Cohesion; shared goals, beliefs, values	X	X			X			X	X		X	X		X	X		X		X							X		X			X							X			X
Conflict		X						X					X		X																							X			
Role stress, overload, busyness, role clarity		X						X			X	X					X						X			X				X			X								
Job challenge, meaningfulness, autonomy					X								X		X								X							X											
Internal & external networks		X									X		X	X	X		X													X											
Leader behaviours that influence climate		X						X			X		X				X		X					X		X				X											X
Culture					X-E									X-E	X-E													X-E						X-E							X-E
QI specific dimensions		X			X						X			X	X		X				X							X						X							
Leadership for QI at all levels																																									
Sets clear goals & plans for quality, QI, change	X	X					X			X	X	X	X					X	X	X	X			X			X	X	X	X			X	X	X		X	X			
Commitment to QI (attitudes, behaviours)							X			X	X	X	X		X	X	X			X	X			X	X		X	X	X		X		X	X	X	X	X		X		
Enables involvement/empowerment								X		X			X										X	X	X		X	X		X	X						X				
Seeks & responds to staff suggestions, feedback, views							X			X			X		X			X		X	X		X	X			X	X	X		X						X				
Capability for QI and change							X			X			X							X	X			X	X				X	X	X						X				
Resources, supporting systems, structures					X																					X	X									X					
- for QI, improvement				X						X	X	X					X				X				X				X	X					X		X	X		X	
Organisational readiness for change																					X-E	X-E				X-E	X-E														
Commitment to imminent change																		X		X	X												X								
Efficacy for behaviours arising from change																				X																					
Resources allocated to a specific change																X		X		X	X										X		X								
Processes used to facilitate change																X		X		X	X										X		X								

[1] ‘a’ and ‘b’ are used to label instruments when multiple instruments are reported from a single paper (or two papers in the same year). [2] (1) and (2) denote an instrument with content spanning more than one domain, (1) being the first entry and (2) the second entry. [3] * Denotes instruments excluded from Stage 4 - review of measurement properties (hence, they do not appear in Table 10 or in Additional files 5 and 6). [4] An ‘X’ may represent a single item, multiple items or an entire scale [5] X-E denotes instruments labelled by developers as measures of ‘readiness’ or ‘culture’.

Table 5: Content of instruments measuring organisational context in non healthcare settings [1,2,3,4,5]

	Ahire, 1996 [42] *	Armenakis, 2007 (1) [43]	Bouckenooghe, 2009 [44] *	Dabhilkar, 2007 [45] *	Fisher, 2005 [46] *	Jansen, 2004 [47] *	Holt, 2007 (1) [48]	Taveira, 2003a [49] *	Taveira, 2003b [49]*	Zeitz, 1997a [50] *	Zeitz, 1997b [50] *
	non health care										
Capability											
Collective efficacy, skills, competency for CQI, change, improvement											
Capability development											
- history of using CQI methods or change	X										
- training in CQI principles, methods, tools								X			
- training & development (not QI specific)	X				X						
- capacity for learning, knowledge sharing				X	X						X
Commitment, goals, motivation											
Attitudinal indicators											
- beliefs about CQI					X						
- beliefs about change, improvement, quality					X				X	X	X
Behaviours indicating commitment											
- to CQI				X							
- to change, improvement or quality				X					X	X	X
Factors motivating use of CQI / change											
Tension for change											
Climate for QI or change											
Emphasis on process improvement				X	X			X		X	
Focus on customers' needs & expectations	X			X	X			X		X	
Staff involvement & participation	X		X	X	X			X		X	X
Staff empowerment for QI	X				X						X
Support for risk taking, experimentation, new ideas				X					X	X	X
Data & information for monitoring & improvement	X			X				X		X	
- timeliness, accuracy, relevance											
- comparison with similar organisations	X							X			
Alignment of QI with organisational plans & goals				X							
Integration of QI with daily work				X							
Recognition, rewards for QI or quality	X			X				X	X	X	X
Generic climate											
Cooperation, collaboration, coordination			X	X	X				X	X	
Communication, openness			X		X				X		X
Cohesion; shared goals, beliefs, values			X						X	X	X
Conflict					X						X
Role stress, overload, busyness, role clarity									X		
Job challenge, meaningfulness, autonomy									X		X
Internal & external networks				X							
Leader behaviours that influence climate			X						X		X
Culture									X-E		X-E
QI specific dimensions											X
Leadership for QI at all levels											
Sets clear goals & plans for quality, QI, change	X		X	X	X				X	X	
Commitment to QI (attitudes, behaviours)	X		X	X	X			X		X	
Enables involvement/empowerment			X		X						X
Seeks & responds to staff suggestions, feedback, views	X		X	X				X	X	X	X
Capability for QI and change			X							X	X
Resources, supporting systems, structures for QI				X							
Organisational readiness for change							X-E				
Commitment to imminent change		X	X			X	X				
Efficacy for behaviours arising from change		X									
Processes used to facilitate change			X								

[1] 'a' and 'b' are used to label instruments when multiple instruments are reported from a single paper (or two papers in the same year). [2] (1) and (2) denote an instrument with content spanning more than one domain, (1) being the first entry and (2) the second entry. [3] * Denotes instruments excluded from Stage 4 - review of measurement properties (hence, they do not appear in Table 10 or in Additional files 5 and 6). [4] An 'X' may represent a single item, multiple items or an entire scale. [5] X-E denotes instruments labelled by developers as measures of 'readiness' or 'culture'.

Table 6: Content of instruments measuring individual level factors [1,2,3,4]

	Calomeni, 1999 [51]	Geboers, 2001b (2) [4]	Lin, 2005 [52]	Solberg, 1998b [53]	Balding, 2005 [54] *	Cunningham, 2002 [55]	Fedor, 2006 [18] *	Francois, 2005 [56] *	Gould, 2002 [57] *	Herscovitch, 2002a [58]	Herscovitch, 2002b [58]	Hill, 2001 [59] *	Irvine, 1995 [60]	Irvine, 1999 [61]	Lehman, 2002 [26] *	Miller, 2000 [62] *	Ogrinc, 2004 [63] *	Oudejans, 2011 [32] *	Armenakis, 2007 (2) [43]	Campbell, 2006 [64] *	Coyle-Shapiro, 2003 [65]	Hanpachern, 1998 [66]	Holt, 2007 (2) [48]	Jansen, 2004 [47] *	Spreitzer, 1995 [67] *	Wanberg, 2000 [68] *
	primary care				other health care														non health care							
Capability & empowerment for QI & change																										
Existing knowledge in CQI specific domains																										
- knowledge tests									X																	
- perceived knowledge				X				X																		
- prior training & participation				X																						
Efficacy, perceived competency, perceived skills																										
- beliefs about capability to perform CQI activities	X	X							X					X			X									
- beliefs about capability to contribute to change or improvement	X								X					X			X	X		X					X	
Empowerment, agency, self-determination, autonomy					X									X											X	
- self determination or autonomy to initiate improvement														X				X							X	
- perceived ability to influence quality, QI outcomes, change					X									X	X			X							X	
Commitment, goals, motivation			X																							
Attitudinal measures																										
- beliefs about need for change, improvement (discrepancy)				X		X	X		X	X		X								X	X		X	X		X
- agreement with the principles & methods of CQI		X		X	X				X			X														
- beliefs about consequences & outcomes of CQI, QI or change	X	X	X	X	X			X	X			X				X				X	X					
- value placed on expected outcomes of QI, change	X		X							X										X			X	X		X
- perceived responsibility for & pride in quality, QI	X				X			X								X		X			X					
- specific factors that motivate participation	X	X						X		X		X														
Behaviours & intentions indicating commitment																										
- intention to participate in future CQI activities				X																						
- intention to participate in or support change, improvement						X	X	X			X											X				
- adopting behaviours consistent with CQI													X			X										
- adopting behaviours that support change, improvement		X				X	X				X		X		X			X		X	X					
Individual readiness for change						X-E													X-E			X-E	X-E			
Commitment to specific, imminent change							X			X	X											X	X	X		X
- attitudes indicating commitment										X									X				X	X		X
- behaviours or intentions indicating support for change							X				X											X				
Efficacy for change implementation process & behaviours arising																			X				X			

[1] ‘a’ and ‘b’ are used to label instruments when multiple instruments are reported from a single paper (or two papers in the same year).

[2] (1) and (2) denote an instrument with content spanning more than one domain, (1) being the first entry and (2) the second entry.

[3] * Denotes instruments excluded from Stage 4 - review of measurement properties (hence, they do not appear in Table 10 or in Additional files 5 and 6).

[4] An ‘X’ may represent a single item, multiple items or an entire scale. X-E denotes instruments labelled by developers as measures of ‘readiness’

Table 10: Overview of instrument development and assessments of measurement properties reported in included studies [1, 2]

	Apekey, 2011a [1]	Engels, 2006 [69]	Geboers, 2001a [4]	Solberg, 1998a [53]	Solberg, 2008 [12]	Barsness , 1993 [70]	Lemieux-Charles, 2002 [71]	Schouten, 2010 [36]	Wilkens, 2006 [72]	Bobiak, 2009 [2]	Book, 2003 [3]	Geboers, 2001b [4]	Kralewski, 2005 [5]	Leykum, 2011 [6]	Marsden, 2006 [7]	Ohman-Strickland, 2007 [8]	Olsson, 2003 [9]	Parker, 1999 [10]	Rushmer, 2007 [11]	Shortell, 1991 [13]	Stevenson, 2005 [14]	Sylvester, 2003 [15]	Duckers, 2008 [16]	Gustafson, 2003 [20]	Helfrich, 2009 [21]	Meurer, 2002 [29]	Lageson, 2006 [24]	Shortell, 2000 [37]	Zammuto, 1991 [41]	Calomeni, 1999 [51]	Lin, 2005 [52]	Solberg, 1998b [53]	Cunningham, 2002 [55]	Herscovitch, 2002a [58]	Herscovitch, 2002b [58]	Irvine, 1995 [60]	Irvine, 1999 [61]	Armenakis, 2007 [43]	Coyle-Shapiro, 2003 [65]	Hanpachern, 1998 [66]	Holt, 2007 [48]
	CQI implementation and use									Organisational context																				Individual level factors											
	Primary care					Other health care				Primary care													Other health care							Primary care			Other health care					Non health care			
Development and content validity																																									
- clear description of content domain	✓	✓	✓	✓	✓	✓	✓	✓✓	✓	✓	X	✓	✓✓	✓✓	✓	✓	X	✓	✓	✓✓	✓	✓✓	✓	✓✓	✓	✓	✓	✓	✓✓	✓	✓✓	✓	✓	✓✓	✓✓	✓✓	✓✓	✓✓	✓	X	✓✓
- clear description of theoretical basis	X	✓	✓	X	✓	✓	✓	✓✓	X	✓	✓	X	✓✓	✓	✓	✓	✓	X	✓	✓✓	✓	✓	✓✓	✓✓	✓	✓	✓	✓	✓✓	X	✓✓	X	✓✓	✓✓	✓✓	✓✓	✓✓	✓✓	✓	X	✓✓
- item development process reported	X	X	X	X	✓	X	X	✓	X	✓	✓	X	✓✓	✓	X	✓✓	X	X	✓	✓	✓	✓	✓	✓✓	✓	✓	X	X	?	X	X	X	X	X	X	✓✓	✓✓	✓✓	X	X	✓✓
- formal assessment of content validity	X	X	X	X	X	X	X	✓	X	X	X	X	✓✓	X	X	✓✓	X	?	✓✓	✓	X	X	✓	✓	X	✓✓	X	X	?	X	X	X	X	X	X	✓	✓	✓	X	X	✓✓
Construct validity																																									
Hypothesis testing																																									
- hypotheses formulated a priori	✓	✓	NA	✓	✓	✓	✓	NA	X	?	?	NA	✓	✓	✓	✓✓	NA	✓	NA	✓✓	NA	NA	✓✓	✓	✓	✓	NA	✓✓	✓✓	NA	✓	✓	✓	✓✓	✓✓	✓✓	✓✓	✓	✓	?	✓
- predictive of related variables	X	X	X	X	X	✓	✓	X	✓	X	X	X	X	✓	✓	✓✓	X	✓	X	✓✓	X	X	✓	✓	✓	X	X	✓	✓✓	X	X	X	✓	✓	✓	✓	✓	✓	X	X	✓
- association with other variables	X	X	X	✓	✓	✓	✓	X	✓	✓	✓	X	X	X	X	✓✓	X	✓	X	✓	X	X	✓	X	X	✓	X	✓	✓✓	X	✓	✓	✓	✓	X	✓	✓	✓	✓	✓	✓
- differences between groups	X	✓	X	X	X	X	X	X	X	✓	X	X	✓	X	X	X	X	X	X	X	X	X	X	X	X	X	X	✓	X	X	X	X	X	X	X	X	✓	X	X	X	✓
Structural																																									
- exploratory	X	X	X	X	X	✓	X	✓	X	X	✓	X	✓	✓	✓	✓	X	?	✓	✓	X	X	✓	X	✓	X	✓	✓	✓	X	X	X	✓	✓	✓	✓	✓	✓	✓	X	✓
- confirmatory	X	X	X	X	X	X	X	X	X	X	X	X	✓	X	X	X	X	?	X	X	X	X	✓	X	X	X	X	X	✓	X	X	X	X	✓	X	X	X	✓	X	X	✓
Reliability																																									
Internal consistency	X	X	X	X	✓	✓	✓	✓	✓	✓	X	X	✓	✓	✓	✓	X	✓	X	✓	X	✓	✓	X	✓	✓	✓	✓	✓✓	X	✓	✓	✓	✓	✓	✓	✓	✓	✓	✓	✓
Stability over time	X	X	X	X	X	X	X	X	X	X	X	X	X	X	X	X	X	X	X	X	X	X	X	X	X	X	X	X	X	X	X	X	X	X	X	X	X	X	X	X	X
Other	X	X	X	X	X	X	X	X	X	X	X	X	X	X	✓	X	X	X	✓	X	X	✓	X	X	X	X	X	X	X	X	X	X	X	X	X	X	X	X	X	X	X
Other assessments																																									
Acceptability (assessed or inferred - RR, missing items)	X	X	X	X	X	X	X	✓	X	X	X	X	X	✓	X	X	X	X	X	X	✓	✓	✓	X	✓	✓	X	X	X	X	X	X	X	X	X	X	X	X	X	X	X
Feasibility (completion time, other assessments)	X	X	X	X	X	X	X	✓	X	X	X	X	X	X	X	✓	X	X	✓	✓	X	X	✓	X	✓	✓	X	X	✓	X	X	X	X	X	X	X	X	X	X	X	X
Level of analysis considered	X	X	✓	X	X	X	✓	✓	X	✓	X	✓	X	✓	✓✓	✓	X	✓	✓	✓✓	X	X	✓	✓	✓	X	X	✓✓	✓	X	✓	✓	X	✓	✓	✓	✓	✓	X	X	X
Interpretability (ceiling/floor effects; important change or difference)	X	X	X	X	X	X	X	X	X	X	X	X	X	X	X	✓	X	X	X	✓	X	X	✓	X	X	X	X	X	NA	X	X	X	X	X	X	X	✓	X	X	X	X
Responsiveness	X	X	X	X	X	X	X	X	X	X	X	X	X	X	X	X	X	X	X	X	X	X	X	X	X	X	X	X	X	X	X	X	X	X	X	X	X	X	X	X	X
Generalisability (sampling & RR reported)	✓	✓	✓	✓	✓	✓	✓	✓	✓	✓	✓	✓	✓	✓	✓	✓	✓	✓	X	✓	✓	✓	✓	✓	✓	✓	X	✓	✓	✓	✓	✓	✓	✓	✓	✓	✓	✓	✓	✓	✓

1 Scoring indicates the extent of evidence available, not methodological quality of assessments or adequacy of measurement properties. Assessments on which scoring is based are summarised in Tables 11-13 (Additional file 6).

2 Key ✓ - some description OR some assessment (e.g. brief description of construct, implicit theory, single administration), ✓✓ - comprehensive description (e.g. explicit construct definition) OR multiple assessments under relevant conditions, X - not assessed, ? – unclear, NA – not applicable (e.g. a priori hypotheses not applicable if no hypotheses were tested)

References

1. Apekey TA, McSorley G, Tilling M, Siriwardena AN: **Room for improvement? Leadership, innovation culture and uptake of quality improvement methods in general practice.** *J Eval Clin Pract* 2011, **17:**311-318.
2. Bobiak SN, Zyzanski SJ, Ruhe MC, Carter CA, Ragan B, Flocke SA, Litaker D, Stange KC: **Measuring practice capacity for change: a tool for guiding quality improvement in primary care settings.** *Qual Manag Health Care* 2009, **18:**278-284.
3. Book S, Hellstrom A, Olsson J: **Perceptions of improvement work in Swedish health care: implications for improvement practices.** *Qual Manag Health Care* 2003, **12:**217-224.
4. Geboers H, Mokkink H, van Montfort P, van den Hoogen H, van den Bosch W, Grol R: **Continuous quality improvement in small general medical practices: the attitudes of general practitioners and other practice staff.** *Int J Qual Health Care* 2001, **13:**391-397.
5. Kralewski J, Dowd BE, Kaissi A, Curoe A, Rockwood T: **Measuring the culture of medical group practices.** *Health Care Manage Rev* 2005, **30:**184-193.
6. Leykum L, Palmer R, Lanham H, Jordan M, McDaniel R, Noel P, Parchman M: **Reciprocal learning and chronic care model implementation in primary care: results from a new scale of learning in primary care settings.** *BMC Health Serv Res* 2011, **11:**44.
7. Marsden PV, Landon BE, Wilson IB, McInnes K, Hirschhorn LR, Ding L, Cleary PD: **The reliability of survey assessments of characteristics of medical clinics.** *Health Serv Res* 2006, **41:**265-283.
8. Ohman-Strickland PA, John Orzano A, Nutting PA, Perry Dickinson W, Scott-Cawiezell J, Hahn K, Gibel M, Crabtree BF: **Measuring organizational attributes of primary care practices: development of a new instrument.** *Health Serv Res* 2007, **42:**1257-1273.
9. Olsson J, Kammerlind P, Thor J, Elg M: **Surveying improvement activities in health care on a national level--the Swedish internal collaborative strategy and its challenges.** *Qual Manag Health Care* 2003, **12:**202-216.
10. Parker VA, Wubbenhorst WH, Young GJ, Desai KR, Charns MP: **Implementing quality improvement in hospitals: the role of leadership and culture.** *Am J Med Qual* 1999, **14:**64-69.
11. Rushmer RK, Kelly D, Lough M, Wilkinson JE, Greig GJ, Davies HT: **The Learning Practice Inventory: diagnosing and developing Learning Practices in the UK.** *J Eval Clin Pract* 2007, **13:**206-211.
12. Solberg LI, Asche SE, Margolis KL, Whitebird RR: **Measuring an organization's ability to manage change: the change process capability questionnaire and its use for improving depression care.** *Am J Med Qual* 2008, **23:**193-200.
13. Shortell SM, Rousseau DM, Gillies RR, Devers KJ, Simons TL: **Organizational assessment in intensive care units (ICUs): construct development, reliability, and validity of the ICU nurse-physician questionnaire.** *Med Care* 1991, **29:**709-726.
14. Stevenson K, Baker R: **Investigating organisational culture in primary care.** *Qual Prim Care* 2005, **13:**191-200.
15. Sylvester S: **Measuring the learning practice: diagnosing the culture in general practice.** *Qual Prim Care* 2003, **11:**29-40.
16. Duckers MLA, Wagner C, Groenewegen PP: **Developing and testing an instrument to measure the presence of conditions for successful implementation of quality improvement collaboratives.** *BMC Health Serv Res* 2008, **8:**172.
17. Estabrooks C, Squires J, Cummings G, Birdsell J, Norton P: **Development and assessment of the Alberta Context Tool.** *BMC Health Serv Res* 2009, **9:**234.
18. Fedor DB, Fedor DB, Caldwell S, Herold DM: **The effects of organizational changes on employee commitment: a multilevel investigation.** *Pers Psychol* 2006, **59:**1-29.
19. Freeman T: **Measuring progress in clinical governance: assessing the reliability and validity of the Clinical Governance Climate Questionnaire.** *Health Serv Manage Res* 2003, **16:**234-250.

20. Gustafson DH, Sainfort F, Eichler M, Adams L, Bisognano M, Steudel H: **Developing and testing a model to predict outcomes of organizational change.** *Health Serv Res* 2003, **38:**751-776.
21. Helfrich CD, Li YF, Sharp ND, Sales AE: **Organizational readiness to change assessment (ORCA): Development of an instrument based on the Promoting Action on Research in Health Services (PARiHS) framework.** *Implement Sci* 2009, **4:**38.
22. Ingersoll GL, Kirsch JC, Merk SE, Lightfoot J: **Relationship of organizational culture and readiness for change to employee commitment to the organization.** *J Nurs Adm* 2000, **30:**11-20.
23. Kivimaki M, Maki E, Lindstrom K, Alanko A, Seitsonen S, Jarvinen K: **Does the implementation of Total Quality Management (TQM) change the wellbeing and work-related attitudes of health care personnel? Study of a TQM prize-winning surgical clinic.** *J Organ Change Manag* 1997, **10:**456-470.
24. Lageson C: **Brief report: reliability and validity of the TQManager feedback instrument.** *J Healthc Qual* 2006, **28:**45-49.
25. Lameei A: **Assessment of organization readiness for TQM implementation.** *Iran J Public Health* 2005, **34:**58-63.
26. Lehman WE, Greener JM, Simpson DD: **Assessing organizational readiness for change.** *J Subst Abuse Treat* 2002, **22:**197-209.
27. Levesque DA, Prochaska JM, Prochaska JO, Dewart SR, Hamby LS, Weeks WB: **Organizational stages and processes of change for continuous quality improvement in health care.** *Consulting Psychol J* 2001, **53:**139-153.
28. Marchionni C, Ritchie J: **Organizational factors that support the implementation of a nursing best practice guideline.** *J Nurs Manag* 2008, **16:**266-274.
29. Meurer SJ, Rubio DM, Counte MA, Burroughs T: **Development of a healthcare quality improvement measurement tool: results of a content validity study.** *Hosp Top* 2002, **80:**7-13.
30. Nelson EC, Batalden PB, Huber TP, Mohr JJ, Godfrey MM, Headrick LA, Wasson JH: **Microsystems in health care: Part 1. Learning from high-performing front-line clinical units.** *Jt Comm J Qual Improv* 2002, **28:**472-493.
31. Olsson JA, Ovretveit J, Kammerlind P: **Developing and testing a model to predict outcomes of organizational change.** *Qual Manag Health Care* 2003, **12:**240-249.
32. Oudejans SC, Schippers GM, Schramade MH, Koeter MW, van den Brink W: **Measuring the learning capacity of organisations: development and factor analysis of the Questionnaire for Learning Organizations.** *Qual Saf Health Care* 2011.
33. Øvretveit J: **Change achievement success indicators (CASI)** [http://www.qualityhealthcare.org]
34. Rondeau KV, Wagar TH: **Organizational learning and continuous quality improvement: examining the impact on nursing home performance.** *Healthc Manage Forum* 2002, **15:**17-23.
35. Rondeau KV, Wagar TH: **Implementing CQI while reducing the work force: how does it influence hospital performance?** *Healthc Manage Forum* 2004, **17:**22-29.
36. Schouten L, Grol R, Hulscher M: **Factors influencing success in quality improvement collaboratives: development and psychometric testing of an instrument.** *Implement Sci* 2010, **5:**84.
37. Shortell SM, Jones RH, Rademaker AW, Gillies RR, Dranove DS, Hughes EF, Budetti PP, Reynolds KS, Huang CF: **Assessing the impact of total quality management and organizational culture on multiple outcomes of care for coronary artery bypass graft surgery patients.** *Med Care* 2000, **38:**207-217.
38. Siloaho M, Hukkanen K, Kinnunen J: **Key elements of the implementation of a quality system in three Finnish clinical laboratories.** *Clin Chem Lab Med* 2001, **39:**988-996.
39. West TD: **Comparing change readiness, quality improvement, and cost management among Veterans Administration, for-profit, and nonprofit hospitals.** *J Health Care Finance* 1998, **25:**46-58.

40. Westphal JD, Gulati R, Shortell SM: **Customization or conformity? An institutional and network perspective on the content and consequences of TQM adoption.** *Adm Sci Q* 1997, **42:**366-394.
41. Zammuto RF, Krakower JY: **Quantitative and qualitative studies of organizational culture.** In *Research in organizational change and development. Volume* 5. Edited by Pasmore WA, Woodman RW: Emerald Group Publishing Limited; 1991: 83-114
42. Ahire SL, Golhar DY, Waller MA: **Development and validation of TQM implementation constructs.** *Decision Sci* 1996, **27:**23-55.
43. Armenakis AA, Bernerth JB, Pitts JP, Walker HJ: **Organizational change recipients' beliefs scale: development of an assessment instrument.** *J Appl Behav Sci* 2007, **43:**481-505.
44. Bouckenooghe D, Devos G, van den Broeck H: **Organizational Change Questionnaire-Climate of Change, Processes, and Readiness: development of a new instrument.** *J Psychol* 2009, **143:**559-599.
45. Dabhilkar M, Bengtsson L, Bessant J: **Convergence or national specificity? Testing the CI maturity model across multiple countries.** *Creat Innov Man* 2007, **16:**348-362.
46. Fisher CM, Barfield J, Li J, Mehta R: **Retesting a model of the Deming management method.** *Total Qual Manag Bus* 2005, **16:**401-412.
47. Jansen KJ: **From persistence to pursuit: A longitudinal examination of momentum during the early stages of strategic change.** *Organ Sci* 2004, **15:**276-294.
48. Holt DT, Armenakis AA, Field HS, Harris SG: **Readiness for organizational change.** *J Appl Behav Sci* 2007, **43:**232-255.
49. Taveira AD, James CA, Karsh BT, Sainfort F: **Quality management and the work environment: an empirical investigation in a public sector organization.** *Appl Ergon* 2003, **34:**281-291.
50. Zeitz G, Johannesson R, Ritchie JE, Jr.: **An employee survey measuring total quality management practices and culture: development and culture.** *Group Organ Manage* 1997, **22:**414-431.
51. Calomeni CA, Solberg LI, Conn SA: **Nurses on quality improvement teams: how do they benefit?** *J Nurs Care Qual* 1999, **13:**75-90.
52. Lin MK, Marsteller JA, Shortell SM, Mendel P, Pearson M, Rosen M, Wu SY: **Motivation to change chronic illness care: results from a national evaluation of quality improvement collaboratives.** *Health Care Manage Rev* 2005, **30:**139-156.
53. Solberg LI, Brekke ML, Kottke TE, Steel RP: **Continuous quality improvement in primary care: what's happening?** *Med Care* 1998, **36:**625-635.
54. Balding C: **Embedding organisational quality improvement through middle manager ownership.** *Int J Health Care Qual Assur Inc Leadersh Health Serv* 2005, **18:**271-288.
55. Cunningham CE, Woodward CA, Shannon HS, MacIntosh J, Lendrum B, Rosenbloom D, Brown J: **Readiness for organizational change: A longitudinal study of workplace, psychological and behavioural correlates.** *J Occup Organ Psychol* 2002, **75:**377-392.
56. Francois P, Vinck D, Labarere J, Reverdy T, Peyrin JC: **Assessment of an intervention to train teaching hospital care providers in quality management.** *Qual Saf Health Care* 2005, **14:**234-239.
57. Gould BE, Grey MR, Huntington CG, Gruman C, Rosen JH, Storey E, Abrahamson L, Conaty AM, Curry L, Ferreira M, et al: **Improving patient care outcomes by teaching quality improvement to medical students in community-based practices.** *Acad Med* 2002, **77:**1011-1018.
58. Herscovitch L, Meyer JP: **Commitment to organizational change: extension of a three-component model.** *J Appl Psychol* 2002, **87:**474-487.
59. Hill A, Gwadry-Sridhar F, Armstrong T, Sibbald WJ: **Development of the continuous quality improvement questionnaire (CQIQ).** *J Crit Care* 2001, **16:**150-160.
60. Irvine D: **The development of measures of organizational citizenship behaviour and changes in job behaviours related to quality management in health care.** *Health Serv Manage Res* 1995, **8:**143-161.

61. Irvine D, Leatt P, Evans MG, Baker RG: **Measurement of staff empowerment within health service organizations.** *J Nurs Meas* 1999, **7:**79-96.
62. Miller DK, Coe RM: **Physician participation in TQM in geriatric medicine.** *Jt Comm J Qual Improv* 2000, **26:**466-475.
63. Ogrinc G, Headrick LA, Morrison LJ, Foster T: **Teaching and assessing resident competence in practice-based learning and improvement.** *J Gen Intern Med* 2004, **19:**496-500.
64. Campbell D: **Embracing change: Examination of a 'capabilities and benevolence' beliefs model in a sample of military cadets.** *Mil Psychol* 2006, **18:**131-148.
65. Coyle-Shapiro JAM, Morrow PC: **The role of individual differences in employee adoption of TQM orientation.** *J Vocat Behav* 2003, **62:**320-340.
66. Hanpachern C, Morgan GA, Griego OV: **An extension of the theory of margin: a framework for assessing readiness for change.** *Hum Resour Dev Q* 1998, **9:**339-350.
67. Spreitzer GM: **Psychological empowerment in the workplace: dimensions, mesurement and validation.** *Acad Manage J* 1995, **38:**1442-1465.
68. Wanberg CR, Banas JT: **Predictors and outcomes of openness to changes in a reorganizing workplace.** *J Appl Psychol* 2000, **85:**132-142.
69. Engels Y, van den Hombergh P, Mokkink H, van den Hoogen H, van den Bosch W, Grol R: **The effects of a team-based continuous quality improvement intervention on the management of primary care: a randomised controlled trial.** *Br J Gen Pract* 2006, **56:**781-787.
70. Barsness ZI, Shortell SM, Gillies EFX, Hughes JL, O'Brien D: **The quality march. National survey profiles quality improvement activities.** *Hosp Health Netw* 1993, **67:**52-55.
71. Lemieux-Charles L, Murray M, Baker G, Barnsley J, Tasa K, Ibrahim S: **The effects of quality improvement practices on team effectiveness: a mediational model.** *J Organ Behav* 2002, **23:**533-553.
72. Wilkens R, London M: **Relationships between climate, process, and performance in continuous quality improvement groups.** *J Vocat Behav* 2006, **69:**510-523.
