# Supplementary material for: Measuring organizational and individual factors thought to influence the success of quality improvement in primary care: a systematic review of instruments
Source: Implement Sci. 2012 Dec 17;7:121. doi: 10.1186/1748-5908-7-121 (PMC3573896; doi:10.1186/1748-5908-7-121)
Supplement: Additional file 5 — Tables summarising the characteristics of instruments included for review of measurement properties (Tables S7-S9). [file 1748-5908-7-121-S5.pdf]

Additional file 5 – Tables summarising the characteristics of instruments included for review of measurement properties

Table 7: Characteristics of instruments measuring CQI use and implementation [1]

**Instrument** (name, index, references for main reports)	**Main purpose** (measurement aim and context of use; examples of use in health care)	**Description of content** (dimensions, sub-dimensions; conceptual/theoretical basis)	**Items & response scale**	**Study design(s)**	**Comments**
Primary care settings					
Quality improvement methods	Descriptive/outcome: To measure the association between leadership and adoption of QI methods in primary care.	Use of QI tools and techniques. Examples include: PDSA, root cause analysis, process mapping, process redesign, run/control charts, and cause and effect diagrams. Each tool rated according to 1) frequency of use and 2) extent to which use of the tool is perceived to have led to changes in practice.	44 items (2 per tool); 5 point Likert scale.	Observational - analytical, cross sectional	
Apekey, 2011a [1]	Other examples: none identified	Conceptual basis of measure not explicitly reported.			
Quality improvement activities	Outcome: Post-intervention measurement of use of CQI in general practices participating in a RCT of CQI. (Netherlands)	CQI steps undertaken: baseline measurement; targets set; barrier identification; change plan; results measurement	5 item checklist	RCT	
Engels, 2006 [2]	Other examples: none identified	Based on elements of the CQI model tested in this study, which was derived from selected QI literature (e.g. [3-4]).			
Attitudes toward CQI model	Descriptive: Assessment of participant response to introduction of CQI model tailored to general practice (Netherlands)	Attitudes in response to use of CQI model: usefulness of, perceived success with and willingness to continue using each of six elements.	18 items; 5 or 4 point Likert scale	Process evaluation	Comprehensive measure of attitudinal response to CQI intervention.
Geboers, 2001a [5]	Other examples: none identified	Based on CQI model developed by the authors [4].			
Process improvement progress (PIP)	Outcome: Pre- and post-intervention measurement of use of CQI concepts and techniques in primary care practices participating in a RCT of CQI. (USA)	Use of CQI concepts and techniques: 17 element of CQI process (training, team composition, meeting frequency, data collection and analysis, problem identification and solving, implementation)	17 items; 5 point Likert scale	RCT	
Solberg, 1998a [6-7]	Other examples: none identified	Based on published descriptions of recommended process improvement tasks from selected literature (e.g.[8-9]). Intended to characterize an "ideal generic team effort at process improvement" ([6] p625) not a specific model.			
Change process capability questionnaire (CPCQ)	Predictive/diagnostic: Assessment of organisational capability for managing change in primary care, with a focus on clinical process improvement. (USA)	Capability for managing change/QI: previous history of change; plans for continuous organizational refinement; ability to initiate and sustain change (16 items). Use of QI strategies for improving care, for example rapid cycle change, systems change. (14 items, 1 per strategy).	30 items; 5 point Likert scale.	Instrument development	Covers two domains: CQI use and organisational context.
Solberg, 2008 [10]	Other examples: pre- and post- intervention measurement of use of change process factors and strategies in primary care practices	Based on primary care practice change model [12] informed by model in [13]. QI strategies from [14]		Multiple baseline time series	

**Instrument** (name, index, references for main reports)	**Main purpose** (measurement aim and context of use; examples of use in health care)	**Description of content** (dimensions, sub-dimensions; conceptual/theoretical basis)	**Items & response scale**	**Study design(s)**	**Comments**
	participating in a QIC [11]. (USA)				
Other health care settings					
CQI/TQM adoption indicators	Discriminative/predictive/outcome: Classification of hospitals as adopters or non-adopters of CQI/TQM. (USA)	Organisational adoption of CQI/TQM: structured problem solving, statistical methods and measurement; philosophy of continuous improvement of processes; empowerment of employees; focus on customers; use of cross-disciplinary QI teams	5 items, 5 point Likert scale [15-16].	Observational - analytical, cross sectional	Indicators may suitable for use as a criterion measure of CQI adoption, with minor rewording for small practice settings, but further assessment for this purpose required.
Barsness, 1993 [17]	Other examples: Measure of QI adoption [3] and one of four variables used to measure QI intensity, as predictors of quality of care in hospitals [15-16]. Used to categorise nursing facilities to compare characteristics of CQI adopters and non-adopters [18-20]. Dependent variable in one study in hospitals [21]. (USA)	Based on selected QI literature [8, 22-24].	Dichotomous scale in most other studies.		
Supportive conditions for QI collaborative (QIC) implementation	See Duckers, 2008 [25] under 'CQI use' domain – Table 6				
Organizational Change Manager (OCM)	See Gustafson, 2003 [26] under 'organisational context' domain – Table 6				
Organizational readiness to change assessment (ORCA)	See Helfrich, 2009 [27] under 'organisational context' domain – Table 6				
QI practices index	Predictive: To measure use of QI practices as a predictor of QI team performance in hospital QI teams (Canada)	Use of key QI practices: knowledge of customer requirements, supplier partnerships, use of statistical methods and analyses, use of process management heuristics, use of cross-functional teams.	8 items, nominal response	Observational - analytical, cross sectional	
Lemieux-Charles, 2002 [28]	Other examples: none identified	Based on selected QI literature [3] [29-30]			
Continuous quality improvement questionnaire	See Meurer, 2002 [31] under 'organisational context' domain - Table 6				
Factors influencing success in a QIC	Predictive/diagnostic: To measure potential determinants of QIC success. The authors suggest retrospective use in QIC evaluations or prospective use by change agents as a checklist to optimise chances of success. (Netherlands)	Factors influencing success in a QIC: sufficient expert panel support; effective multidisciplinary teamwork (incorporating use of improvement model); helpful collaborative processes.	40 items, 5 point Likert scale	Instrument development	Covers two domains: CQI use and organisational context. Effective multidisciplinary teamwork scale suitable for evaluation of any team-based QI effort.
Schouten, 2010 [32]	Other examples: none identified	Based on systematic search for papers on QIC theory (e.g. papers [33-35])			

**Instrument** (name, index, references for main reports)	**Main purpose** (measurement aim and context of use; examples of use in health care)	**Description of content** (dimensions, sub-dimensions; conceptual/theoretical basis)	**Items & response scale**	**Study design(s)**	**Comments**
CQI process facilitation	Predictive: To measure facilitator structured group process as a predictor of perceived QI team performance in hospitals. (USA)	Facilitator role in CQI process: extent of involvement in the QI process (provision of training, fostering feedback, conflict resolution)	25 items, 7 point response scale	Observational – analytical, cross sectional	Comprehensive measure for assessing fidelity of facilitator role.
Wilkens, 2006 [36]	Other examples: none identified	Conceptual basis of measure not explicitly reported. Description of facilitator role based on single reference [37].			

1. Abbreviations: QI – quality improvement; QIC – quality improvement collaborative; RCT – randomised controlled trial; CCM – chronic care model; PDSA – plan do study act cycles

Table 8: Characteristics of instruments measuring organisational context [1]

**Instrument** (name, index, references for main reports)	**Main purpose** (measurement aim and context of use; examples of use in health care)	**Description of content** (dimensions, sub-dimensions; conceptual/theoretical basis)	**Items & response scale**	**Study design(s)**	**Comments**
Primary care settings					
Practice capacity for change	Predictive/diagnostic: To assess capacity for change and successful achievement of QI goals in primary care practices. (USA)	Practice capacity for change: practice members' motivations; external influences; resources for change; perceived options for change; organisational structure; climate; culture.	25 items, 5 or 6 point Likert	Instrument development	In this paper, scoring is based on researcher rating using data from direct observation and interview, not self report.
Bobiak, 2009 [38]	Other examples: none identified	Based on models of practice change in primary care [12, 39-40] and selected empirical studies.			
Perceptions of improvement work	Descriptive: To measure collective perceptions of improvement work among managers of healthcare organisations including primary care. (Sweden)	Perceptions of improvement work: effective approach that meets perceived needs; conflict between improvement work and the organisation; difficulties in the use of improvement practices and techniques.	9 items, 5 point Likert scale	Cross sectional survey	
Book, 2003 [41-42]	Other examples: none identified	Based on diffusion of innovation theory, specifically five attributes thought to influence diffusion (relative advantage; compatibility; complexity; observability; trialability).			
Obstacles to the use of CQI	Descriptive: Assessment of practical and personal barriers to use of CQI model tailored to general practice (Netherlands)	Obstacles to the use of CQI: practical factors (capacity and time; conflicting demands; staff participation; workload associated with model); individual level factors (beliefs about the complexity and usefulness of the model; competing demands; motivation; capability)	22 items, 5 point Likert scale	Process evaluation	Covers two domains: organisational context and individual level factors.
Geboers, 2001b [5]	Other examples: none identified	List of barriers derived from selected literature [43-44] and unpublished thesis.			
Medical Group Practice Culture	Predictive/discriminative: To assess the organisational culture of medical group practices (USA)	Medical group practice culture: collegiality; information; quality emphasis; organisational identity; organisational trust; cohesiveness; business; innovativeness; autonomy.	33 items, 4 point Likert scale	Instrument development	Minor rewording may be required for non-USA settings. A short 10 item version has been used in UK primary care [45]
Kralewski, 2005 [46-48]	Other examples: Instrument has been used to investigate the relationship between culture and aspects of practice performance, for example medication error [49] and the quality of diabetes care [45] (USA and UK respectively)	Based on dimensions of culture identified by Reynolds [50].			
Reciprocal learning scale	Predictive: To measure reciprocal learning as a factor thought to influence practice capacity to improve the quality of care.	Reciprocal learning. Items were intended to measure six themes, covering whether learning is reflective, proactive, continuous, shared, mindful, and sources of learning.	5 items, 5 point Likert scale	Observational – analytical, cross sectional	Data reported were baseline data for a randomised trial of a practice facilitation intervention to improve diabetes care.

Instrument (name, index, references for main reports)	Main purpose (measurement aim and context of use; examples of use in health care)	Description of content (dimensions, sub-dimensions; conceptual/theoretical basis)	Items & response scale	Study design(s)	Comments
Leykum, 2011 [51]	Other examples: none identified	Based on review and thematic analysis of organisational learning and educational psychology literatures, focusing on that where learning was conceived as a social activity in groups and organisations.			
Openness to QI and climate for QI participation	Predictive: Pre-intervention measurement of organisational-level characteristics to identify factors predictive of the number and comprehensiveness of QI activities in primary care practices participating in a QIC (USA)	Organisational culture: organisation QI focus; openness of organisational culture (includes items about leadership commitment to QI, organisational and interpersonal climate that supports experimentation).	9 items, 4 point response scale	Observational – analytical, longitudinal	Data collected as part of larger controlled pre- and post-intervention evaluation of a QI collaborative [52], however data reported here are observational.
Marsden, 2006 [53-54]	Other examples: none identified	Based on selected references on QI leadership (e.g.[3, 55]) and supportive organisational and interpersonal climate (e.g.[56-57])			
Survey of Organizational Attributes for Primary Care (SOAPC)	Predictive/discriminative: To characterise and discriminate between primary care practices based on their resources for change. (USA)	Internal resources for change: communication; decision making; stress/chaos; history of change (includes items about relationships among practice members; leadership and decision-making approaches; communication and perception of competing demands). Knowledge management and human resources scales added in version reported in [58]. Some items dropped from existing scales and some new items added to this version.	21 items, 5 point Likert scale<br><br>16 item version, [58]	Instrument development	SOAPC developed for ULTRA trial, which tested a team building intervention to improve guideline adherence.
Ohman-Strickland, 2007 [58-59]	Other examples: Selected scales used to investigate association between organisational attributes in primary care and preventive services [60-61], use of nurse practitioners for diabetes care [62], and productivity and turnover {Hung, 2006 #6616}. (USA)	Based on [39] primary care change model. Revisions based on knowledge management model developed for healthcare [63].		Observational – analytical, cross sectional	Multiple publications arising from ULTRA using SOAPC or subscales.
Climate for improvement	Descriptive: To measure perceptions of climate for (quality) improvement among managers of healthcare organisations including primary care (Sweden)	Climate for improvement: no sub-dimensions specified. Items focus on employee involvement, commitment, support for experimentation.	19 items, 5 point Likert scale	Observational – descriptive, cross sectional	Only 12 of 19 items reported.
Olsson, 2003b [42]	Other examples: none identified	Based on organisational change literature, but sources not referenced in this paper.			
Degree of QI implementation	Outcome: Dependent variable in study investigating whether leadership and culture are predictive of degree of QI implementation in hospitals. (USA)	Degree of QI implementation: role of managers; information and analysis; strategic quality planning; human resources development and management; management of process quality.	42 items, 5 point Likert scale	Observational – analytical, longitudinal	Content of this and [55] are similar. Instrument administered to measure QI implementation in all care settings in the VHA All Employee Survey.
Parker, 1999 [64]	Other examples: Used to examine associations between QI commitment and cancer screening in primary care [65], and QI implementation and quality of care in other settings [66] [67] (USA)	Based on Baldrige Quality Award criteria [68].			Data from example studies derived from this survey.

**Instrument** (name, index, references for main reports)	**Main purpose** (measurement aim and context of use; examples of use in health care)	**Description of content** (dimensions, sub-dimensions; conceptual/theoretical basis)	**Items & response scale**	**Study design(s)**	**Comments**
Learning Practice Inventory (LPI)	Diagnostic: To assess the extent to which primary care practice staff perceive that their practice possesses the characteristics of a learning organisation. (UK)	Characteristics of a learning practice: foundations (ownership and involvement; supporting attitudes and beliefs); support (supporting hard structures and organisational arrangements; supporting behaviours; supporting systems); progress (evidence of learning routines; outcomes associated with learning practices; maintaining momentum)	62 items, 10 point modified Behaviourally Anchored Rating Scale (BARS)	Instrument development	
Rushmer, 2007 [69-71]	Other examples: none identified	Based on literature review of learning organisations and organisational learning theory and its application to healthcare settings [72-74].			
Change process capability questionnaire (CPCQ)	See Solberg, 2008 [10] under 'CQI use' domain – Table 5				
Organizational assessment in ICU measure	Predictive/outcome: To measure managerial and organisational processes as i) potential predictors of healthcare quality and efficiency and ii) outcomes of interventions aiming to improve organisational factors.	Managerial and organisational processes (five constructs): leadership (emphasis on excellence, clear goals, responsive to needs, understanding of staff concerns); communication (openness, accuracy, timeliness, understanding, satisfaction); coordination (plans, treatment protocols, policies and procedures, unit director's efforts, face-to-face interaction); problem-solving/conflict management (collaborative problem-solving, arbitration approaches); unit cohesiveness.<br><br>Based on theoretical framework derived from organisational behaviour literature (multiple references for each construct).	56 items, 5 point Likert scale.<br><br>42 item, 5 point Likert scale version for primary care.	Instrument development	Several modifications exist for other settings; most relevant to this review is the version for primary care [75]. The original instrument has been widely used, particularly in hospital settings (170+ citations).
Shortell, 1991 [75-76]	Other examples: Version for primary care [75], used as an outcome measure in a randomized trial of a guideline implementation strategy [77-78].				
Practice Culture Questionnaire (PCQ)	Diagnostic: To identify cultures resistant to QI activities in primary care teams. (UK)	Organisational culture for clinical governance: being responsible and accountable for quality clinical care; involving all staff in QI activity; developing appropriate risk management procedures; developing appropriate procedures to identify and remedy poor performance	25 items, 4 point scale	Instrument development	Some items specific to UK model of clinical governance.
Stevenson, 2005 [79-80]	Other examples: none identified	Based on previously reported elements of a resistant culture [81].			
Learning organisation characteristics questionnaire	Diagnostic: To use a learning organisation diagnostic tool to ascertain the organisational culture of general practices (UK)	Characteristics of a learning organisation: learning; people in the practice; creativity; values and beliefs; change; feedback; connectedness; teamwork.	40 items, 5 point Likert scale	Instrument development	
Sylvester, 2003 [82]	Other examples: Instrument was modified for use in a single practice [83]; study reports descriptive data only. (UK)	Based on management literature on learning organisations [84-86].			

Instrument (name, index, references for main reports)	Main purpose (measurement aim and context of use; examples of use in health care)	Description of content (dimensions, sub-dimensions; conceptual/theoretical basis)	Items & response scale	Study design(s)	Comments
Other health care settings					
Supportive conditions for QI collaborative (QIC) implementation	Discriminative/predictive: Measurement of QIC implementation as a predictor of process and outcome improvement in QI teams participating in a national, hospital based QIC. (Netherlands)	Implementation of elements of a QIC: QIC team organisation; external change agent support; organisational support for teams.	15 items, 7 point Likert scale	Instrument development	Covers two domains: CQI use and organisational context. Although designed for QICs, most items are suitable for any team-based QI.
Duckers, 2008 [25]	Other examples: additional reports from the same QIC evaluation [87-88].	Based on literature pertaining to QIC success, team performance, and the role of leaders and change agents' in supporting adoption of innovations.		Pre- and post-intervention evaluation	
Organizational Change Manager (OCM)	Diagnostic/predictive: To enhance chances of successful change by measuring modifiable factors that predict success of health care improvement projects. (USA)	Modifiable factors that predict implementation of a health system change: mandate; leader and middle manager goals, involvement and support; supporters and opponents; tension for change; staff needs assessment, involvement and support; exploration of problem and customer needs; change agent prestige, commitment and customer focus; source of ideas; funding; advantage to staff and customers; radicalness of design; flexibility of design; evidence of effectiveness; complexity of implementation plans; work environment; staff changes required; monitoring and feedback.	18 items, three descriptions for each item - respondents choose one that best fits their organisation.	Model development<br><br>Observational-analytical, longitudinal	Instrument provides a tool for assessing readiness for a specific change. Although not specific to CQI, it is a comprehensive measure of change process. Hence, the OCM is included in the CQI use and organisational context domains.
Gustafson, 2003 [26]	Other examples: Used to predict QI outcomes in teams participating in a hospital-based QIC [89] and a primary care based QIC [90].(USA)	Based on review of organisational change models and literature and expert-consensus model developed in this study.			
Organizational readiness to change assessment (ORCA)	Diagnostic/predictive: "…to assess organizational readiness to change in preparation for testing interventions designed to implement evidence-based changes in clinical practice" (p2) (USA)	Organisational readiness to change: evidence (discord, research, clinical experience, patient preferences); context (leader culture; staff culture; leadership behaviour; measurement; opinion leaders; general resources; facilitation (leaders practices; clinical champion; leadership implementation role; implementation team roles; implementation plan; project communication; project tracking; project resources and context; project evaluation)	73 items, 5 point Likert scale	Instrument development	Context scale is most relevant of three scales for CQI evaluation. Although not specific to CQI, the facilitation scale is a comprehensive measure of change process, hence ORCA is also included in the CQI use domain.
Helfrich, 2009 [27]	Other examples: Used to examine association between readiness and introduction of preventive care in substance use disorder clinics [91].	Based on PAHRiS framework [92]. QI implementation survey [55] informed context domains.			
TQManager feedback instrument	Unclear, potentially predictive: To measure managerial competences for TQM in healthcare settings. (USA)	Managerial competencies for supporting QM: developing relationships of openness and trust; building collaboration and teamwork; managing by fact; supporting results through recognition and rewards; learning values.	25 items, 5 point Likert scale	Instrument development	
Lageson, 2006 [93]	Other examples: none identified	Based on core competencies described by quality experts (e.g.[23, 94-95]).			

Instrument (name, index, references for main reports)	Main purpose (measurement aim and context of use; examples of use in health care)	Description of content (dimensions, sub-dimensions; conceptual/theoretical basis)	Items & response scale	Study design(s)	Comments
Continuous quality improvement questionnaire	Discriminative: To measure the extent of implementation of QI philosophy and methods in hospitals. (USA)	Quality improvement implementation: leadership, process, human resources training, planning, information, customer focus, innovation, supplier partnership. Score used o assign level of implementation ranging from 1 (not yet an important topic) to 5 (absorbed).	22 items, 5 point Likert scale	Instrument development	Most items could be used in primary care with minor rewording.
Meurer, 2002 [31, 96]	Other examples: none identified	Based on quality award frameworks and review of existing instruments.			
Factors influencing success in a QIC	See Schouten, 2010 [32] under 'CQI use' domain – Table 5				
Quality improvement implementation survey II (QIIS)	Predictive: To measure the association between QI implementation and care outcomes in hospitals (USA).	Extent of QI deployment/implementation: leadership; information and analysis; employee quality planning involvement; employee quality training (labelled "human resource utilization" in some versions); quality results; quality management; customer satisfaction.	58 items, 5 point Likert scale<br>20 item version [97]	Observational – analytical, longitudinal	Content of this and Parker, 1999 [64] are similar. No primary care equivalent was identified. Minor rewording required for primary care.
Shortell, 2000 [3, 55]	Other examples: Used in a national evaluation of QICs [98] to measure commitment to QI as a predictor of motivation to run PDSA cycles and implement the Chronic Care Model [97], perceived QI team effectiveness and changes to care processes [99]. (USA)	Based on Baldrige Quality Award criteria [68].			The QIC evaluation used a controlled pre- post-intervention design [98], however reported data [97, 99]) are observational (from intervention group only).
Competing values instrument for organizational culture	Predictive/discriminative: Used in a wide range of studies investigating culture as a potential determinant of organisational performance and quality of care.	Organisational culture: typological measures used to categorise organisations by their dominant culture type(s). Culture type is based on four organisational characteristics: overall characteristics, leadership, cohesion and emphasis. For each characteristic, four descriptions are provided, each reflecting a different culture type (group, developmental rational or hierarchical culture). A fifth characteristic –rewards – is included in Shortell's version [3]. In the ipsative scale, 100 points are distributed across four descriptions according to fit with the respondent's organisation.	16 item, ipsative scale [100] [101]; Likert scale [101]<br>20 item, ipsative scale [3]<br>14 item,5 point Likert scale [102]	Instrument development	Identified as the most widely used measure of culture in health care [102]. The 14 item version is used in the Veterans Health Administration all employee survey, providing a large data set (>70,000) for validating this measure [102]
Zammuto, 1991 [3, 100-102]	Other examples: Used in a national evaluation of QICs to measure culture as a potential determinant of QI outcomes [97]. (USA) Used to measure the association between culture and quality of care in primary care [103] (UK), [104] (Netherlands), [65] (USA).	Based on the competing values framework [105] [106].		Observational – analytical, longitudinal	
Non health care					
Organizational Change Recipients' Beliefs Scale (OCRBS)	See Armenakis, 2007 [107] under 'individual factors' domain – Table 7				

**Instrument** (name, index, references for main reports)	**Main purpose** (measurement aim and context of use; examples of use in health care)	**Description of content** (dimensions, sub-dimensions; conceptual/theoretical basis)	**Items & response scale**	**Study design(s)**	**Comments**
Readiness for organisational change	See Holt, 2007 [108] under 'individual factors' domain – Table 7				

1. Abbreviations: QI – quality improvement; QIC – quality improvement collaborative; RCT – randomised controlled trial; CCM – chronic care model; PDSA – plan do study act cycles

Table 9: Characteristics of instruments measuring individual level factors [1]

Instrument (name, index, references for main reports)	Main purpose (measurement aim and context of use; examples of use in health care)	Description of content (dimensions, sub-dimensions; conceptual/theoretical basis)	Items & response scale	Study design(s)	Comments
Primary care settings					
Satisfaction, perceived skills and impact of CQI participation	Descriptive: To assess response to process improvement of nurses acting as leaders of QI teams participating in an RCT of CQI in primary care practices (USA)	Response to participation in process improvement: self-perceived skills in process improvement (17 items); satisfaction with involvement (13 items); perceived impact of involvement on the nursing role (6 items)	36 items, 5 or 6 point Likert scale	Process evaluation, observational data from cross sectional survey administered post-intervention	Items in scale measuring perceived impact are specific to nursing.
Calomeni, 1999 [109]	Other examples: none identified	Not reported. Items were based on the learning objectives of the training provided in the IMPROVE trial [110]			
Obstacles to the use of CQI	See Geboers, 2001b [5] under 'organisational context' domain – Table 6				
Motivation to run PDSA cycles and implement the CCM	Outcome/predictive/discriminative: Used in a national evaluation of QICs [98] to measure motivation as a potential determinant of system change and improvement in care quality. In [97], used as dependent variable examining the association between QI commitment and motivation. (USA)	Motivation: expectancy (association between effort and success implementing PDSA process); instrumentality (perceived link between purported outcomes and running PDSA cycles); valence (value assigned to the outcomes associated with implementation of CCM and running PDSA cycles)	18 items, 7 point Likert scale (expectancy, instrumentality) or 5 point Likert scale (valence)	Observational – analytical, longitudinal	The QIC evaluation used a controlled pre- post-intervention design [98], however data reported here are observational (from intervention group only).
Lin, 2005 [97-98]	Other examples: none identified	Based on expectancy theory [111-112]			
Provider attitude survey (PAS) - CQI scale	Descriptive: Pre-intervention measurement of knowledge, attitudes and beliefs about CQI in primary care practices recruited to a RCT of CQI. (USA)	Knowledge, attitudes and beliefs about CQI: knowledge of CQI and process improvement; beliefs about the relevance and importance of CQI principles and practices for improving care.	10 items, 5 point Likert scale	Cross sectional survey	
Solberg, 1998b [113]	Other examples: none identified	Conceptual basis not reported.			
Other health care settings					
Individual readiness for organizational change	Predictive: Measurement of readiness as a mediator of participation and contribution to change among staff in a hospital undergoing organisational re-engineering. (USA)	Individual readiness for change: precontemplative stage; contemplative stage; reparatory stage; action stage; maintenance stage.	6 items, 5 point Likert scale	Observational – analytical, longitudinal	
Cunningham, 2002 [114]	Other examples: Used to measure association between readiness for change and potentially modifiable factors among general practice staff enrolled in a RCT of an organisation change intervention [115] (Australia)	Based on stages of change model [116-117]		Cross sectional survey administered prior to RCT	

**Instrument** (name, index, references for main reports)	**Main purpose** (measurement aim and context of use; examples of use in health care)	**Description of content** (dimensions, sub-dimensions; conceptual/theoretical basis)	**Items & response scale**	**Study design(s)**	**Comments**
Commitment to change scales	Predictive: Used to examine the association between different forms of individual commitment to organisational change and behavioural support for change among registered nurses. (USA)	Commitment to change: affective commitment (support for the change based on a belief in its inherent benefits); continuance commitment (recognition of costs associated with failure to support change); normative commitment (obligation to provide support change).	18 items, 7 point Likert scale	Observational – analytical, cross sectional	Instrument has a strong theoretical basis and is a comprehensive measure of commitment to a specific change.
Herscovitch, 2002a [118-119]	Other examples: none identified in primary care or related to QI in healthcare.	Based Meyer and Herscovitch's three component model of commitment to organisational change [120].			
Behavioural support for change	Outcome: Used as a dependent variable to examine the association between individual commitment to organisational change and behavioural support for change among registered nurses. (USA)	Behavioural response in support of a change initiative: compliance (3 items); cooperation (8 items); championing (6 items). A second measure, using a behavioural continuum with five anchor points: active resistance; passive resistance; compliance; cooperation; championing. A written description of each anchor point was provided.	17 items, 7 point Likert scale<br><br>Single item behavioural continuum, 101 points with 5 anchors	Observational – analytical, cross sectional	Scales have potential application as an intermediate outcome measure.
Herscovitch, 2002b [118-119]	Other examples: none identified in primary care or related to QI in healthcare.	Based Meyer and Herscovitch's three component model of commitment to organisational change [120].			
Job behaviour related to CQI (JBCQI)	Outcome: Measurement of job behaviour related to QI as a behavioural outcome of participation in CQI teams in hospitals. (Canada)	Job behaviour related to CQI: problem solving; customer awareness; use of CQI knowledge; organisational interest.	24 items, 5 point Likert scale	Instrument development	
Irvine, 1995 [121-122]	Other examples: none identified	Based on new construct defined in this paper and data derived inductively from interviews.		Observational – analytical, longitudinal	
Empowerment	Outcome/predictive: Measurement of empowerment as an outcome of participation in CQI teams and as a mediator of job behaviour related to QI among hospital staff with experience on CQI teams. (Canada)	Empowerment (self efficacy perceptions): outcome empowerment (confidence in ability to influence outcomes); verbal empowerment (confidence in verbal discourse); behavioural empowerment (confidence in specific tasks)	21 items, 11 point response scale	Instrument development	Developed explicitly for use in studies of CQI - a comprehensive measure of empowerment for QI behaviours.
Irvine, 1999 [122-123]	Other examples: used to examine the association between organisational change factors and empowerment among nurses in hospitals [124] [125] (Finland)	Based on Conger and Kanungo's conceptualization of empowerment [126].		Observational – analytical, longitudinal	

**Instrument** (name, index, references for main reports)	**Main purpose** (measurement aim and context of use; examples of use in health care)	**Description of content** (dimensions, sub-dimensions; conceptual/theoretical basis)	**Items & response scale**	**Study design(s)**	**Comments**
Non-health care					
Organizational Change Recipients' Beliefs Scale (OCRBS)	Predictive/diagnostic: Measurement of change recipients' beliefs as a predictor of change outcomes; use as tool to monitor change progress and take action to improve outcomes.	Change recipients' beliefs (5 dimensions): discrepancy; appropriateness; efficacy; principal support; valence.	24 items, 7 point response scale	Instrument development	Developed for use in any change context, irrespective of setting.
Armenakis, 2007 [107]	Other examples: none identified	Based on comprehensive review and analysis of change recipients' beliefs from organisational science and innovation research literature.			
Individual TQM orientation	Outcome: Dependent variable in study in manufacturing sector exploring determinants of individual adoption of a TQM orientation. (UK)	Facets of continuous improvement: engagement in quality focused behaviour (active involvement); acceptance of quality and continuous improvement precepts (allegiance to quality); degree to which an individual feels responsible for quality (personal accountability).	11 items, 7 point Likert scale	Observational – analytical, longitudinal	Developed in manufacturing sector, however item wording is suitable for any context and no equivalent measures were identified from the healthcare.
Coyle-Shapiro, 2003 [127]	Other examples: none identified	Based on review of TQM literature (selected references cited).			
Readiness for change	Outcome: Dependent variable in study exploring association between personal and work related factors and readiness for organisational change, in individuals working in manufacturing sector. (USA)	Individual readiness for organisational change: participating in, promoting, and resisting organisational change (single scale)	14 items, 7 point Likert scale	Cross sectional survey	Developed in manufacturing sector, however item wording is suitable for any context and it is a comprehensive measure of intention to support change. No equivalent measures identified from healthcare.
Hanpachern, 1998 [128]	Other examples: No published studies in healthcare found (one thesis using measure in hospitals excluded).	The authors reported that the instrument was based on published and unpublished research, however only one reference was provided [129]			
Readiness for organizational change	Predictive/discriminative: the authors reported that the instrument could be used to evaluate an organisation change, with potential for use as a predictor of change outcomes and to discriminate between groups.	Readiness for organisational change: self efficacy for implementation of change (change confidence); belief that the change will be personally beneficial (personal valence); senior leader support for the change (management support); belief that the change will benefit the organisation (organisational valence); belief that change is needed (discrepancy)	25 items, 7 point response scale	Instrument development	Instrument has a strong theoretical basis, is suitable for any context and is a comprehensive measure of change readiness beliefs. No equivalent measures identified from the healthcare literature.
Holt, 2007 [108]	Other examples: The 'management support' and 'change appropriateness' scales were used to measure potential antecedents of clinicians' readiness to adopt clinical information systems [130] (Canada)	Based on a systematic review of existing instruments [131].			Covers two domains: organisational context and individual factors.

1. Abbreviations: QI – quality improvement; QIC – quality improvement collaborative; RCT – randomised controlled trial; CCM – chronic care model; PDSA – plan do study act cycles

References

1. Apekey TA, McSorley G, Tilling M, Siriwardena AN: **Room for improvement? Leadership, innovation culture and uptake of quality improvement methods in general practice.** *J Eval Clin Pract* 2011, **17:**311-318.
2. Engels Y, van den Hombergh P, Mokkink H, van den Hoogen H, van den Bosch W, Grol R: **The effects of a team-based continuous quality improvement intervention on the management of primary care: a randomised controlled trial.** *Br J Gen Pract* 2006, **56:**781-787.
3. Shortell SM, O'Brien JL, Carman JM, Foster RW, Hughes EF, Boerstler H, O'Connor EJ: **Assessing the impact of continuous quality improvement/total quality management: concept versus implementation.** *Health Serv Res* 1995, **30:**377-401.
4. Geboers H, Grol R, Van Den Bosch W, Van Den Hoogen H, Mokkink H, Van Montfort P, Oltheten H: **A model for continuous quality improvement in small scale practices.** *Qual Health Care* 1999, **8:**43-48.
5. Geboers H, Mokkink H, van Montfort P, van den Hoogen H, van den Bosch W, Grol R: **Continuous quality improvement in small general medical practices: the attitudes of general practitioners and other practice staff.** *Int J Qual Health Care* 2001, **13:**391-397.
6. Solberg LI, Brekke ML, Kottke TE, Steel RP: **Continuous quality improvement in primary care: what's happening?** *Med Care* 1998, **36:**625-635.
7. Solberg LI, Kottke TE, Brekke ML: **Will primary care clinics organize themselves to improve the delivery of preventive services? A randomized controlled trial.** *Prev Med* 1998, **27:**623-631.
8. Berwick D, Godfrey BA, Roessner J: *Curing health care: new strategies for quality improvement: a report on the National Demonstration Project on Quality Improvement in Health Care.* 1st edn. San Francisco: Jossey-Bass; 1990.
9. McLaughlin CP, Kaluzny AD: **Defining total quality management/continuous quality improvement.** In *Continuous quality improvement in health care: theory, implementation and applications.* third edition. Edited by McLaughlin CP, Kaluzny AD. Gaithersburg, Md.: Aspen Publishers Inc.; 1994: xii, 467
10. Solberg LI, Asche SE, Margolis KL, Whitebird RR: **Measuring an organization's ability to manage change: the change process capability questionnaire and its use for improving depression care.** *Am J Med Qual* 2008, **23:**193-200.
11. Solberg LI, Glasgow RE, Unutzer J, Jaeckels N, Oftedahl G, Beck A, Maciosek MV, Crain AL: **Partnership research: a practical trial design for evaluation of a natural experiment to improve depression care.** *Med Care* 2010, **48:**576-582.
12. Solberg LI: **Improving medical practice: a conceptual framework.** *Ann Fam Med* 2007, **5:**251-256.
13. Ingersoll GL, Kirsch JC, Merk SE, Lightfoot J: **Relationship of organizational culture and readiness for change to employee commitment to the organization.** *J Nurs Adm* 2000, **30:**11-20.

14. Solberg LI, Brekke ML, Fazio CJ, Fowles J, Jacobsen DN, Kottke TE, Mosser G, O'Connor PJ, Ohnsorg KA, Rolnick SJ: **Lessons from experienced guideline implementers: attend to many factors and use multiple strategies.** *Jt Comm J Qual Improv* 2000, **26:**171-188.
15. Alexander JA, Weiner BJ, Shortell SM, Baker LC: **Does quality improvement implementation affect hospital quality of care?** *Hosp Top* 2007, **85:**3-12.
16. Alexander JA, Weiner BJ, Griffith J: **Quality improvement and hospital financial performance.** *J Organ Behav* 2006, **27:**1003-1029.
17. Barsness ZI, Shortell SM, Gillies EFX, Hughes JL, O'Brien D: **The quality march. National survey profiles quality improvement activities.** *Hosp Health Netw* 1993, **67:**52-55.
18. Lucas JA, Avi-Itzhak T, Robinson JP, Morris CG, Koren MJ, Reinhard SC: **Continuous quality improvement as an innovation: which nursing facilities adopt it?** *Gerontologist* 2005, **45:**68-77.
19. Zinn JS, Brannon D, Weech R: **Quality improvement in nursing care facilities: extent, impetus, and impact.** *Am J Med Qual* 1997, **12:**51-61.
20. Zinn JS, Weech RJ, Brannon D: **Resource dependence and institutional elements in nursing home TQM adoption.** *Health Serv Res* 1998, **33:**261-273.
21. Weiner BJ, Alexander JA, Shortell SM: **Leadership for quality improvement in health care: empirical evidence on hospital boards, managers, and physicians.** *Med Care Res Rev* 1996, **53:**397-416.
22. Batalden P, Smith SD, Bovender JO, Hardison CD: **Quality improvement: the role and application of research methods.** *J Health Adm Educ* 1989, **7:**577-583.
23. Deming WE: *Out of the crisis.* Cambridge, Mass.: Cambridge University Press; 1986.
24. Juran JM: *Juran on planning for quality.* New York: Collier Macmillan; 1988.
25. Duckers MLA, Wagner C, Groenewegen PP: **Developing and testing an instrument to measure the presence of conditions for successful implementation of quality improvement collaboratives.** *BMC Health Serv Res* 2008, **8:**172.
26. Gustafson DH, Sainfort F, Eichler M, Adams L, Bisognano M, Steudel H: **Developing and testing a model to predict outcomes of organizational change.** *Health Serv Res* 2003, **38:**751-776.
27. Helfrich CD, Li YF, Sharp ND, Sales AE: **Organizational readiness to change assessment (ORCA): Development of an instrument based on the Promoting Action on Research in Health Services (PARiHS) framework.** *Implement Sci* 2009, **4:**38.
28. Lemieux-Charles L, Murray M, Baker G, Barnsley J, Tasa K, Ibrahim S: **The effects of quality improvement practices on team effectiveness: a mediational model.** *J Organ Behav* 2002, **23:**533-553.
29. Hackman J: **Total quality mangement: empirical, conceptual and practical issues.** *Adm Sci Q* 1995, **40:**309-329.
30. Whetton DA, Cameron KS: **Organizational effectiveness: old models and new constructs.** In *Organizational behavior : the state of the science.* Edited by Greenberg J. Hillsdale, N.J.: L. Erlbaum Associates; 1994: x, 312 p.

31. Meurer SJ, Rubio DM, Counte MA, Burroughs T: **Development of a healthcare quality improvement measurement tool: results of a content validity study.** *Hosp Top* 2002, **80:**7-13.

32. Schouten L, Grol R, Hulscher M: **Factors influencing success in quality improvement collaboratives: development and psychometric testing of an instrument.** *Implement Sci* 2010, **5:**84.

33. Kilo CM: **Improving care through collaboration.** *Pediatrics* 1999, **103:**384-393.

34. Øvretveit J, Bate P, Cleary P, Cretin S, Gustafson D, McInnes K, McLeod H, Molfenter T, Plsek P, Robert G, et al: **Quality collaboratives: lessons from research.** *Qual Saf Health Care* 2002, **11:**345-351.

35. Wilson T, Berwick DM, Cleary PD: **What do collaborative improvement projects do? Experience from seven countries.** *Jt Comm J Qual Saf* 2003, **29:**85-93.

36. Wilkens R, London M: **Relationships between climate, process, and performance in continuous quality improvement groups.** *J Vocat Behav* 2006, **69:**510-523.

37. Hackman JR, Wageman R: **A Theory of Team Coaching.** *Acad Manage Rev* 2005, **30:**269-287.

38. Bobiak SN, Zyzanski SJ, Ruhe MC, Carter CA, Ragan B, Flocke SA, Litaker D, Stange KC: **Measuring practice capacity for change: a tool for guiding quality improvement in primary care settings.** *Qual Manag Health Care* 2009, **18:**278-284.

39. Cohen D, McDaniel RR, Jr., Crabtree BF, Ruhe MC, Weyer SM, Tallia A, Miller WL, Goodwin MA, Nutting P, Solberg LI, et al: **A practice change model for quality improvement in primary care practice.** *J Healthc Manag* 2004, **49:**155-168; discussion 169-170.

40. Ruhe MC, Carter C, Litaker D, Stange KC: **A systematic approach to practice assessment and quality improvement intervention tailoring.** *Qual Manag Health Care* 2009, **18:**268-277.

41. Book S, Hellstrom A, Olsson J: **Perceptions of improvement work in Swedish health care: implications for improvement practices.** *Qual Manag Health Care* 2003, **12:**217-224.

42. Olsson J, Kammerlind P, Thor J, Elg M: **Surveying improvement activities in health care on a national level--the Swedish internal collaborative strategy and its challenges.** *Qual Manag Health Care* 2003, **12:**202-216.

43. Juran D: **Achieving sustained quantifiable results in an interdepartmental quality improvement project.** *Jt Comm J Qual Improv* 1994, **20:**105-119.

44. Gustafson DH, Hundt AS: **Findings of innovation research applied to quality management principles for health care.** *Health Care Manage Rev* 1995, **20:**16-33.

45. Shackelton R, Link C, Marceau L, McKinlay J: **Does the culture of a medical practice affect the clinical management of diabetes by primary care providers?** *J Health Serv Res Policy* 2009, **14:**96-103.

46. Kralewski J, Dowd BE, Kaissi A, Curoe A, Rockwood T: **Measuring the culture of medical group practices.** *Health Care Manage Rev* 2005, **30:**184-193.

47. Kralewski JE, Wingert TD, Barbouche MH: **Assessing the culture of medical group practices.** *Med Care* 1996, **34:**377-388.

48. Curoe A, Kralewski J, Kaissi A: **Assessing the cultures of medical group practices.** *J Am Board Fam Pract* 2003, **16:**394-398.

49. Kaissi A, Kralewski J, Dowd B, Heaton A: **The effect of the fit between organizational culture and structure on medication errors in medical group practices.** *Health Care Manage Rev* 2007, **32:**12-21.
50. Reynolds PD: **Organizational culture as related to industry, position and performance: A preliminary report.** *J Manage Stud* 1986, **23:**333-345.
51. Leykum L, Palmer R, Lanham H, Jordan M, McDaniel R, Noel P, Parchman M: **Reciprocal learning and chronic care model implementation in primary care: results from a new scale of learning in primary care settings.** *BMC Health Serv Res* 2011, **11:**44.
52. Landon BE, Wilson IB, McInnes K, Landrum MB, Hirschhorn L, Marsden PV, Gustafson D, Cleary PD: **Effects of a quality improvement collaborative on the outcome of care of patients with HIV infection: the EQHIV study.** *Ann Intern Med* 2004, **140:**887-896.
53. Marsden PV, Landon BE, Wilson IB, McInnes K, Hirschhorn LR, Ding L, Cleary PD: **The reliability of survey assessments of characteristics of medical clinics.** *Health Serv Res* 2006, **41:**265-283.
54. Deo S, McInnes K, Corbett CJ, Landon BE, Shapiro MF, Wilson IB, Cleary PD: **Associations between organizational characteristics and quality improvement activities of clinics participating in a quality improvement collaborative.** *Med Care* 2009, **47:**1026-1030.
55. Shortell SM, Jones RH, Rademaker AW, Gillies RR, Dranove DS, Hughes EF, Budetti PP, Reynolds KS, Huang CF: **Assessing the impact of total quality management and organizational culture on multiple outcomes of care for coronary artery bypass graft surgery patients.** *Med Care* 2000, **38:**207-217.
56. Sarin S, McDermott C: **The Effect of Team Leader Characteristics on Learning, Knowledge Application, and Performance of Cross-Functional New Product Development Teams.** *Decision Sci* 2003, **34:**707-739.
57. Edmondson A: **Psychological safety and learning behavior in work teams.** *Adm Sci Q* 1999, **44:**350-383.
58. Orzano AJ, Ohman-Strickland PA, Patel M: **What can family medicine practices do to facilitate knowledge management?** *Health Care Manage Rev* 2008, **33:**216-224.
59. Ohman-Strickland PA, John Orzano A, Nutting PA, Perry Dickinson W, Scott-Cawiezell J, Hahn K, Gibel M, Crabtree BF: **Measuring organizational attributes of primary care practices: development of a new instrument.** *Health Serv Res* 2007, **42:**1257-1273.
60. Hung DY, Rundall TG, Crabtree BF, Tallia AF, Cohen DJ, Halpin HA: **Influence of Primary Care Practice and Provider Attributes on Preventive Service Delivery.** *Am J Prev Med* 2006, **30:**413-422.
61. Hudson SV, Ohman-Strickland P, Ferrante JM, Lu-Yao G, Orzano AJ, Crabtree BF: **Prostate-specific antigen testing among the elderly in community-based family medicine practices.** *J Am Board Fam Med* 2009, **22:**257-265.
62. Ohman-Strickland PA, Orzano AJ, Hudson SV, Solberg LI, Diciccio-Bloom B, O'Malley D, Tallia AF, Balasubramanian BA, Crabtree BF: **Quality of diabetes care in family medicine practices: Influence of nurse-practitioners and physician's assistants.** *Ann Fam Med* 2008, **6:**14-22.
63. Orzano AJ, McInerney CR, Scharf D, Tallia AF, Crabtree BF: **A knowledge management model: Implications for enhancing quality in health care.** *J Am Soc Inf Sci Technol* 2008, **59:**489-505.

64. Parker VA, Wubbenhorst WH, Young GJ, Desai KR, Charns MP: **Implementing quality improvement in hospitals: the role of leadership and culture.** *Am J Med Qual* 1999, **14:**64-69.

65. Goldzweig CL, Parkerton PH, Washington DL, Lanto AB, Yano EM: **Primary Care Practice and Facility Quality Orientation: Influence on Breast and Cervical Cancer Screening Rates.** *Am J Manag Care* 2004, **10:**265-272.

66. Anaya HD, Yano EM, Asch SM: **Early adoption of human immunodeficiency virus quality improvement in Veterans Affairs medical centers: use of organizational surveys to measure readiness to change and adapt interventions to local priorities.** *Am J Med Qual* 2004, **19:**137-144.

67. Charbonneau A, Parker V, Meterko M, Rosen AK, Kader B, Owen RR, Ash AS, Whittle J, Berlowitz DR: **The relationship of system-level quality improvement with quality of depression care.** *Am J Manag Care* 2004, **10:**846-851.

68. National Institute of Standards and Technology: **Baldrige National Quality Program: Health Care Criteria for Performance Excellence**. Galthersberg 2005.

69. Rushmer RK, Kelly D, Lough M, Wilkinson JE, Greig GJ, Davies HT: **The Learning Practice Inventory: diagnosing and developing Learning Practices in the UK.** *J Eval Clin Pract* 2007, **13:**206-211.

70. Kelly DR, Lough M, Rushmer R, Wilkinson JE, Greig G, Davies HTO: **Delivering feedback on learning organization characteristics--using a Learning Practice Inventory.** *J Eval Clin Pract* 2007, **13:**734-740.

71. Kelly DR, Crossley J, Lough M, Rushmer R, Greig GJ, Davies HT: **Diagnosing a learning practice: the validity and reliability of a learning practice inventory.** *Qual Saf Health Care* 2011, **20:**209-215.

72. Rushmer R, Kelly D, Lough M, Wilkinson JE, Davies HT: **Introducing the Learning Practice--I. The characteristics of Learning Organizations in Primary Care.** *J Eval Clin Pract* 2004, **10:**375-386.

73. Rushmer R, Kelly D, Lough M, Wilkinson JE, Davies HT: **Introducing the Learning Practice--II. Becoming a Learning Practice.** *J Eval Clin Pract* 2004, **10:**387-398.

74. Rushmer R, Kelly D, Lough M, Wilkinson JE, Davies HT: **Introducing the Learning Practice--III. Leadership, empowerment, protected time and reflective practice as core contextual conditions.** *J Eval Clin Pract* 2004, **10:**399-405.

75. Hall CB, Tennen H, Wakefield DB, Brazil K, Cloutier MM: **Organizational assessment in paediatric primary care: development and initial validation of the primary care organizational questionnaire.** *Health Serv Manage Res* 2006, **19:**207-214.

76. Shortell SM, Rousseau DM, Gillies RR, Devers KJ, Simons TL: **Organizational assessment in intensive care units (ICUs): construct development, reliability, and validity of the ICU nurse-physician questionnaire.** *Med Care* 1991, **29:**709-726.

77. Brazil K, Wakefield DB, Cloutier MM, Tennen H, Hall CB: **Organizational culture predicts job satisfaction and perceived clinical effectiveness in pediatric primary care practices.** *Health Care Manage Rev* 2010, **35:**365-371.

78. Cloutier MM, Wakefield DB, Tsimikas J, Hall CB, Tennen H, Brazil K: **Organizational attributes of practices successful at a disease management program.** *J Pediatr* 2009, **154:**290-295.

79. Stevenson K, Baker R: **Investigating organisational culture in primary care.** *Qual Prim Care* 2005, **13:**191-200.

80. Stevenson K: **Are your practices resistant to changing their clinical culture?** *Primary Care Report* 2000, **2:**19-20.

81. Donaldson L: **A First Class Service: quality in the new NHS**. Department of Health London 1999.

82. Sylvester S: **Measuring the learning practice: diagnosing the culture in general practice.** *Qual Prim Care* 2003, **11:**29-40.

83. Dobson C: **Can the key characteristics of a learning organisation be found in our general practice?** *Educ Prim Care* 2008, **19:**74-79.

84. Kinston W: *Strengthening the management culture: phasing the transformation of organizations.* London: SIGMA Centre; 1994.

85. Mintzberg H: *The structuring of organizations : a synthesis of the research.* Englewood Cliffs, N.J.: Prentice-Hall; 1979.

86. Morgan G: *Images of organization.* 2nd edn. Thousand Oaks, California: Sage Publications; 1997.

87. Duckers M, Spreeuwenberg P, Wagner C, Groenewegen P: **Exploring the black box of quality improvement collaboratives: modelling relations between conditions, applied changes and outcomes.** *Implement Sci* 2009, **4:**74.

88. Vos L, Duckers M, Wagner C, van Merode G: **Applying the quality improvement collaborative method to process redesign: a multiple case study.** *Implement Sci* 2010, **5:**19.

89. Molfenter T, Gustafson D, Kilo C, Bhattacharya A, Olsson J: **Prospective evaluation of a Bayesian model to predict organizational change.** *Health Care Manage Rev* 2005, **30:**270-279.

90. Hall LW, Headrick LA, Cox KR, Deane K, Gay JW, Brandt J: **Linking health professional learners and health care workers on action-based improvement teams.** *Qual Manag Health Care* 2009, **18:**194-201.

91. Hagedorn HJ, Heideman PW: **The relationship between baseline Organizational Readiness to Change Assessment subscale scores and implementation of hepatitis prevention services in substance use disorders treatment clinics: A case study.** *Implement Sci* 2010, **5**.

92. Kitson A, Harvey G, McCormack B: **Enabling the implementation of evidence based practice: a conceptual framework.** *Qual Health Care* 1998, **7:**149-158.

93. Lageson C: **Brief report: reliability and validity of the TQManager feedback instrument.** *J Healthc Qual* 2006, **28:**45-49.

94. Juran JM: *Juran on leadership for quality : an executive handbook.* New York: Free Press; 1989.

95. Crosby PB: *Quality is free.* New York: New American Library; 1979.

96. Meurer SJ, Counte MA, Rubio DM, Arrington B: **Measuring a hospital's ability to improve.** *Am J Med Qual* 2004, **19:**214-222.

97. Lin MK, Marsteller JA, Shortell SM, Mendel P, Pearson M, Rosen M, Wu SY: **Motivation to change chronic illness care: results from a national evaluation of quality improvement collaboratives.** *Health Care Manage Rev* 2005, **30:**139-156.

98. Cretin S, Shortell SM, Keeler EB: **An evaluation of collaborative interventions to improve chronic illness care. Framework and study design.** *Eval Rev* 2004, **28:**28-51.

99. Shortell SM, Marsteller JA, Lin M, Pearson ML, Wu SY, Mendel P, Cretin S, Rosen M: **The role of perceived team effectiveness in improving chronic illness care.** *Med Care* 2004, **42:**1040-1048.

100. Zammuto RF, Krakower JY: **Quantitative and qualitative studies of organizational culture.** *Res Organ Chan Dev* 1991, **5:**83-114.

101. Quinn R, Spreitzer G: **The psychometrics of the Competing Values Culture Instrument and an analysis of the impact of organizational culture on quality of life.** In *Research in organizational change and development. Volume* 5. Edited by Woodman R, Pasmore W. Greenwich, CT: JAI Press; 1991: 115-142

102. Helfrich CD, Li YF, Mohr DC, Meterko M, Sales AE: **Assessing an organizational culture instrument based on the Competing Values Framework: exploratory and confirmatory factor analyses.** *Implement Sci* 2007, **2:**13.

103. Hann M, Bower P, Campbell S, Marshall M, Reeves D: **The association between culture, climate and quality of care in primary health care teams.** *Fam Pract* 2007, **24:**323-329.

104. Bosch M, Dijkstra R, Wensing M, van der Weijden T, Grol R: **Organizational culture, team climate and diabetes care in small office-based practices.** *BMC Health Serv Res* 2008, **8:**180.

105. Quinn RE, Rohrbaugh J: **A Competing Values Approach to Organizational Effectiveness.** *Public Product Rev* 1981, **5:**122-140.

106. Quinn RE, Rohrbaugh J: **A spatial model of effectiveness criteria: Towards a competing values approach to organizational analysis.** *Manage Sci* 1983, **29:**363-377.

107. Armenakis AA, Bernerth JB, Pitts JP, Walker HJ: **Organizational Change Recipients' Beliefs Scale: Development of an Assessment Instrument.** *J Appl Behav Sci* 2007, **43:**481-505.

108. Holt DT, Armenakis AA, Field HS, Harris SG: **Readiness for Organizational Change.** *J Appl Behav Sci* 2007, **43:**232-255.

109. Calomeni CA, Solberg LI, Conn SA: **Nurses on quality improvement teams: how do they benefit?** *J Nurs Care Qual* 1999, **13:**75-90.

110. Solberg LI, Kottke TE, Brekke ML, Magnan S, Davidson G, Calomeni CA, Conn SA, Amundson GM, Nelson AF: **Failure of a continuous quality improvement intervention to increase the delivery of preventive services. A randomized trial.** *Eff Clin Pract* 2000, **3:**105-115.

111. Georgopoulos BS, Mahoney GM, Jones NW, Jr.: **A path-goal approach to productivity.** *J Appl Psychol* 1957, **Vol.41:**pp.

112. Vroom VH: *Work and motivation.* New York: Wiley; 1964.

113. Solberg LI, Brekke ML, Kottke TE, Steel RP: **Continuous quality improvement in primary care: what's happening?[see comment].** *Med Care* 1998, **36:**625-635.

114. Cunningham CE, Woodward CA, Shannon HS, MacIntosh J, Lendrum B, Rosenbloom D, Brown J: **Readiness for organizational change: A longitudinal study of workplace, psychological and behavioural correlates.** *J Occup Organ Psychol* 2002, **75:**377-392.

115. Christl B, Harris MF, Jayasinghe UW, Proudfoot J, Taggart J, Tan J: **Readiness for organisational change among general practice staff.** *Qual Saf Health Care* 2010.

116. Prochaska JO, Hedding CA, Evers K: **The transtheoretical model of change.** In *Health behavior and education: theory, research and practice.* Edited by K. Glanz, Lewis FM, Rimer BK. San Francisco: Jossey Bass; 1997: 60-84

117. Prochaska JO, Velicer WF, Rossi S, Goldstein MG, Marcus BH, Rakowski V, Fiore C, Harlow Ll, Redding CA, Rosenbloom D, Rossi SR: **Stages of change and decisional balance for 12 problem behaviors.** *Health Psychol* 1994, **13:**39-46.

118. Herscovitch L, Meyer JP: **Commitment to organizational change: extension of a three-component model.** *J Appl Psychol* 2002, **87:**474-487.

119. Meyer JP, Srinivas ES, Lal JB, Topolnytsky L: **Employee commitment and support for an organizational change: Test of the three-component model in two cultures.** *J Occup Organ Psychol* 2007, **80:**185-211.

120. Meyer JP, Herscovitch L: **Commitment in the workplace: Toward a general model.** *Hum Resour Manage R* 2001, **11:**299-326.

121. Irvine D: **The development of measures of organizational citizenship behaviour and changes in job behaviours related to quality management in health care.** *Health Serv Manage Res* 1995, **8:**143-161.

122. Irvine DM, Leatt P, Evans MG, Baker GR: **The behavioural outcomes of quality improvement teams: the role of team success and team identification.** *Health Serv Manage Res* 2000, **13:**78-89.

123. Irvine D, Leatt P, Evans MG, Baker RG: **Measurement of staff empowerment within health service organizations.** *J Nurs Meas* 1999, **7:**79-96.

124. Kuokkanen L, Suominen T, Rankinen S, Kukkurainen M-L, Savikko N, Doran D: **Organizational change and work-related empowerment.** *J Nurs Manag* 2007, **15:**500-507.

125. Rankinen S, Suominen T, Kuokkanen L, Kukkurainen ML, Doran D: **Work empowerment in multidisciplinary teams during organizational change.** *Int J Nurs Pract* 2009, **15:**403-416.

126. Conger JA, Kanungo RN: **The empowerment process: Integrating theory and practice.** *Acad Manage Rev* 1988, **13:**471-482.

127. Coyle-Shapiro JAM, Morrow PC: **The role of individual differences in employee adoption of TQM orientation.** *J Vocat Behav* 2003, **62:**320-340.

128. Hanpachern C, Hanpachern C, Morgan GA, Griego OV: **An Extension of the Theory of Margin: A Framework for Assessing Readiness for Change.** *Hum Resour Dev Q* 1998, **9:**339-350.

129. McNabb DE, Sepic FT: **Culture, Climate, and Total Quality Management: Measuring Readiness for Change.** *Public Product Manage Rev* 1995, **18:**369-385.

130. Pare G, Sicotte C, Poba-Nzaou P, Balouzakis G: **Clinicians' perceptions of organizational readiness for change in the context of clinical information system projects: insights from two cross-sectional surveys.** *Implement Sci* 2011, **6:**15.

131. Holt DT, Armenakis AA, Harris SG, Feild HS, William APaRWW: **Toward a Comprehensive Definition of Readiness for Change: A Review of Research and Instrumentation.** In *Research in Organizational Change and Development. Volume* 16: JAI; 2007: 289-336
