# Supplementary material for: Measuring organizational and individual factors thought to influence the success of quality improvement in primary care: a systematic review of instruments
Source: Implement Sci. 2012 Dec 17;7:121. doi: 10.1186/1748-5908-7-121 (PMC3573896; doi:10.1186/1748-5908-7-121)
Supplement: Additional file 6 — Tables summarising the development and measurement properties of instruments included in Stage 4 of the review (Tables S11–S13). [file 1748-5908-7-121-S6.pdf]

Additional file 6 - Tables summarising the development and measurement properties of instruments included in Stage 4 of the review

Table 11: Summary of instrument development and assessments of measurement properties - CQI use and implementation

**Instrument**<br>(name, index, references for main reports)	**Development & content validity**<br>(item generation; content validity assessments)	**Construct validity**	**Reliability**	**Other assessments**<br>(eg feasibility, acceptability, interpretability)
Primary care settings				
Quality improvement methods<br><br>Apekey, 2011a [1]	Methods of item generation not reported.<br><br>Content validity: no independent assessments reported.<br><br>Administered to QI leads in general practices in the UK (RR 63/102, 62%).	Hypothesis testing: Association between adoption of quality improvement methods (scale scores) and leadership behaviour and culture of innovation. Results not reported.<br><br>Structure: not assessed	Internal consistency: investigators reported that Cronbach's alphas was calculated for the scale, however results were not reported.<br><br>Stability over time: not assessed	No other assessments reported.
Quality improvement activities<br><br>Engels, 2006 [2]	Items match the steps in the QI cycle component of the intervention tested in this study. Methods of item generation not reported.<br><br>Content validity: no independent assessments reported.<br><br>Administered to primary care practices participating in a RT of CQI (pre- and post-intervention data for 45/49 practices, 91%)	Hypothesis testing: Intervention and control groups were expected to differ on scores, with a higher % of intervention group practice using QI cycle steps. Hypothesis supported. No other hypotheses tested.<br><br>Structure: not assessed	Not assessed.	No other assessments reported.
Attitudes toward CQI model<br><br>Geboers, 2001a [3]	Items match the components of the QI model implemented in this study. Methods of item generation not reported.<br><br>Content validity: no independent assessments reported.<br><br>Administered to staff in general practices implementing CQI (RR 92/118, 78%)	No assessments of construct validity identified.	Not assessed.	No other assessments reported.
Process improvement progress (PIP)<br><br>Solberg, 1998a [4-5]	Items were based on "descriptions of recommended process improvement tasks" (p627) from selected literature. Method of item generation not reported.<br><br>Content validity: The instrument was pre-tested in two clinics and unspecified revisions made. No other assessments of content validity reported.<br><br>Administered to QI team members participating in a RT of CQI in primary care (RR 163/175 representing 22 intervention group teams, 93%)	Hypothesis testing: Correlation between number of process improvement tasks and perceived process improvement. Hypotheses were supported.<br><br>Structure: not assessed	Not assessed.	No other assessments reported.

**Instrument** (name, index, references for main reports)	**Development & content validity** (item generation; content validity assessments)	**Construct validity**	**Reliability**	**Other assessments** (eg feasibility, acceptability, interpretability)
Change process capability questionnaire (CPCQ)<br><br>Solberg, 2008 [6]	Items were based on data from an earlier study aiming to identify contextual factors important for successful implementation of clinical practice guidelines and strategies to address the factors. Factors and strategies were derived from selected literature and interviews with clinical leaders experienced in QI, and then ranked in a Delphi process. International experts commented on the ranked list [7]. Items were developed from this data.<br><br>Content validity: no independent assessments reported.<br><br>Initial testing with QI coordinators in medical group practices (RR 41/41, 100%)	Hypothesis testing: Association between CPCQ scores (total and subscale) and i) priority for improving depression care; ii) systems for delivering depression care (overall score; scores on subscales). Some hypotheses supported.<br><br>Structure: Items purporting to measure organisational factors were grouped (and summed) to form three scales. Grouping was based on domains of a theoretical model of change [8], however the structure of these scales was not tested empirically (i.e. using factor analysis).	Internal consistency: Cronbach's alpha for all scales and subscales reported. Two subscales <0.70.<br><br>Stability over time: not assessed.	No other assessments reported.
Other healthcare settings				
CQI/TQM adoption indicators<br><br>Barsness, 1993 [9]	Methods of item generation not reported.<br><br>Content validity: no independent assessments reported.<br><br>Administered to CEOs and person in charge of QI in national samples of hospitals in 1993 (RR 3303/5492, 60%) [9-10] and 1997 (RR 2,350/6150, 38%; 1748 provided data for analysis) [11-12]. Other administrations to hospital CEOs [13] and aged care nursing facility administrators (RR 241/615, 39.2%) [14-15].	Hypothesis testing (examples only): no testing in primary care. Association between adoption of a CQI/TQM approach and i) degree of QI implementation (Baldrige measures) and ii) patient, financial and HR outcomes. Hypotheses not supported [13]. Association between physician involvement in governance and CQI/TQM adoption. Mixed support for hypotheses [12].<br><br>Structure: Empirical support for a single scale (exploratory factor analysis) from a single study [12], however methods and results of analysis not reported.	Internal consistency: Cronbach's alpha for single scale 0.84 [12]. No other reports.<br><br>Stability over time: not assessed.	Alexander and colleagues report use of a Likert scaled measure [11-12]. Dichotomous scales appear to have been used in all other studies, however this is not explicitly stated in all reports.<br><br>Four studies used this instrument to classify organisations as CQI/TQM adopters or non-adopters, reporting only descriptive data on attributes of each group [9, 16-18].
Supportive conditions for QI collaborative (QIC) implementation	See Duckers, 2008 [19] under 'organisational context' domain – Table 9			
Organizational Change Manager (OCM)	See Gustafson, 2003 [20] under 'organisational context' domain – Table 9			
Organizational readiness to change assessment (ORCA)	See Helfrich, 2009 [21] under 'organisational context' domain – Table 9			

**Instrument** (name, index, references for main reports)	**Development & content validity** (item generation; content validity assessments)	**Construct validity**	**Reliability**	**Other assessments** (eg feasibility, acceptability, interpretability)
QI practices index<br><br>Lemieux-Charles, 2002 [22]	Items were based on selected literature related to QI. Methods of item generation not reported.<br><br>Content validity: no independent assessments reported.<br><br>Initial testing in QI teams from acute care hospitals (RR 506/732 team members, 69%)	Hypothesis testing: i) Direct effects of QI practices on self-rated and externally rated team effectiveness; ii) QI practices as a mediator of the effect of context on performance; iii) team process as a mediator of effect of QI practices on team effectiveness. Mixed support for hypotheses.<br><br>Structure: not assessed	Internal consistency: Cronbach's alpha for single scale was 0.70<br><br>Stability over time: not assessed	No other assessments.
Continuous quality improvement questionnaire	See Meurer, 2002 [23] under 'organisational context' domain – Table 9			
Factors influencing success in a QIC<br><br>Schouten, 2010 [24]	Items derived from a systematic review of QIC theory from which determinants of QIC success were identified. Overlapping items were combined, and vague, ambiguous or redundant items were removed.<br><br>Content validity: content experts reviewed items for "readability, comprehensibility, ease of response, content validity" (p2). Items were accepted or deleted based on expert agreement.<br><br>Initial testing in a sample of QIC participants (RR 44/46 teams, 95%)	Hypothesis testing: Correlation between scales. All hypotheses supported.<br><br>Structure: Four theoretical dimensions were predicted. Two were empirically supported. A third factor included items from the remaining two dimensions (factors identified by principal component analysis).	Internal consistency: Cronbach's alpha for the three scales ranged from 0.85 to 0.89.<br><br>Stability over time: not assessed.	
CQI process facilitation<br><br>Wilkens, 2006 [25]	The authors describe the role of facilitation and mechanism by which it influences team outcomes. Methods of item generation not reported.<br><br>Content validity: no independent assessments reported.<br><br>Initial testing in a sample of CQI team members in a university hospital (RR 49/76 members, 64% from 8 teams)	Hypothesis testing: Association with i) team climate scales (self-disclosure; psychological safety; learning orientation), ii) measures of team process (feedback giving and seeking; task and relationship conflict) and iii) perceived team effectiveness (creativity of outcomes; perceived effectiveness). Mixed support for hypotheses.<br><br>Structure: not assessed	Internal consistency: Cronbach's alpha for single scale was 0.97<br><br>Stability over time: not assessed	No other assessments

Table 12: Summary of instrument development and assessment of measurement properties - organisational context

**Instrument** (name, index, references for main reports)	**Development & content validity** (item generation; content validity assessments)	**Construct validity**	**Reliability**	**Other assessments** (eg feasibility, acceptability, interpretability)
Primary care settings				
Practice capacity for change<br><br>Bobiak, 2009 [26]	Items were written to operationalise a practice change model and factors thought to influence change capacity (structure, climate, culture). Inter-item correlations and Rasch analysis were used to identify redundant items and select those with greatest discriminative ability and best fit with the model.<br><br>Content validity: no independent assessments reported.<br><br>Data from a single researcher's rating of 15 practices based on observation and interviews (i.e. not self report data).	Hypothesis testing: Association between scale scores and independent rating of capacity for change (single item, 10 point scale) by three researchers. Both scores based on researcher assessment of qualitative data (field notes and interviews). Rasch analysis used to assess scale's ability to discriminate between practices.<br><br>Structure: Rasch analysis identified a single dominant factor and was used to assess item fit with model.	Internal consistency: Cronbach's alpha for single scale was 0.94.<br><br>Stability over time: not assessed	No other assessments.<br><br>This instrument requires researcher scoring of items based on field observation and interviews.
Perceptions of improvement work<br><br>Book, 2003 [27-28]	Items based on expert views, change management and diffusion of innovation literature. Methods of item generation not reported.<br><br>Content validity: Drafts were reviewed and pre-tested by a small number of health services managers, and revised accordingly. Details not reported.<br><br>Instrument was administered to managers of all primary care centres and hospital departments (RR 1062/2313, 45.9%).	Hypothesis testing: Association between scale scores and a single item, self-report measure of perceived results from QI (outcome measure). Hypotheses not supported.<br><br>Structure: Items were written to measure five attributes based on diffusion of innovation theory. Three factors were identified by principal components analysis.	Internal consistency: not reported<br><br>Stability over time: not assessed	No other assessments
Obstacles to the use of CQI<br><br>Geboers 2001b [3]	Items based on selected literature. Methods of item generation not reported.<br><br>Content validity: no independent assessments reported.<br><br>Administered to staff in general practices implementing CQI (RR 92/118, 78%)	No assessment of construct validity.	No assessment of reliability.	Level of analysis: Homogeneity of responses was assessed to confirm that within practice agreement was greater than between.

**Instrument**<br>(name, index, references for main reports)	**Development & content validity**<br>(item generation; content validity assessments)	**Construct validity**	**Reliability**	**Other assessments**<br>(eg feasibility, acceptability, interpretability)
Medical Group Practice Culture<br><br>Kralewski, 2005 [29-31]	Medical directors from 30 group practices each wrote three statements describing their practice culture. Items were grouped by dimensions of a theoretical framework and items judged as redundant by investigators were removed.<br><br>Content validity: Item refinement involved several administrations of items to physicians in group practices (RR 69% from 259 physicians, then 45% from 1223). Comments on item appropriateness and missing items were elicited. Items that did not differentiate between practices (based on descriptive statistics) or load onto factors (principal components analysis) were removed. New items were added.<br><br>A forty seven item instrument was administered to physicians in group practices in multiple studies (RR for assessments reported here 547/1223 physicians, 45% from 148/191 medical groups)	Hypothesis testing: Association between individual dimensions of culture (scale scores), and practice size and complexity, specifically: i) small versus large practices, ii) rural versus urban, iii) single- versus multi-specialty and iv) physician-owned versus hospital or health plan owned. Most hypotheses supported.<br><br>Structure: Twelve dimensions were predicted from the initial theoretical model. Nine were supported empirically (principal components analysis).	Internal consistency: Cronbach's alpha for nine scales ranged from 0.8 to 0.86.<br><br>Stability over time: not assessed	No other assessments.
Reciprocal learning scale<br><br>Leykum, 2011 [32]	Items were written to operationalise six themes identified from organisational learning literature. Methods of item generation not reported.<br><br>Content validity: 22 items were administered to primary care staff and clinicians (101 staff, 5 clinics). Feedback was elicited to refine item wording.<br><br>Revised items were administered to staff and clinicians in primary care clinics participating in a randomised trial (RR not reported: 296 respondents from 40 clinics).	Hypothesis testing: Association between scale scores and chronic care model implementation. Hypothesis was supported.<br><br>Structure: Three factors were identified (principal components analysis). Items loading on two of the factors were dropped because scale reliability scores were below the pre-specified threshold (>0.7). Items loading on the remaining factor were assessed for conceptual similarity; five of eight items were retained.	Internal consistency: Cronbach's alpha for single scale was 0.79.<br><br>Stability over time: not assessed	No other assessments.
Openness to QI and climate for QI participation<br><br>Marsden, 2006 [33-34]	Methods of item generation not reported.<br><br>Content validity: no independent assessments reported.<br><br>Instrument was administered to administrators and physicians pre- and post- participation in a QI collaborative (RR 41/62 clinics, 66% provided complete data used for hypothesis testing, factor analysis, and internal consistency. Assessments of organisation level reliability were performed on a larger sample RR 89%, 379 respondents from 49 clinics)	Hypothesis testing: Association between scale scores and number, breadth, depth and comprehensiveness of chronic care model intervention implemented. Most hypotheses were supported.<br><br>Structure: Items were written to measure two constructs. Two dimensions were identified (exploratory factor analysis). Not all items loaded onto the expected factors; scales were relabelled to reflect item loading.	Internal consistency: Cronbach's alpha for two scales was 0.87 and 0.70 respectively.<br><br>Stability over time: not assessed<br><br>The effect on reliability of increasing i) the number of informants (respondents) and ii) number of items used to measure organisational level factors was investigated.	Differences in scale scores from different types of informants (managers versus physicians) were assessed, to investigate potential effects on the measurement of group or organisational level factors.

**Instrument**<br>(name, index, references for main reports)	**Development & content validity**<br>(item generation; content validity assessments)	**Construct validity**	**Reliability**	**Other assessments**<br>(eg feasibility, acceptability, interpretability)
Survey of Organizational Attributes for Primary Care (SOAPC)<br><br>Ohman-Strickland, 2007 [35-36]	Items derived from existing instruments designed to measure core concepts from the model for practice change.<br><br>Content validity: expert panel workshop held to review core concepts, sort items accordingly, remove duplicates and, if necessary, adapt wording for primary care. A revised version of the instrument was later developed to include measures of knowledge management [36].<br><br>Initial testing with staff and clinicians in practices participating in a randomised trial (RR 640/1100, representing 58% of all staff from 51/61 practices). Results from a subset of 13 practices were reported in a second paper [36].	Hypothesis testing: Association between individual scale scores [35]; scale scores and knowledge management (revised scales) [36]; scale scores and preventive service delivery [37]. Most hypotheses supported.<br><br>Structure: Four factors were identified (exploratory factor analysis). Items that cross-loaded or failed to load onto factors were eliminated, taking into account between versus within practice variability, correlation and conceptual similarity with other items in the factor.	Internal consistency: Cronbach's alpha for the four scales ranged from 0.73 to 0.88.<br><br>Stability over time: not assessed	Reported completion time: 15-20 minutes.<br><br>Multiple studies from the ULTRA trial report use of SOAPC (also called the Practice Staff Questionnaire (PSQ)), primarily contributing evidence related to construct validity (e.g. [38-39].
Climate for improvement<br><br>Olsson, 2003 [28]	Items based on expert views, change management and diffusion of innovation literature. Method of item generation not reported.<br><br>Content validity: Drafts were reviewed and pre-tested by a small number of health services managers, and revised accordingly. Details not reported.<br><br>Instrument was administered to managers of all primary care centres and hospital departments (RR 1062/2313, 45.9%).	Hypothesis testing: none reported<br><br>Structure: not assessed	No assessment of reliability	Data presented: respondent ranking of extent to which statements relating to climate for improvement reflect their organisation.
Degree of QI implementation<br><br>Parker, 1999 [40]	Items and dimensions based on Malcolm Baldrige Award criteria. Methods of item generation not reported.<br><br>Content validity: no independent assessments reported.<br><br>Instrument was administered as part of a staff survey to national samples of clinical, managerial and other staff working in Veterans Health Administration in 1997, 1998 and 2000 (RR 1997: 12399/18292 employees and managers, 68%) (multiple publications).	Hypothesis testing: Association between QI implementation and i) leadership and ii) culture in hospitals [40]; QI and depression care adequacy in hospitals [41]; and QI and cancer screening in primary care [42]. Most hypotheses supported.<br><br>Structure: no assessment reported	Internal consistency: Cronbach's alpha for five subscales ranged from 0.89 to 0.92 [41].<br><br>Stability over time: assessment not reported.	The extent of testing is unclear from published reports. Parker et al [40] state "reliability and validity of this instrument were both supported by previous testing" (p65). In a later paper, Charbonneau et al [41] reported that to their knowledge "neither construct nor criterion validity had been tested" (p848).

**Instrument**<br>(name, index, references for main reports)	**Development & content validity**<br>(item generation; content validity assessments)	**Construct validity**	**Reliability**	**Other assessments**<br>(eg feasibility, acceptability, interpretability)
Learning Practice Inventory (LPI)<br><br>Rushmer, 2007 [43-45]	Domains, and items written to measure each domain, were derived from themes identified from literature review. Item wording, order, context and response format were designed to minimise socially desirable responses.<br><br>Content validity: Items were pre-tested with general practice staff and clinicians to obtain feedback on clarity and ease of completion. Experts rated items on relevance and clarity in a nominal group process (alternating survey rounds and consensus meetings). Items were revised, added or removed accordingly.<br><br>Initial testing with staff and clinicians from a purposive sample of 10 general practices (10 staff recruited per practice; RR not reported)	Hypothesis testing: none reported.<br><br>Structure: Eight distinct domains (dimensions) were expected based on the investigators' grouping of conceptually similar items. Many items did not load on factors as expected or cross-loaded on multiple factors.	Estimates of variance attributable to individual and practice level were calculated through variance component modelling. Resulting generalisability coefficients indicated that reliability was acceptable with eight respondents per practice (0.69), but improved with 16 respondents (0.8).	Administration of the self-report instrument is intended to be overseen by a trained facilitator.
Change process capability questionnaire (CPCQ)	See Solberg, 2008 under 'CQI use' domain – Table 8			
Organizational assessment in ICU measure<br><br>Shortell, 1991 [46-47]	Constructs and dimensions based on literature review of practices and processes key to organisational effectiveness. Items were written for each identified factor. Some items were derived from existing scales. Items from four scales in the original instrument were later adapted for primary care [47].<br><br>Content validity: Instrument pilot-tested with ICU nurses and physicians (n=187). Based on findings, a second profession-specific version was written and some items revised for clarity. No independent assessments of content reported.<br><br>Initial testing with ICU staff (1418 returned, RR 73%) [46-47]. Primary care version administered to all clinicians and staff in practices participating in randomised trial (RR 353/420, 84% from all 36 practices) [47].	Hypothesis testing: Association between scale scores and outcomes: i) perceived technical quality of care, ii) unit ability to meet family member needs, and iii) nursing turnover. Association between scale scores. Most hypotheses supported. Multiple hypotheses subsequently tested in primary care sample [48-49].<br><br>Structure: Subscales for each of the constructs were identified using exploratory factor analysis. Most theoretically predicted scales in the primary care version were empirically supported (principal components analysis).	Internal consistency: Cronbach's alphas for scale scores ranged from 0.64 to 0.88. Primary care version, 0.70 to 0.93.	Feasibility: completion time for short form of ICU version approximately 20 minutes.

**Instrument** (name, index, references for main reports)	**Development & content validity** (item generation; content validity assessments)	**Construct validity**	**Reliability**	**Other assessments** (eg feasibility, acceptability, interpretability)
Practice Culture Questionnaire (PCQ)<br><br>Stevenson, 2005 [50-51]	Items based on a theoretical framework for an organisational culture resistant to clinical governance. Phrasing used for items was intended to be consistent with that used by general practice staff in an earlier (unrelated) qualitative study on QI.<br><br>Content validity: no independent assessments reported.<br><br>Instrument was administered for pilot testing to all team members from 17 general practices (RR 151/213, 71% of practice members).	Hypothesis testing: none reported.<br><br>Structure: not assessed	No assessment of reliability.	Acceptability: Based on median RR per practice (88%) and a RR ≤50% in 5/17 practices, the instrument was judged to be acceptable to respondents.<br><br>Based on visual assessment of median practice scores, the investigators concluded that the instrument differentiated between practices.
Learning organization characteristics questionnaire<br><br>Sylvester, 2003 [52]	Dimensions and items were derived from a review of management literature. Methods of item generation not reported.<br><br>Content validity: no independent assessments reported.<br><br>Reliability assessment based on administration to general practice staff in 5 practices (RR 23/25, 92%). Subsequent administration to general practices with ≥5 staff in a UK primary care trust. (RR 248/290; 85.5% of staff from 15/16 practices).	Hypothesis testing: none reported.<br><br>Structure: not assessed<br><br>Descriptive statistics reported for second administration, but no analyses.	Internal consistency: kappa statistics calculated to assess agreement between scores on a single pair of items in each subscale. Strength of agreement was rated as moderate to good for all pairings.<br><br>Intra-rater reliability: kappa statistics calculated to assess agreement between scores on repeated administrations of the instrument to the same respondents (10-12 weeks apart).	Acceptability: 13/15 practice had a RR >80% and overall response rate was 85.5%, hence the instrument was judged as acceptable.
Other healthcare settings				
Supportive conditions for QI collaborative (QIC) implementation<br><br>Duckers, 2008 [19, 53]	Items based on selected literature on each of the three dimensions (organisational support, team organisation, external change agent support). Methods of item generation not reported.<br><br>Content validity: Assessed by nine content experts who judged “appropriateness, clarity, completeness, question sequence, completion time, [and] overall appearance”. Methods of assessment not reported. (p6)<br><br>Initial testing with QI team leaders from hospitals participating in a QIC (RR 168/237; 71%).	Hypothesis testing: Association between scores on each of the three scales and i) number of process changes made by QI teams and ii) outcomes (perceived success; performance indicators). Mixed support for hypotheses. Associations found between some scale scores, changes made and perceived success, but not with performance indicators.<br><br>Structure: Three theoretically predicted dimensions were empirically supported (identified by principal component analysis and confirmed using multi-trait multi-item matrix).	Internal consistency: Cronbach’s alpha for all three scales >0.70.<br><br>Stability over time: not assessed.	Feasibility: Instrument was pilot tested by five researchers and judged “straightforward to complete” (p6). Completion time was approximately 10 minutes.<br><br>Acceptability: the number of valid responses was high and missing items low, suggesting “items and response choices are clear and unambiguous” (p8)

**Instrument** (name, index, references for main reports)	**Development & content validity** (item generation; content validity assessments)	**Construct validity**	**Reliability**	**Other assessments** (eg feasibility, acceptability, interpretability)
Organizational Change Manager (OCM)<br><br>Gustafson, 2003 [20, 54]	Factors thought to influence the success of organisational change were identified from interviews with content experts (4 theorists, 3 practitioners) and literature review. In a face-to-face meeting, experts rephrased overlapping factors, eliminated duplicates, and wrote descriptions of levels of each factor (ranging from positive to negative influence on change outcomes). Experts estimated the probability of successful change for each level, from which a Bayesian model was developed.<br><br>Content validity: integrated with development process.<br><br>Initial testing in sample of 198 leaders of QI projects (RR not reported). Subsequent testing in QIC teams (RR 25/34; 73.5%).	Hypothesis testing: Whether scores on the OCM were predictive of i) self-rated success of QI projects, ii) QIC faculty rating of success, and iii) sustainability of outcomes. Based on initial tests, the model was judged to be effective in predicting successful outcomes of improvement projects, but not for predicting sustainability.<br><br>Structure: not assessed	Preliminary test of inter-rater reliability of scores on the OCM.	No other assessments reported.
Organizational readiness to change assessment (ORCA)<br><br>Helfrich, 2009 [21]	Instrument content based on i) interviews with staff involved in implementation projects, to identify factors that facilitation or inhibited intervention implementation and ii) domains from two existing instruments. Items categorised using PARiHS framework.<br><br>Content validity: no independent assessments reported.<br><br>Initial testing in a sample of Veterans Health Administration staff participating in QI projects (RR not reported 80/113 respondents provided complete data for inclusion in analysis). Subsequent administration to representatives from substance use disorder clinics participating in an intervention to introduce preventive care (RR 8/9, 89%) [55].	Hypothesis testing: Association between scale scores and implementation of preventive care. Sample too small to draw conclusions [55].<br><br>Structure: Three theoretically predicted domains from PARiHS framework were empirically supported and most subscales loaded onto the domain they were intended to measure (factors identified by exploratory factor analysis). Two subscales failed to load onto any domain.	Internal consistency: Cronbach's alpha for most scales and subscales > 0.80 threshold set by authors, except for the "Evidence" scale.<br><br>Stability over time: not assessed (single administration)	Acceptability: authors state that a higher proportion of missing items for the third scale may suggest respondent fatigue.<br><br>Feasibility: completion time approximately 15 minutes.
TQManager feedback instrument<br><br>Lageson, 2006 [56]	Instrument content was developed in earlier studies, however the original authors did not report the methods used to identify dimensions or generate items.<br><br>Content validity: no independent assessments reported.<br><br>Administered to a convenience sample of 458 nurses and unlicensed assistive personnel (RR not reported).	Hypothesis testing: no assessments<br><br>Structure: Five theoretical dimensions were described by the developers. Two factors were identified (principal components analysis). The items that loaded on each factor were judged to measure conceptually distinct dimensions.	Internal consistency: Cronbach's alphas for the two subscales was 0.97 and 0.96.	No other assessments reported.

**Instrument** (name, index, references for main reports)	**Development & content validity** (item generation; content validity assessments)	**Construct validity**	**Reliability**	**Other assessments** (eg feasibility, acceptability, interpretability)
Continuous quality improvement questionnaire<br><br>Meurer, 2002 [23, 57]	Initial pool of items derived from systematic review of studies measuring CQI implementation. Items were categorised using domains from quality frameworks.<br><br>Content validity: Eight content experts i) rated items for clarity and how adequately they represented the construct and ii) assigned items to pre-specified domains. Inter-rater agreement across four rounds was used to remove items, reduce the number of domains and assign items to domains.<br><br>Initial testing in sample of hospital managers (RR 200/415, 48%)	Hypothesis testing: i) differences in scale score between hospitals with different demographics (eg. size, region, funding), and ii) association between scale score and a global measure characterising the hospital's QI effort. Most hypotheses not supported.<br><br>Structure: not assessed	Internal consistency: Cronbach's alpha for six of the subscales was >0.70. Two scales <0.70.<br><br>Stability over time: not assessed.	Feasibility: Completion time estimated at 15 minutes.<br><br>No other assessments
Factors influencing success in a QIC	See Schouten, 2010 under 'CQI use' domain – Table 8			
Quality improvement implementation survey II (QIIS)<br><br>Shortell, 2000 [13, 58]	Items were intended to operationalise National Malcolm Baldrige Quality Award criteria. Methods of item generation not reported.<br><br>Content validity: No independent assessments of content validity reported.<br><br>Initial administration to hospital employees (RR 7337/10190; 72% from 61/67 hospitals) [13]. Subsequent administrations to hospital clinicians and administrators (RR 55%, average of 54 respondents per site from 16 sites[58]); and individuals from organisations participating in a QIC evaluation (261 members from 40 QI teams provided data for analyses reported in Shortell 2004 [59]; 359 individuals from 43 organisations provided data for analyses in Lin 2005 [60]).	Hypothesis testing: In hospitals, association between overall QI implementation score and i) perceived impact of QI on human resources development, patient and financial outcomes, ii) LOS and charges, iii) organisational culture type, and iv) process and outcomes of CABG surgery [13, 58]. In QI teams, association between commitment to QI (selected scales) and i) perceived QI team effectiveness, ii) number and depth of changes made to patient care processes, and iii) individual motivation to implement the chronic care model and run PDSA cycles [59-60]. Mixed support for hypotheses.<br><br>Structure: Factor analysis identified seven scales, most comprised of items matching those in the hypothesised dimensions (unpublished data [61]).	Internal consistency: Cronbach's alpha for the seven scales ranged from 0.80 to 0.93 [13, 58-60].<br><br>Stability: not reported	No other assessments reported

Instrument<br>(name, index, references for main reports)	Development & content validity<br>(item generation; content validity assessments)	Construct validity	Reliability	Other assessments<br>(eg feasibility, acceptability, interpretability)
Competing values instrument for organizational culture<br><br>Zammuto, 1991 [13, 62-64]	Item development was not described in any of the included papers. The theoretical basis of the instrument is reported in detail in Zammuto 1991 [62]and Quinn 1991 [63].<br><br>No independent assessments of content validity reported in any of the included papers.<br><br>Original testing of 16 item version in non-healthcare settings (e.g. [62] and [63]). Initial administration of 20 item version adapted for healthcare to hospital employees (RR 7337/10,190; 72% representing 61/67 hospitals) [13]. Subsequent administrations in primary care [42, 48, 65-68] and to QIC participants (261 members from 40 QI teams [59]; 359 individuals from 43 organisations [60]). Testing of 14 item Likert scaled version in healthcare with non-managerial staff across all care settings in the Veteran's Health Affairs (RR 75,135/147,323; 51%) [64].	Hypothesis testing: Association between culture and i) QI implementation, process improvement [59] and motivation for QI [60], ii) process and outcomes of care in CABG surgery [58] and diabetes [68], iii) use of preventive care [67], iv) perceived team effectiveness [59] and team climate [66, 68], v) patient experience of care [12] , vi) perceived practice effectiveness [48], vii) job satisfaction [48], and viii) satisfaction with aspects of medical group practice (e.g. managerial decision making) [65]. Results across studies varied; in some studies most hypotheses were supported [48, 65, 67], in some there was mixed support [58, 68], and in others marginal or no support [12, 59-60].<br><br>Correlations between items and i) other items on the same subscale, and ii) other subscales. Items converged with items from the same subscale; however items from three of the subscales were highly correlated [64].<br><br>Structure: Two, rather than four, factors were empirically supported (principal components analysis and confirmatory factor analysis) [64].	Internal consistency: Cronbach's alpha for the four ipsative scales ranged from 0.46 (rational culture scale, [68]) to 0.82 (group culture scale; [65]). For the Likert scales, Cronbach's alpha ranged from 0.69 (hierarchical culture scale) to 0.85 (entrepreneurial culture scale) [64].<br><br>Stability: not assessed	Pilot testing of the instrument used in the Veterans' Health Affairs all employee survey prompted use of a Likert rather than an ipsative scale, and rewording of two items [64].<br><br>Group culture was consistently identified as the dominant culture type in primary care practices [48, 65-68].<br><br>Additional analyses of data by subgroup have been undertaken by Helfrich, based on which it was concluded: "Culture as measured by the Competing Value Framework varies significantly among employees at different supervisory levels, and other subgroups including by gender, race and clinical profession, and may not be an organisational level construct." [69]
Non health care				
Organizational Change Recipients' Beliefs Scale (OCRBS)	See Armenakis, 2007 under 'individual factors' domain – Table 10			
Readiness for organisational change	See Holt, 2007 under 'individual factors' domain – Table 10			

Table 13: Summary of instrument development and assessment of measurement properties – individual level factors

**Instrument**<br>(name, index, references for main reports)	**Development**<br>(item generation & content validity)	**Construct validity**	**Reliability**	**Other assessments**<br>(eg feasibility, acceptability, interpretability)
Primary care settings				
Satisfaction, perceived skills and impact of CQI participation<br><br>Calomeni, 1999 [70]	Items were based on literature review and learning objectives for training delivered to intervention group. Methods of item generation not reported.<br><br>Content validity: the authors state that the instrument was pre-tested and revised, however details were not reported.<br><br>Instrument administered to nurses responsible for facilitating or leading CQI teams in primary care clinics participating in the intervention group of a randomised trial (RR 13/13, 100%).	Hypothesis testing: none reported.<br><br>Structure: no assessments reported	No assessment of reliability.	No other assessments reported.
Obstacles to the use of CQI	See Geboers, 2001b under 'organisational context' domain – Table 9			
Motivation to run PDSA cycles and implement the CCM<br><br>Lin, 2005 [60, 71]	Items were written to operationalise Vroom's expectancy framework.<br><br>Content validity: no independent assessments reported.<br><br>Instrument was administered to staff from organisations participating in a QI collaborative (RR 80% (n=574) of participants from 43 organisations; 359 respondents provided data suitable for analysis)	Hypothesis testing: Association between individual and composite scale scores and i) culture type, ii) climate, and iii) QI commitment. Most hypotheses supported.<br><br>Structure: no assessments reported	Internal consistency: Cronbach's alpha for the two multi-item scales was 0.90 and 0.94.<br><br>Stability: not assessed	No other assessments reported.
Provider attitude survey (PAS) - CQI scale<br><br>Solberg, 1998b [4]	Items were derived from a longer version of the PAS referenced to an earlier paper. Neither paper reports methods used to generate items.<br><br>Content validity: no independent assessments reported. Unclear if provided in earlier report.<br><br>Administered to all personnel in primary care practices participating in a RT of CQI (RR 647/988, 65.5%)	Hypothesis testing: Association between scale score and presence of a process improvement team. Hypothesis not supported.<br><br>Structure: no assessments reported	Internal consistency: Cronbach's alpha for the final 10 item scale was 0.80<br><br>Stability: not assessed	No other assessments reported.

**Instrument**<br>(name, index, references for main reports)	**Development**<br>(item generation & content validity)	**Construct validity**	**Reliability**	**Other assessments**<br>(eg feasibility, acceptability, interpretability)
Other healthcare settings				
Individual readiness for organizational change<br><br>Cunningham, 2002 [72]	Items were based on questionnaires used to operationalise the stages of change model [73].<br><br>Content validity: no independent assessments reported.<br><br>Administered to a random sample of hospital staff involved in a reengineering program (RR 654/880, 74%). Administered in a separate study to general practice staff participating in a randomised trial of a QI intervention (RR 502/965, 62% from 58 practices) [74].	Hypothesis testing: Association between readiness (scale score) and predictors (e.g. organisational commitment to QI) and outcomes (e.g. participation in reengineering, quality) of readiness. Association between readiness and practice characteristics [74]. Mixed support for hypotheses in both studies.<br><br>Structure: not assessed in [72]. A single factor was identified (principal components analysis); two of six items excluded based on factor loading [74].	Internal consistency: Cronbach's alpha for the single scale was 0.63 in [72] and 0.68 in [74].<br><br>Stability: not assessed	No other assessments reported.
Commitment to change scales<br><br>Herscovitch, 2002a [75-76]	Items were written to operationalise a model of commitment to change [77].<br><br>Content validity: no independent assessments reported.<br><br>Administered to a random sample of nurses (RR 157/600, 26% in study 1 and 108/400, 27% in study 2).	Hypothesis testing: Whether commitment to change (and different forms of commitment) is predictive of behaviours indicating support for change. Most hypotheses supported.<br><br>Structure: Three theoretically predicted dimensions were empirically supported (principal components analysis) and confirmed with data from a subsequent sample (confirmatory factor analysis). Most items loaded as predicted.	Internal consistency: In two studies, Cronbach's alpha for the three scales ranged from 0.71 to 0.94.<br><br>Stability: not assessed	No other assessments reported.<br><br>Low response rate has implications for generalisability of findings.
Behavioural support for change<br><br>Herscovitch, 2002b [75-76]	Items were written for two types of measures, both based on a theory of the behavioral consequences of change.<br><br>Content validity: no independent assessments reported.<br><br>Administered to a random sample of nurses (RR 157/600, 26% in study 1 and 108/400, 27% in study 2).	Hypothesis testing: Whether commitment to change (and different forms of commitment) is predictive of behaviours indicating support for change. Most hypotheses supported.<br><br>Structure: Three theoretically predicted dimensions were not empirically supported (principal components analysis), however the authors judged the items to reflect conceptually different constructs.	Internal consistency: for the three multi-item scales was 0.85, 0.90 and 0.49.<br><br>Stability: not assessed	No other assessments reported.<br><br>Low response rate has implications for generalisability of findings.

Instrument<br>(name, index, references for main reports)	Development<br>(item generation & content validity)	Construct validity	Reliability	Other assessments<br>(eg feasibility, acceptability, interpretability)
Job behaviour related to CQI (JBCQI)<br><br>Irvine, 1995 [78-79]	Items were derived inductively from interviews with hospital staff with experience of CQI teams (n=52). Job behaviours attributable to QI team knowledge and experience were elicited, grouped by theme, and each unique behaviour change was written as a statement. Items with low variation among respondents were removed.<br><br>Content validity: established through inductive item development.<br><br>Administered to a sample comprising interview participants (RR 39/52, 75%). Subsequent administration to CQI teams in 4 hospitals pre- and six months post- CQI introduction (RR time 1: 79/98, 80%; time 2: 57/79, 72%) [79].	Hypothesis testing: Association between JBCQI (scale scores) and i) perceived CQI team success (rated independently by respondents and staff member external to team) and identification with CQI team (predictor of JBCQI), and 2) empowerment (mediating variable). Most hypotheses supported.<br><br>Structure: Four factors were identified (principal components analysis). Most items loaded onto a single factor with a loading above the pre-specified threshold (0.30). Two items that did not load on any factor were eliminated.	Internal consistency: Cronbach's alpha for the four scale ranged from 0.77 to 0.82.<br><br>Stability: not assessed	No other assessments reported.
Empowerment<br><br>Irvine, 1999 [80-81]	Items were derived inductively from interviews with hospital staff with experience of CQI teams (n=52). Perceived changes in self perception attributable to QI team participation were elicited and grouped by theme. Themes reflecting the concept of empowerment were used as the basis for items.<br><br>Content validity: established through review of key theoretical papers and inductive item development.<br><br>Pilot tested in a sample comprising interview participants (RR 52/68, 76%). Subsequent administration to i) staff in 4 hospitals (RR 405/1023, 39.95%) [80] and ii) CQI teams in hospitals pre- and six months post- CQI introduction (RR time 1: 79/98, 80%; time 2: 57/79, 72%) [79].	Hypothesis testing: Association between empowerment (scale scores) and i) perceived CQI team success and identification with CQI team (predictor of empowerment), ii) individual behaviour consistent with CQI principles (outcome), iii) leadership and citizenship behaviours, iv) managerial and non-managerial staff. Mixed support for hypotheses.<br><br>Structure: Three conceptually meaningful factors were identified (principal components analysis). Results were stable in pilot and second sample (n=405).	Internal consistency: Cronbach's alpha for the composite scale was 0.88 [81]<br><br>Stability: not assessed	Ceiling and floor effects assessed.
Non-health care				
Organizational Change Recipients' Beliefs Scale (OCRBS)<br><br>Armenakis, 2007 [82]	A comprehensive review and analysis of theoretical and empirical research identified five types of change recipients' beliefs. Items were developed deductively to measure each of the beliefs.<br><br>Content validity: 19 executives with managerial experience categorised each of the items using the hypothesised categories. Agreement among respondents was determined using Cohen's Kappa.<br><br>Three separate administrations: 1) not-for-profit research company (RR 117/150; 78%); 2) manufacturing employees (RR 117/125, 94%); 3) employees of a public safety company (RR 247/325, 76%)	Hypothesis testing: Association between beliefs and change outcomes, specifically i) organisational justice; ii) commitment to change; iii) organisational cynicism (administration 2). Most hypotheses supported.<br><br>Structure: Five theoretically predicted factors were empirically supported (exploratory and confirmatory factor analysis; data from administrations 2 and 3 respectively).	Internal consistency: Coefficient alpha for the five subscales, across the three studies, ranged from 0.69 to 0.95.<br><br>Stability: not assessed	No other assessments reported.

**Instrument**<br>(name, index, references for main reports)	**Development**<br>(item generation & content validity)	**Construct validity**	**Reliability**	**Other assessments**<br>(eg feasibility, acceptability, interpretability)
Individual TQM orientation<br><br>Coyle-Shapiro, 2003 [83]	Items based on selected literature. Methods of item generation not reported.<br><br>Content validity: no independent assessments reported.<br><br>Administered pre- and post- implementation of TQM in an automotive supply company (RR pre 186/200, 93%; 9 months post 166/186, 89%; 32 months post 118/186, 71%)	Hypothesis tested: Association between TQM orientation and potential predictors of TQM orientation: i) individual factors (organisational commitment, trust in colleagues, higher order need strength) and ii) organisation level factors (management support, supervisor support). Mixed support for hypotheses.<br><br>Structure: Four factors were identified (principal components analysis). Items that cross loaded were eliminated.	Internal consistency: Cronbach's alpha for the four subscales ranged from 0.66 to 0.90.<br><br>Stability: not assessed	No other assessments reported.
Readiness for change<br><br>Hanpachern, 1998 [84]	Items were based on literature (one example references provided). Methods of item generation not reported.<br><br>Content validity: no independent assessments reported.<br><br>Administered to staff in a manufacturing business (RR 131/200, 60%)	Hypothesis testing: Association between readiness and potential predictors of readiness (e.g. job demands, social relations in the workplace, organisational culture). Mixed support for hypotheses.<br><br>Structure: no assessments reported	Internal consistency: Cronbach's alpha for scale 0.82.<br><br>Stability: not assessed	The investigators report that the instrument was "refined in three pilot studies used to improve reliability and to check content and construct validity" (p343), however references or supporting data were not provided.
Readiness for organizational change<br><br>Holt, 2007 [85]	Dimensions and items based on: i) systematic review of literature, ii) thematic analysis of interviews and responses to open-ended questions from 75 managers with recent experience of organisational change, and iii) a survey of 291 managers to rate the importance of themes. Items were written to reflect important themes.<br><br>Content validity: In two rounds, assessors categorised each of the items using the hypothesised dimensions. Items were deleted and new items written to address areas of ambiguity.<br><br>Two separate administrations: 1) Department of Defence employees (RR 264/498, 36%); 2) IT company employees (RR 228/495 46%).	Hypothesis testing: associations between scale scores and i) factors expected to be related to readiness (e.g. work climate), ii) groups expected to differ on readiness, and iii) factors expected to be predicted by readiness (e.g. job satisfaction). Mixed support for hypotheses.<br><br>Structure: Four factors were empirically supported (principal components analysis and confirmatory factor analysis; data from separate administrations). Items from three of the five theoretical factors loaded as predicted. Remaining items loaded on a single factor.	Internal consistency: Cronbach's alpha for the four subscales ranged from 0.66 to 0.94.<br><br>Stability: not assessed	No other assessments reported.

References

1. Apekey TA, McSorley G, Tilling M, Siriwardena AN: **Room for improvement? Leadership, innovation culture and uptake of quality improvement methods in general practice.** *J Eval Clin Pract* 2011, **17:**311-318.
2. Engels Y, van den Hombergh P, Mokkink H, van den Hoogen H, van den Bosch W, Grol R: **The effects of a team-based continuous quality improvement intervention on the management of primary care: a randomised controlled trial.** *Br J Gen Pract* 2006, **56:**781-787.
3. Geboers H, Mokkink H, van Montfort P, van den Hoogen H, van den Bosch W, Grol R: **Continuous quality improvement in small general medical practices: the attitudes of general practitioners and other practice staff.** *Int J Qual Health Care* 2001, **13:**391-397.
4. Solberg LI, Brekke ML, Kottke TE, Steel RP: **Continuous quality improvement in primary care: what's happening?** *Med Care* 1998, **36:**625-635.
5. Solberg LI, Kottke TE, Brekke ML: **Will primary care clinics organize themselves to improve the delivery of preventive services? A randomized controlled trial.** *Prev Med* 1998, **27:**623-631.
6. Solberg LI, Asche SE, Margolis KL, Whitebird RR: **Measuring an organization's ability to manage change: the change process capability questionnaire and its use for improving depression care.** *Am J Med Qual* 2008, **23:**193-200.
7. Solberg LI, Brekke ML, Fazio CJ, Fowles J, Jacobsen DN, Kottke TE, Mosser G, O'Connor PJ, Ohnsorg KA, Rolnick SJ: **Lessons from experienced guideline implementers: attend to many factors and use multiple strategies.** *Jt Comm J Qual Improv* 2000, **26:**171-188.
8. Ingersoll GL, Kirsch JC, Merk SE, Lightfoot J: **Relationship of organizational culture and readiness for change to employee commitment to the organization.** *J Nurs Adm* 2000, **30:**11-20.
9. Barsness ZI, Shortell SM, Gillies EFX, Hughes JL, O'Brien D: **The quality march. National survey profiles quality improvement activities.** *Hosp Health Netw* 1993, **67:**52-55.
10. Weiner BJ, Alexander JA, Shortell SM: **Leadership for quality improvement in health care: empirical evidence on hospital boards, managers, and physicians.** *Med Care Res Rev* 1996, **53:**397-416.
11. Alexander JA, Weiner BJ, Shortell SM, Baker LC: **Does quality improvement implementation affect hospital quality of care?** *Hosp Top* 2007, **85:**3-12.
12. Alexander JA, Weiner BJ, Griffith J: **Quality improvement and hospital financial performance.** *J Organ Behav* 2006, **27:**1003-1029.
13. Shortell SM, O'Brien JL, Carman JM, Foster RW, Hughes EF, Boerstler H, O'Connor EJ: **Assessing the impact of continuous quality improvement/total quality management: concept versus implementation.** *Health Serv Res* 1995, **30:**377-401.

14. Zinn JS, Brannon D, Weech R: **Quality improvement in nursing care facilities: extent, impetus, and impact.** *Am J Med Qual* 1997, **12:**51-61.
15. Zinn JS, Weech RJ, Brannon D: **Resource dependence and institutional elements in nursing home TQM adoption.** *Health Serv Res* 1998, **33:**261-273.
16. Lucas JA, Avi-Itzhak T, Robinson JP, Morris CG, Koren MJ, Reinhard SC: **Continuous quality improvement as an innovation: which nursing facilities adopt it?** *Gerontologist* 2005, **45:**68-77.
17. Weech-Maldonado R, Zinn JE, Hamilton Iii RD: **The performance impact of context in TQM implementation: The nursing facility industry.** *Health Serv Manage Res* 2001, **14:**147-158.
18. Weech-Maldonado R, Zinn JS, Brannon D: **Managerial implications of corporate board involvement and perceived market competition for quality improvement in nursing homes.** *J Healthc Manag* 1999, **44:**382-396.
19. Duckers MLA, Wagner C, Groenewegen PP: **Developing and testing an instrument to measure the presence of conditions for successful implementation of quality improvement collaboratives.** *BMC Health Serv Res* 2008, **8:**172.
20. Gustafson DH, Sainfort F, Eichler M, Adams L, Bisognano M, Steudel H: **Developing and testing a model to predict outcomes of organizational change.** *Health Serv Res* 2003, **38:**751-776.
21. Helfrich CD, Li YF, Sharp ND, Sales AE: **Organizational readiness to change assessment (ORCA): Development of an instrument based on the Promoting Action on Research in Health Services (PARiHS) framework.** *Implement Sci* 2009, **4:**38.
22. Lemieux-Charles L, Murray M, Baker G, Barnsley J, Tasa K, Ibrahim S: **The effects of quality improvement practices on team effectiveness: a mediational model.** *J Organ Behav* 2002, **23:**533-553.
23. Meurer SJ, Rubio DM, Counte MA, Burroughs T: **Development of a healthcare quality improvement measurement tool: results of a content validity study.** *Hosp Top* 2002, **80:**7-13.
24. Schouten L, Grol R, Hulscher M: **Factors influencing success in quality improvement collaboratives: development and psychometric testing of an instrument.** *Implement Sci* 2010, **5:**84.
25. Wilkens R, London M: **Relationships between climate, process, and performance in continuous quality improvement groups.** *J Vocat Behav* 2006, **69:**510-523.
26. Bobiak SN, Zyzanski SJ, Ruhe MC, Carter CA, Ragan B, Flocke SA, Litaker D, Stange KC: **Measuring practice capacity for change: a tool for guiding quality improvement in primary care settings.** *Qual Manag Health Care* 2009, **18:**278-284.
27. Book S, Hellstrom A, Olsson J: **Perceptions of improvement work in Swedish health care: implications for improvement practices.** *Qual Manag Health Care* 2003, **12:**217-224.
28. Olsson J, Kammerlind P, Thor J, Elg M: **Surveying improvement activities in health care on a national level--the Swedish internal collaborative strategy and its challenges.** *Qual Manag Health Care* 2003, **12:**202-216.

29. Kralewski J, Dowd BE, Kaissi A, Curoe A, Rockwood T: **Measuring the culture of medical group practices.** *Health Care Manage Rev* 2005, **30:**184-193.

30. Curoe A, Kralewski J, Kaissi A: **Assessing the cultures of medical group practices.** *J Am Board Fam Pract* 2003, **16:**394-398.

31. Kralewski JE, Wingert TD, Barbouche MH: **Assessing the culture of medical group practices.** *Med Care* 1996, **34:**377-388.

32. Leykum L, Palmer R, Lanham H, Jordan M, McDaniel R, Noel P, Parchman M: **Reciprocal learning and chronic care model implementation in primary care: results from a new scale of learning in primary care settings.** *BMC Health Serv Res* 2011, **11:**44.

33. Marsden PV, Landon BE, Wilson IB, McInnes K, Hirschhorn LR, Ding L, Cleary PD: **The reliability of survey assessments of characteristics of medical clinics.** *Health Serv Res* 2006, **41:**265-283.

34. Deo S, McInnes K, Corbett CJ, Landon BE, Shapiro MF, Wilson IB, Cleary PD: **Associations between organizational characteristics and quality improvement activities of clinics participating in a quality improvement collaborative.** *Med Care* 2009, **47:**1026-1030.

35. Ohman-Strickland PA, John Orzano A, Nutting PA, Perry Dickinson W, Scott-Cawiezell J, Hahn K, Gibel M, Crabtree BF: **Measuring organizational attributes of primary care practices: development of a new instrument.** *Health Serv Res* 2007, **42:**1257-1273.

36. Orzano AJ, Ohman-Strickland PA, Patel M: **What can family medicine practices do to facilitate knowledge management?** *Health Care Manage Rev* 2008, **33:**216-224.

37. Hung DY, Rundall TG, Crabtree BF, Tallia AF, Cohen DJ, Halpin HA: **Influence of Primary Care Practice and Provider Attributes on Preventive Service Delivery.** *Am J Prev Med* 2006, **30:**413-422.

38. Hudson SV, Ohman-Strickland P, Ferrante JM, Lu-Yao G, Orzano AJ, Crabtree BF: **Prostate-specific antigen testing among the elderly in community-based family medicine practices.** *J Am Board Fam Med* 2009, **22:**257-265.

39. Ohman-Strickland PA, Orzano AJ, Hudson SV, Solberg LI, Diciccio-Bloom B, O'Malley D, Tallia AF, Balasubramanian BA, Crabtree BF: **Quality of diabetes care in family medicine practices: Influence of nurse-practitioners and physician's assistants.** *Ann Fam Med* 2008, **6:**14-22.

40. Parker VA, Wubbenhorst WH, Young GJ, Desai KR, Charns MP: **Implementing quality improvement in hospitals: the role of leadership and culture.** *Am J Med Qual* 1999, **14:**64-69.

41. Charbonneau A, Parker V, Meterko M, Rosen AK, Kader B, Owen RR, Ash AS, Whittle J, Berlowitz DR: **The relationship of system-level quality improvement with quality of depression care.** *Am J Manag Care* 2004, **10:**846-851.

42. Goldzweig CL, Parkerton PH, Washington DL, Lanto AB, Yano EM: **Primary Care Practice and Facility Quality Orientation: Influence on Breast and Cervical Cancer Screening Rates.** *Am J Manag Care* 2004, **10:**265-272.

43. Rushmer RK, Kelly D, Lough M, Wilkinson JE, Greig GJ, Davies HT: **The Learning Practice Inventory: diagnosing and developing Learning Practices in the UK.** *J Eval Clin Pract* 2007, **13:**206-211.

44. Kelly DR, Lough M, Rushmer R, Wilkinson JE, Greig G, Davies HTO: **Delivering feedback on learning organization characteristics--using a Learning Practice Inventory.** *J Eval Clin Pract* 2007, **13:**734-740.

45. Kelly DR, Crossley J, Lough M, Rushmer R, Greig GJ, Davies HT: **Diagnosing a learning practice: the validity and reliability of a learning practice inventory.** *Qual Saf Health Care* 2011, **20:**209-215.

46. Shortell SM, Rousseau DM, Gillies RR, Devers KJ, Simons TL: **Organizational assessment in intensive care units (ICUs): construct development, reliability, and validity of the ICU nurse-physician questionnaire.** *Med Care* 1991, **29:**709-726.

47. Hall CB, Tennen H, Wakefield DB, Brazil K, Cloutier MM: **Organizational assessment in paediatric primary care: development and initial validation of the primary care organizational questionnaire.** *Health Serv Manage Res* 2006, **19:**207-214.

48. Brazil K, Wakefield DB, Cloutier MM, Tennen H, Hall CB: **Organizational culture predicts job satisfaction and perceived clinical effectiveness in pediatric primary care practices.** *Health Care Manage Rev* 2010, **35:**365-371.

49. Cloutier MM, Wakefield DB, Tsimikas J, Hall CB, Tennen H, Brazil K: **Organizational attributes of practices successful at a disease management program.** *J Pediatr* 2009, **154:**290-295.

50. Stevenson K, Baker R: **Investigating organisational culture in primary care.** *Qual Prim Care* 2005, **13:**191-200.

51. Stevenson K: **Are your practices resistant to changing their clinical culture?** *Primary Care Report* 2000, **2:**19-20.

52. Sylvester S: **Measuring the learning practice: diagnosing the culture in general practice.** *Qual Prim Care* 2003, **11:**29-40.

53. Duckers M, Spreeuwenberg P, Wagner C, Groenewegen P: **Exploring the black box of quality improvement collaboratives: modelling relations between conditions, applied changes and outcomes.** *Implement Sci* 2009, **4:**74.

54. Molfenter T, Gustafson D, Kilo C, Bhattacharya A, Olsson J: **Prospective evaluation of a Bayesian model to predict organizational change.** *Health Care Manage Rev* 2005, **30:**270-279.

55. Hagedorn HJ, Heideman PW: **The relationship between baseline Organizational Readiness to Change Assessment subscale scores and implementation of hepatitis prevention services in substance use disorders treatment clinics: A case study.** *Implement Sci* 2010, **5**.

56. Lageson C: **Brief report: reliability and validity of the TQManager feedback instrument.** *J Healthc Qual* 2006, **28:**45-49.

57. Meurer SJ, Counte MA, Rubio DM, Arrington B: **Measuring a hospital's ability to improve.** *Am J Med Qual* 2004, **19:**214-222.

58. Shortell SM, Jones RH, Rademaker AW, Gillies RR, Dranove DS, Hughes EF, Budetti PP, Reynolds KS, Huang CF: **Assessing the impact of total quality management and organizational culture on multiple outcomes of care for coronary artery bypass graft surgery patients.** *Med Care* 2000, **38:**207-217.

59. Shortell SM, Marsteller JA, Lin M, Pearson ML, Wu SY, Mendel P, Cretin S, Rosen M: **The role of perceived team effectiveness in improving chronic illness care.** *Med Care* 2004, **42:**1040-1048.

60. Lin MK, Marsteller JA, Shortell SM, Mendel P, Pearson M, Rosen M, Wu SY: **Motivation to change chronic illness care: results from a national evaluation of quality improvement collaboratives.** *Health Care Manage Rev* 2005, **30:**139-156.

61. Shortell SM: **Assessing the implementation and impact of clinical quality improvement efforts(CABG-THR).** [http://shortellresearch.berkeley.edu/]

62. Zammuto RF, Krakower JY: **Quantitative and qualitative studies of organizational culture.** *Res Organ Chan Dev* 1991, **5:**83-114.

63. Quinn R, Spreitzer G: **The psychometrics of the Competing Values Culture Instrument and an analysis of the impact of organizational culture on quality of life.** In *Research in organizational change and development. Volume* 5. Edited by Woodman R, Pasmore W. Greenwich, CT: JAI Press; 1991: 115-142

64. Helfrich CD, Li YF, Mohr DC, Meterko M, Sales AE: **Assessing an organizational culture instrument based on the Competing Values Framework: exploratory and confirmatory factor analyses.** *Implement Sci* 2007, **2:**13.

65. Zazzali JL, Alexander JA, Shortell SM, Burns LR: **Organizational Culture and Physician Satisfaction with Dimensions of Group Practice.** *Health Serv Res* 2007, **42:**1150-1176.

66. Hann M, Bower P, Campbell S, Marshall M, Reeves D: **The association between culture, climate and quality of care in primary health care teams.** *Fam Pract* 2007, **24:**323-329.

67. Hung DY, Rundall TG, Tallia AF, Cohen DJ, Halpin HA, Crabtree BF: **Rethinking prevention in primary care: applying the chronic care model to address health risk behaviors.** *Milbank Q* 2007, **85:**69-91.

68. Bosch M, Dijkstra R, Wensing M, van der Weijden T, Grol R: **Organizational culture, team climate and diabetes care in small office-based practices.** *BMC Health Serv Res* 2008, **8:**180.

69. Helfrich CD: **Measurement equivalence/invariance analysis of organizational culture** [http://www.hsrd.research.va.gov/research/abstracts.cfm?Project_ID=2141699591]

70. Calomeni CA, Solberg LI, Conn SA: **Nurses on quality improvement teams: how do they benefit?** *J Nurs Care Qual* 1999, **13:**75-90.

71. Cretin S, Shortell SM, Keeler EB: **An evaluation of collaborative interventions to improve chronic illness care. Framework and study design.** *Eval Rev* 2004, **28:**28-51.

72. Cunningham CE, Woodward CA, Shannon HS, MacIntosh J, Lendrum B, Rosenbloom D, Brown J: **Readiness for organizational change: A longitudinal study of workplace, psychological and behavioural correlates.** *J Occup Organ Psychol* 2002, **75:**377-392.

73. Prochaska JO, Velicer WF, Rossi S, Goldstein MG, Marcus BH, Rakowski V, Fiore C, Harlow Ll, Redding CA, Rosenbloom D, Rossi SR: **Stages of change and decisional balance for 12 problem behaviors.** *Health Psychol* 1994, **13:**39-46.

74. Christl B, Harris MF, Jayasinghe UW, Proudfoot J, Taggart J, Tan J: **Readiness for organisational change among general practice staff.** *Qual Saf Health Care* 2010.

75. Herscovitch L, Meyer JP: **Commitment to organizational change: extension of a three-component model.** *J Appl Psychol* 2002, **87:**474-487.

76. Meyer JP, Srinivas ES, Lal JB, Topolnytsky L: **Employee commitment and support for an organizational change: Test of the three-component model in two cultures.** *J Occup Organ Psychol* 2007, **80:**185-211.

77. Meyer JP, Herscovitch L: **Commitment in the workplace: Toward a general model.** *Hum Resour Manage R* 2001, **11:**299-326.

78. Irvine D: **The development of measures of organizational citizenship behaviour and changes in job behaviours related to quality management in health care.** *Health Serv Manage Res* 1995, **8:**143-161.

79. Irvine DM, Leatt P, Evans MG, Baker GR: **The behavioural outcomes of quality improvement teams: the role of team success and team identification.** *Health Serv Manage Res* 2000, **13:**78-89.

80. Irvine D, Leatt P, Evans MG, Baker RG: **Measurement of staff empowerment within health service organizations.** *J Nurs Meas* 1999, **7:**79-96.

81. Irvine Doran DM, Baker GR, Murray M, Bohnen J, Zahn C, Sidani S, Carryer J: **Achieving clinical improvement: an interdisciplinary intervention.** *Health Care Manage Rev* 2002, **27:**42-56.

82. Armenakis AA, Bernerth JB, Pitts JP, Walker HJ: **Organizational Change Recipients' Beliefs Scale: Development of an Assessment Instrument.** *J Appl Behav Sci* 2007, **43:**481-505.

83. Coyle-Shapiro JAM, Morrow PC: **The role of individual differences in employee adoption of TQM orientation.** *J Vocat Behav* 2003, **62:**320-340.

84. Hanpachern C, Hanpachern C, Morgan GA, Griego OV: **An Extension of the Theory of Margin: A Framework for Assessing Readiness for Change.** *Hum Resour Dev Q* 1998, **9:**339-350.

85. Holt DT, Armenakis AA, Field HS, Harris SG: **Readiness for Organizational Change.** *J Appl Behav Sci* 2007, **43:**232-255.
